# Unraveling the Strategies Used by the Underexploited Amaranth Species to Confront Salt Stress: Similarities and Differences With Quinoa Species

**DOI:** 10.3389/fpls.2021.604481

**Published:** 2021-02-10

**Authors:** Yanira Estrada, Amanda Fernández-Ojeda, Belén Morales, José M. Egea-Fernández, Francisco B. Flores, María C. Bolarín, Isabel Egea

**Affiliations:** ^1^Department of Stress Biology and Plant Pathology, Centro de Edafologia y Biologia Aplicada del Segura, Consejo Superior de Investigaciones Científicas, Campus Universitario de Espinardo, Murcia, Spain; ^2^Department of Plant Biology, University of Murcia, Murcia, Spain

**Keywords:** osmotic stress, ionic stress, Na^+^ homeostasis, K^+^ homeostasis, Na^+^ transporter genes, seed yield

## Abstract

Yield losses due to cultivation in saline soils is a common problem all over the world as most crop plants are glycophytes and, hence, susceptible to salt stress. The use of halophytic crops could be an interesting alternative to cope with this issue. The Amaranthaceae family comprises by far the highest proportion of salt-tolerant halophytic species. Amaranth and quinoa belong to this family, and their seeds used as pseudo-cereal grains have received much attention in recent years because of their exceptional nutritional value. While advances in the knowledge of salt tolerance mechanisms of quinoa have been remarkable in recent years, much less attention was received by amaranth, despite evidences pointing to amaranth as a promising species to be grown under salinity. In order to advance in the understanding of strategies used by amaranth to confront salt stress, we studied the comparative responses of amaranth and quinoa to salinity (100 mM NaCl) at the physiological, anatomical, and molecular levels. Amaranth was able to exhibit salt tolerance throughout its life cycle, since grain production was not affected by the saline conditions applied. The high salt tolerance of amaranth is associated with a low basal stomatal conductance due to a low number of stomata (stomatal density) and degree of stomata aperture (in adaxial surface) of leaves, which contributes to avoid leaf water loss under salt stress in a more efficient way than in quinoa. With respect to Na^+^ homeostasis, amaranth showed a pattern of Na^+^ distribution throughout the plant similar to glycophytes, with the highest accumulation found in the roots, followed by the stem and the lowest one detected in the leaves. Contrarily, quinoa exhibited a Na^+^ includer character with the highest accumulation detected in the shoots. Expression levels of main genes involved in Na^+^ homeostasis (*SOS1*, *HKT1s*, and *NHX1*) showed different patterns between amaranth and quinoa, with a marked higher basal expression in amaranth roots. These results highlight the important differences in the physiological and molecular responses of amaranth and quinoa when confronted with salinity.

## Introduction

The interest in Amaranth (*Amaranthus* spp.) has greatly increased in recent years. Some species are consumed as vegetables and others as grain-producing crops, being the most important species of these last group *Amaranthus cruentus*, *Amaranthus caudatus*, and *Amaranthus hypochondriacus*. These species are recognized by their high protein content and have been proven to be non-allergenic food with seeds of remarkable nutraceutical properties (Castrillón-Arbeláez and Délano-Frier, 2011). Due to the high quality of amaranth proteins, crops such as corn, wheat, and potatoes have been transformed with genes coding for amaranth seed storage proteins in order to increase their nutritional value and content of essential amino acids (Tamás et al., 2009; Chakraborty et al., 2010). Current interest in amaranth plants is also related to their extraordinary adaptability to grow under abiotic stress conditions, such as heat (Maughan et al., 2009), drought (Huerta-Ocampo et al., 2011), and salinity (Huerta-Ocampo et al., 2014). The high tolerance to abiotic stress has been associated with their ability to grow long tap roots and maintain water absorption under stress (Caselato-Sousa and Amaya-Farfán, 2012). In sum, amaranth may be an agronomic alternative for semiarid and arid areas where other crops produce poor quality grain with low yield or where they are unable to grow.

Yield loss due to salinity is a common problem all over the world as most crop plants are glycophytes and, hence, unable to uphold yield under salinity. For this reason, the interest of halophytes for agriculture in saline conditions has greatly increased in recent years (Panta et al., 2014; Cheeseman, 2015; Flowers and Colmer, 2015). In this aspect, Quinoa (*Chenopodium quinoa*) is one of the very few halophyte seed crops where numerous experiments have been carried out under conditions of salinity (Bazile et al., 2016), although many questions still remain to be clarified (Ruiz et al., 2016; Kiani-Pouya et al., 2019, 2020; Manaa et al., 2019). Contrarily, in amaranth, analogous studies are much scarcer, and what is more, very few comparative studies have been approached between both species, which share the same geographical origin (the Andean region in South America) and belong to the same family (*Amaranthaceae*). It is an exciting perspective to advance in the knowledge of the salt tolerance mechanisms in species such as amaranth and quinoa, since the high genetic variability of both species constitutes an attractive feature for their adaptation to most of the arable regions from tropical to temperate climates, under different environmental conditions (Tang and Tsao, 2017; Kiani-Pouya et al., 2019).

The mechanisms responsible for salinity tolerance imply both tolerance to osmotic stress and ionic stress. Osmotic stress causes inhibition of water uptake as a consequence of increased salt content in the soil around the roots (Munns and Tester, 2008). One key mechanism to cope with osmotic stress is osmotic adjustment, in which plants accumulate inorganic and organic solutes to reduce osmotic potential and maintain water uptake under the stressful condition, being the accumulation of saline ions as the most energy-efficient strategy (Munns et al., 2020). Other important plant mechanisms to confront osmotic stress are those directed to reduce transpirational water loss, which depends not only on stomatal closure but also on stomatal density (Shabala et al., 2013; Albaladejo, 2017).

Ionic stress is the specific component of salt stress that plants need to face when growing in saline soils. In this regard, halophytes are generally considered as Na^+^-includers, that is, the salt tolerance is associated to high Na^+^ accumulation in leaves. However, it is necessary to consider that mechanisms to tolerate potentially toxic levels of Na^+^ in leaf tissues are efficient up to a certain salinity level, until the tolerance limit to cytoplasmic Na^+^ is exceeded, as these mechanisms are rather similar in glycophytes and halophytes (Flowers et al., 2010). Thus, varieties with includer and excluder characters have been identified in quinoa despite exhibiting this species leaves with epidermal bladder cells (EBCs), specialized cells that have a key role as salt sinks for external sequestration of Na^+^ (Shabala et al., 2013). In addition to particular anatomical features, ion homeostasis is maintained by membrane transporters like SOS1 (Salt Overly Sensitive 1), which extrudes Na^+^ out of the root and facilitates its loading into the xylem, and HKT1s (High-affinity K^+^ transporter 1), involved in Na^+^ retrieval from the xylem under salt stress, as well as NHX1 (Na^+^/H^+^ exchanger 1), involved in the vacuolar Na^+^ compartmentation (Munns et al., 2012; Nieves-Cordones et al., 2016; Jaime-Pérez et al., 2017). Na^+^ accumulation causes an important alteration in K^+^ homeostasis, in such a manner that the Na^+^/K^+^ ratio has been considered as a salt tolerance rate index not only in glycophytes but also in halophytes (Cai and Gao, 2020; Kiani-Pouya et al., 2020). Therefore, K^+^ transporters may be key determinants of salt tolerance, which include KT, HAK, or KUP (KT/HAK/KUP family). Thus, quinoa *CqHAK5*-like transporter drives K^+^ influx into EBCs of the leaf to contribute to osmotic balance of cytosol against osmotic pressure due to the salt-containing vacuoles (Böhm et al., 2018). The SKOR transporter (Stellar K^+^ Outward-Rectifying) is also important as it is involved in the long-distance K^+^ transport from root to shoot, toward the xylem vessels (Nieves-Cordones et al., 2016). Research is under way to elucidate the importance of quinoa subjected to salt stress of these transporters involved in Na^+^ and K^+^ homeostasis, although important advances have been already achieved (Ali et al., 2020). Undoubtedly, it is of great interest to fulfill a similar study in amaranth, where, up to our knowledge, neither Na^+^ nor K^+^ transporters have been studied to date.

Most studies on salt tolerance of halophytes are conducted at very high salinity levels (higher than 300 mM NaCl) and with short times of salt exposure, conditions that are not agronomically realistic. Therefore, if we are interested in identifying salt tolerance mechanisms and selecting useful traits with the goal of upholding grain production, lower salt levels should be applied, and salt tolerance evaluated through the whole plant life cycle. This study has been carried out to investigate the comparative mechanisms used by amaranth and quinoa when a not so high salt level is applied (100 mM of NaCl), which warrants seed production, a key agronomic trait. Here we show the different anatomical, physiologic, and molecular responses in amaranth and quinoa when confronted to such salinity level, as well as the expression levels of the main genes involved in Na^+^ and K^+^ homeostasis in amaranth, which could be the determinant of the salt tolerance of this species.

## Materials and Methods

### Plant Material, Growth, and Salt Treatment

In a first screening for salt tolerance carried out with different accessions of amaranth (*Amaranthus caudatus*) (Kwicha Perú, Blanco, Oscar Blanco, Kwicha Granada, and Burganda), and quinoa (*Chenopodium quinoa*) (QQ74, Cherry Vainilla, Inca, and Cahuil), it was found that Kwicha Perú (K1) was the most tolerant amaranth variety to salinity, being able to maintain vegetative development when exposed to extremely high levels of salinity (300 mM of NaCl for 11 days) (Supplementary Figures 1–3). Similarly, it was found that the QQ74 (QQ) variety of quinoa showed the lowest water loss when exposed to 100 mM of NaCl for 20 days (Supplementary Figure 4). Therefore, the K1 variety of amaranth and the QQ variety of quinoa were selected to further study their response mechanisms to salt stress.

Seeds of the selected varieties of amaranth (K1) and quinoa (QQ) were germinated in darkness, in an 8:3 (v/v) mixture of peat and perlite, respectively, at 28°C temperature and 90% of relative humidity (RH). After emergence, plants were transferred to a pot (14 cm diameter) and grown in a controlled-condition growth chamber with 16 h light and 8 h darkness photoperiod, with the light of a photosynthetic photon flux (400–700 nm) of 350 μmol m^–2^s^–1^ at the plant level provided by fluorescent tubes (Philips Master TL-D 58 W/840 REFLEX, Holland), and 18–25°C and 50–60% of temperature and RH, respectively.

To unravel the strategies displayed by amaranth to confront salt stress compared to quinoa in terms of maintenance of vegetative development, a short-term salt treatment assay was carried out in a controlled-condition culture chamber. After 35 days of germination, nine plants of each species were irrigated with half-strength Hoagland solution (Hoagland and Arnon, 1950) as control condition, while another nine plants per species were subjected to salt stress by adding 100 mM NaCl to the Hoagland solution. The salt treatment was applied for 20 days, a sufficient period of treatment for both the osmotic and ion toxicity effects induced by salinity being clearly observed but senescence symptoms not being very evident yet (Campos et al., 2016). This salt stress treatment was selected in order to be near saline stress conditions that occur in the natural environment. Physiological measurements of the plants were carried out at the end of the assay (20 days), and assessment of shoot and root biomass accumulation (g of fresh weight), calculated by weighting individual plants, separating shoots from roots. Root/shoot ratio was calculated by dividing root fresh weight by shoot fresh weight of each individual plant. Finally, vegetative material (adult leaves and stem from the upper half of the plant, and roots) were harvested for further analysis.

To evaluate salt tolerance of amaranth and quinoa in terms of grain yield, a long-term salt treatment assay was carried out in a polyethylene greenhouse with natural conditions located in Santomera municipality (Murcia, Spain) in an area of semiarid conditions typical of South-east Spain. The conditions of the greenhouse were the following: in spring, 40/15°C day/night temperatures, 48% relative humidity, and 600 mmol m^–2^s^–1^ of mean natural light irradiance (photosynthetically active radiation, PAR); and in summer, 50/22°C day/night temperatures, 40% relative humidity, and 1,200 mmol m^–2^s^–1^ of mean natural light irradiance (PAR). At the fifth fully developed leaf stage, 20 plants of each species were transplanted to the greenhouse and grown in containers with coconut peat, using a drip irrigation system and a fertigation solution as described by Egea et al. (2018). The fertigation solution was prepared in 2,000-L tanks with local irrigation water (electrical conductivity = 0.9 dS m^–1^). After 30 days of growing in these conditions, 10 plants per species were subjected to salt treatment with fertigation solution where 100 mM NaCl was added (salt treatment), maintaining the other 10 plants of each species growing in fertigation solution without NaCl (control treatment). When plants reached their physiological maturity, 125 and 110 days after germination for amaranth and quinoa, respectively, irrigation was suppressed, and 15 days later, when plants were fully dried (seed humidity <12%), seeds of primary, secondary, and tertiary (the latter only appeared in quinoa) panicles were harvested, weighted, and finally fully dried in an oven at 60°C.

### Determination of Water Content and Osmotic Potential

Vegetative samples (roots, stems, and adult leaves) of plants from the short-term salt treatment assay were oven-dried at 60°C for 72 h, and water contents (mg g DW^–1^) were calculated as [(FW - DW)/DW], where FW and DW are fresh weight and dry weight, respectively. Root/shoot water content ratio was calculated by dividing root water content (mg g DW^–1^) by shoot water content (mg g DW^–1^) of each individual plant. Shoot water content is the sum of the stems’ and the adult leaves’ water contents.

Osmotic potential was determined by measuring osmolality in the sap extracts of the leaves by the freezing point depression method using an automatic osmometer (Roebling DR 02, Berlin, Germany). Osmolalities (mOsm kg^–1^) were converted to osmotic potential (1 mOsm = -2.408 kPa). Crude leaf sap extracts were obtained by centrifugation of previously frozen material in liquid nitrogen.

### Gas Exchange Parameters Measurements

To analyze stomatal conductance and foliar transpiration, a portable photosynthesis measurement system (CIRAS-2, PP-system, Amesbury, MA 01913, United States) was used in intact upper and fully expanded leaves after 20 days of treatment (control and salt) of adult plants (55 days after germination). The stomatal conductance, *g*_*s*_ (mmol H_2_O m^–2^s^–1^), which indicates the degree of stomatal opening or closing by the rate of CO_2_ entry and H_2_O exit through the stomata, and the foliar transpiration rate, E (mmol H_2_O m^–2^s^–1^) were analyzed. Working conditions of the CIRAS-2 equipment were as follows: 1.7 cm^2^ of measurement leaf surface, 480 ppm reference CO_2_ level, 25.7 to 26.8°C temperature of the leaf in the measurement chamber, 195 ml min^–1^ gas flow rate in the measurement chamber, ambient pressure (1.010 mb) and 500 μmol m^–2^s^–1^ photosynthetic photon flux density flow (PAR).

### Microscopy Analyses

For microscopy analyses, sections of 1 mm^2^ were processed as described by Albaladejo (2017). For observation of stomatal anatomy, six randomly selected digital images, from nine plants per species and treatment, of the adaxial and abaxial surface sections were collected to determine stomatal density (SD, stomata mm^–2^) and stomatal aperture (SA, μm). These parameters were determined using the free ImageJ software (National Institutes of Health^1^). Finally, for leaf tissue anatomical measurements, toluidine blue-stained sections were observed under light microscope (Olympus, Tokyo), and digital images were obtained, 18 randomly selected images, from nine plants per species and treatment, were analyzed using ImageJ software. Mesophyll and bundle sheath cell size (μm^2^) of amaranth, and palisade and spongy parenchyma cell size (μm^2^) of quinoa were measured. Adaxial and abaxial epidermis cell size (μm^2^), leaf thickness (μm), and cell density (cell mm^–2^) were also measured for both species.

### Determination of Na^+^ and K^+^ Ion Content

Dried tissues used for water content determination were milled to powder, digested for 8 h in a concentrated HNO_3_:HClO_4_ (2:1 v/v) solution and analyzed by inductively coupled plasma optical emission spectrometry (ICP-OES) in an ICAP 6500 DUO/IRIS Intrepid II XLD equipment (Thermo Scientific, Waltham, MA, United States). Measurements were carried out at the Ionomics Platform of CEBAS-CSIC (Murcia, Spain).

### Gene Expression Analysis

Reference sequences of amaranth orthologs of the main genes involved in Na^+^ homeostasis, *AhSOS1* (AH006272-RA), *AhHKT1;1* (AHYPO_003592-RA), *AhNHX1* (AHYPO_008765-RA), and in K^+^ homeostasis *AhHAK5* (AHYPO_013402-RA) and *AhSKOR* (AHYPO_001358-RA), were obtained by blasting the mRNA sequences of *CqSOS1*, *CqHKT1;1*, *CqHKT1;2*, and *CqHAK5* (Böhm et al., 2018), and *CqNHX1* (Ruiz-Carrasco et al., 2011) of quinoa, and the mRNA sequence of *AtSKOR* (Pilot et al., 2003) of *Arabidopsis thaliana*, respectively, against the amaranth (*A. hypochondriacus*) genome (Clouse et al., 2016), using Phytozome v13^2^ and Plaza 4.0^3^ plant comparative genomics portals. The reference sequence of the *CqSKOR* gene (AUR62037748-RA) was obtained by blasting the mRNA sequence *AtSKOR* of *A. thaliana* against the quinoa (*C. quinoa*) genome (Jarvis et al., 2017), using the abovementioned plant comparative genomics portals. Phylogenetic trees were generated based on minimal evolution criterion applying the neighbor-joining method with 1,000 times of bootstrap using different species of the Amaranthaceae family, *A. thaliana*, and rice (*Oryza sativa*) (Supplementary Figure 5). Amino acid sequence alignment of *SOS1*, *HKT1s*, *NHXs*, *HAK5*, and *SKOR* orthologs was performed using ClustalW^4^ software (Supplementary Figures 6–10). All accession numbers and species for all amino acid sequences are listed in Supplementary Table 1.

The expression levels of these genes were analyzed for control and salt-treated plants by quantitative real-time PCR (RT-qPCR) after 20 days of treatment. For these analyses, three biological replicates were considered, each one consisting of three plants (*n* = 9). Fresh roots, stems, and the first fully developed leaf were immediately frozen in liquid nitrogen and pulverized in a mortar. Then, 120 mg of the frozen powder was used for total RNA extraction with the E.Z.N.A.^®^ Plant RNA Kit (Omega, Bio-Tek) according to the manufacturer’s instructions. Contaminant DNA was removed with RNase-free DNase Set I (Omega, Bio-Tek). Total RNA was quantified in a Nanodrop 1 ND-2000 spectrophotometer (Thermo Scientific, Waltham, MA, United States), and 1 μg was used for cDNA synthesis with the iTaq^®^ iScript cDNA synthesis kit (Bio-Rad). One microlitre of the cDNA sample was used for gene amplification using the iTaq^®^ Universal SYBR Green Supermix (Bio-Rad). Amplification reactions were carried out in a Rotor Gene 3000 (Corbett Research). All primers used for quantitative RT-qPCR are listed in Supplementary Table 2. Serial dilutions of cDNA samples were used to make a standard curve in order to calculate the amplification efficiency of primers. The presence of a single band on an agarose gel electrophoresis and a single peak in the melting temperature curve confirmed the specificity of RT-qPCR amplification. Relative expression data were calculated as described by Asins et al. (2013) using *Elongation Factor 1a* as housekeeping gene of each species, *AhEF1a* (AH015912-RA) for amaranth and *CqEF1a* for quinoa (Böhm et al., 2018), respectively. The expression level was calculated using the 2^–ΔΔ*Ct*^ method (Livak and Schmittgen, 2001), considering the expression level from the root of amaranth in control conditions as the calibrator sample.

### Determination of Oxidative Status and Total Antioxidant Capacity of Plants

Malondialdehyde (MDA) was quantified in adult leaves, as indicator of cell membrane lipid peroxidation, by means of the thiobarbituric acid reactive substrates (TBARS) assay, using the protocol described by Sánchez-Bel et al. (2012) with slight modifications. Briefly, 0.5 g of leaf-pulverized tissue was homogenized in 4 ml of 0.1% trichloroacetic acid (TCA) solution using a mortar. The homogenate was centrifuged at 14,000 × *g* for 10 min at room temperature, and 0.5 ml of the supernatant was added to 1.5 ml of 0.5% thiobarbituric acid (TBA) in 20% TCA. The mixture was incubated at 90°C in a shaking water bath for 30 min, and the reaction was stopped by placing the reaction tubes in an ice bath. The samples were centrifuged at 14,000 × *g* for 10 min, and the absorbance of the supernatant was read at 532 nm in a spectrophotometer (Helios Gamma Spectrophotometer, Thermo Scientific). The value for non-specific absorption at 600 nm was subtracted. The amount of the MDA–TBA complex (red pigment) was calculated using the extinction coefficient 155 mM^–1^cm^–1^. Results were expressed as nmol MDA produced per gram of fresh weight per hour (nmol MDA g^–1^h^–1^).

In order to evaluate the total antioxidant activity of the plant tissues, the Trolox Equivalent Antioxidant Capacity (TEAC) assay was performed as described by Egea et al. (2007). The ABTS•− radical anion solution was generated by incubating at 60°C for 6 min a mixture of 2.5 mM 2,2′-azobis (2-amidinopropane) hydrochloride (ABAP) and 20 mM 2,2′-azinobis(3-ethylbenzothiazoline-6-sulfonate) ABTS_2_^–^stock solution in 100 ml of phosphate buffer (100 mM sodium phosphate with 150 mM NaCl, pH 7.4). Absorbance at 734 nm was measured to check ABTS•− formation. Fresh leaf, 0.2 g, was grounded in 10 ml in the mentioned above buffer and centrifuged for 10 min at 9,500 rpm at 4°C. Of the sample, 40 μl was mixed with 1.960 μl of the ABTS•− radical solution to measure the antioxidant activity at 734 nm for a period of 6 min. The decrease in absorbance at 734 nm observed after the addition of each compound was used to calculate the Trolox equivalent antioxidant capacity (TEAC). A calibration curve was prepared with increasing concentrations of Trolox (water-soluble compound analogous to vitamin E). The TEAC activity was calculated according to Egea et al. (2007) and represents the concentration of Trolox, in μmol 100 g^–1^ FW, which has the same antioxidant capacity as the analyzed sample (Trolox equivalent).

### Determination of Grain Yield

Seeds (grain) were manually separated using sieves and filters of different pore size to remove the rest of the dry panicle. Total yield (g) was calculated weighting all harvested seeds by panicle (primary, secondary and tertiary). The average seed weight (mg) was calculated weighting 100 seeds per plant (mg 100 seeds/100). The number of seeds per panicle was calculated by dividing the total seed yield (g) between average seed weight.

### Statistical Analysis

Experimental data are presented as mean ± standard error (SE) of 18 plants per species (nine plants per each treatment). Statistical analysis was performed using SPSS 19.0 software package by a two-way analysis of variance (ANOVA) to determinate the interaction between different factors (treatment, species, leaf surface, plant tissue, or panicle type depending on the variable), carrying out a factorial design and a one-way analysis of variance (ANOVA), followed by Tukey’s test (*p* ≤ 0.05) to determinate in each combination of factors where differences were significant, denoted by different letters.

## Results

### Preselection of Salt-Tolerant Varieties During Seed Germination and in Young Plants

Four varieties of *A. caudatus* were analyzed for salt tolerance during seed germination and seedling growth. Compared with the control condition (no salt), only Kwicha Peru (K1) did not reduce the germination percentage when increasing the salinity levels, whereas the rest of the varieties were differently affected. Thus, a sharp inhibition was found in Blanco (B) and Burganda (Bur) varieties starting from 100 mM NaCl, and Oscar Blanco (OB) and Kwicha Granada (K2) varieties at 150 mM NaCl (Supplementary Figure 1). Subsequently, the salt tolerance was evaluated in young plants by applying 200 mM NaCl for 5 days followed by 300 mM NaCl for 6 days more. Visually, it was observed that K1 was the only variety that seems to be not affected by the salt treatment at the end of the assay (Supplementary Figure 2A). The differences among varieties were clearly observed over time, as shoot biomass increased after 5 days of salt treatment (DST) in three varieties (B, K1, and OB), but it only increased in K1 after 11 DST (Supplementary Figure 2B). Regarding leaf chlorophyll contents, again K1 was the variety maintaining chlorophyll levels at 5 DST together with B and the one exhibiting the lowest reduction at 11 DST (Supplementary Figure 2C). Finally, to estimate water loss through the leaf, an important physiological trait to assess salt tolerance of halophytes, weight loss of detached leaves from plants grown in control was monitored at a short term (during 6 h) and after 24 h (Supplementary Figure 3). The varieties B, K1, and OB exhibited a leaf water loss lower than K2 and Bur, which suggests that in the latter two varieties, transpiration is higher in salt stress. By taking together these results, the most tolerant amaranth variety during both seed germination and development of young plants is K1, which was selected for further studies.

In parallel, a preliminary study was also carried out with four varieties of quinoa whose salt responses were unknown (Supplementary Figure 4A). The germination percentages were not reduced by salt treatment (100 mM NaCl) in QQ74 (QQ) and Cherry Vainilla (CV) varieties, it was only slightly reduced in Inca (IN), while in Cahuil (CA), it was reduced by 50% (Supplementary Figure 4B). Therefore, the first two varieties could be good candidates for the comparative study of salt tolerance between amaranth and quinoa. To estimate water loss through the leaves, weight loss of pots was determined during the first and second day of salt stress (100 mM NaCl). The weight loss was significantly lower in QQ than in the other varieties (Supplementary Figure 4C), which suggests that this variety has the highest ability to maintain leaf water content under salt stress. By taking together these results, amaranth variety K1 and quinoa variety QQ were selected for comparison of salinity responses in both species.

### Amaranth and Quinoa Show Similar Growth Responses but Different Osmotic Responses Under 100 mM NaCl Salt Stress

**FIGURE 1:**
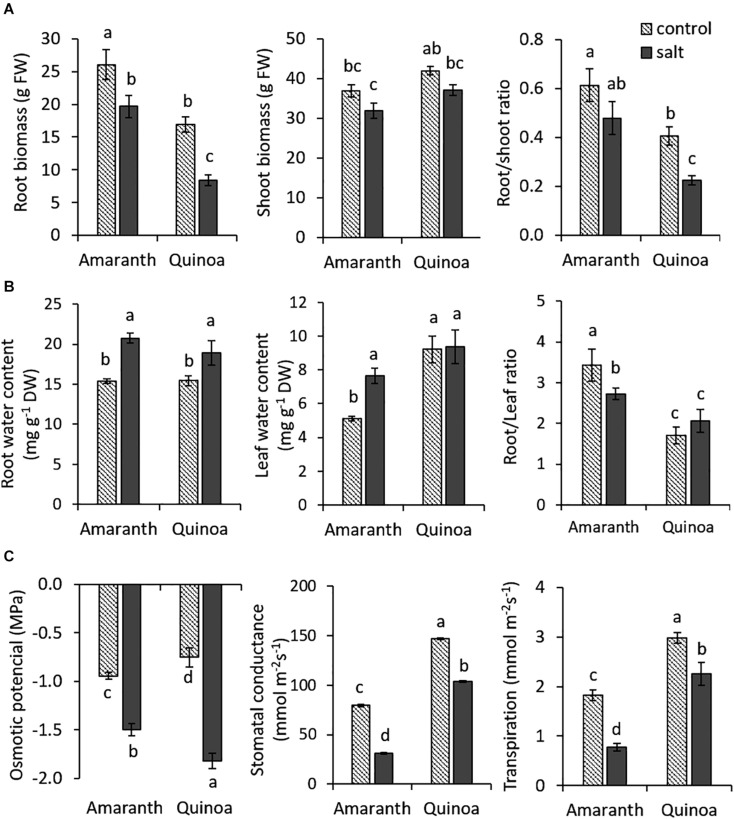
Changes induced by salinity in plant growth and physiological traits related to osmotic homeostasis in amaranth and quinoa. **(A)** Root and shoot biomass and root/shoot ratio in plants grown in control and salt stress (100 mM NaCl) for 20 days. Values are means ± SE (*n* = 9 plants). **(B)** Water contents in roots and adult leaves of amaranth and quinoa plants in control and salt stress (100 mM NaCl) conditions for 20 days and root water content/leaf water content ratio. **(C)** Osmotic potential, stomatal conductance, and transpiration rate in leaves of amaranth and quinoa plants in control and salt stress (100 mM NaCl) conditions for 20 days. Values are means ± SE (*n* = 9). Different letters indicate significant differences between species and treatment (Tukey’s test *p* ≤ 0.05).

When the previously selected varieties of amaranth and quinoa were salt treated (100 mM NaCl) at the eighth developed leaf stage (adult plants) for 20 days, we did not find significant changes in shoot vegetative biomass between the control condition and salt stress. Contrarily, root growth was significantly reduced with salinity in both species, although the reduction was greater in quinoa (50%) than in amaranth (25%) (Figure 1A). The main difference between both species was the root/shoot ratio. Thus, the values of root/shoot ratio in amaranth were 33 and 52% higher in the control and salt stress, respectively, than in quinoa, which indicates that amaranth has a denser root system than quinoa.

Regarding traits related to osmotic homeostasis, first, water contents were analyzed in the root and leaf at the end of the experiment (Figure 1B). These significantly increased in salt treatment with respect to control in roots of both species (25.8% in amaranth and 18.4% in quinoa), but in leaves, only amaranth increased (33%) its water content with salinity, whereas quinoa maintained a similar leaf water content in both treatments. It is also interesting to remark the differences found between the ratios of root/leaf water contents, which are significantly higher in amaranth than in quinoa in the control (50% higher) and salt treatment (24.2% higher) (Figure 1B).

The leaf Ψ_*s*_ reduction induced by salinity was significantly greater in quinoa (59%) than in amaranth (37%) (Figure 1C). Regarding traits involved in leaf water loss, stomatal conductance (*g*_*s*_) and transpiration ratio (E), values of both traits in control condition were significantly higher in quinoa than in amaranth (46.1 and 39%, respectively) (Figure 1C). Under salinity, both species significantly reduced their leaf *g*_*s*_ and *E*-values compared with the control condition, but the reduction percentages were significantly greater in amaranth than in quinoa (around 60% in both traits for amaranth leaves and 25–30% for quinoa leaves).

### Stomatal Density and Stomatal Aperture in Leaves of Amaranth and Quinoa

**FIGURE 2:**
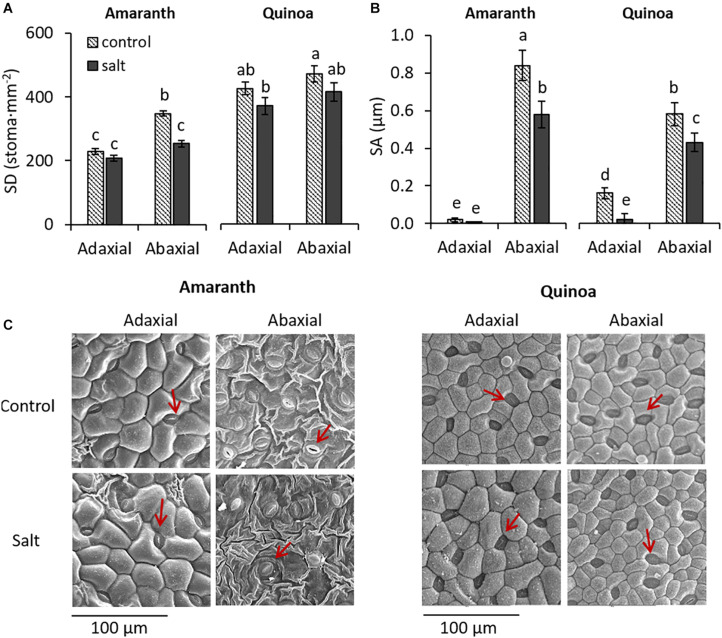
Changes induced by salinity in stomata of amaranth and quinoa. Plants were grown in control and salt stress (100 mM NaCl) for 20 days. **(A)** Stomatal density (SD) and **(B)** stomatal aperture (SA) in abaxial and adaxial surfaces of amaranth and quinoa leaves, from plants in control and salt stress conditions. **(C)** Representative micrographs of leaf abaxial and adaxial epidermal sections showing stomata of both species (red arrows), from plants in control and salt stress conditions. Values are means ± SE of 18 randomly selected images from nine plants per species and treatment. Different letters indicate significant differences between species, treatment, and leaf surface (Tukey’s test, *p* ≤ 0.05).

In amaranth, stomatal density (SD) was lower in the adaxial than in the abaxial surface under control condition, and it was in the abaxial surface where the SD was significantly reduced (27.3%) by salinity, as it is illustrated in the micrographs (Figures 2A,C). In quinoa, however, the SD was similar in both leaf surfaces, and although a tendency to decrease with salinity was observed in both, no significant differences between control and salt treatments were achieved. Regarding SA, the stomata of amaranth were practically closed in the adaxial surface, as shown by the very low values in both control and salt conditions (0.02 and 0.01 μm, respectively), while it was very high in the abaxial surface in the control although it was significantly reduced (31%) under salinity. In quinoa, SA was also much lower in adaxial than in abaxial surfaces, but salinity decreased SA at both surfaces (87.5% in adaxial and 26% in abaxial surface). Moreover, another difference between both species was that SA of abaxial surface both at control and salt conditions was lower in quinoa than in amaranth (31 and 26%, respectively) (Figures 2B,C).

**FIGURE 3:**
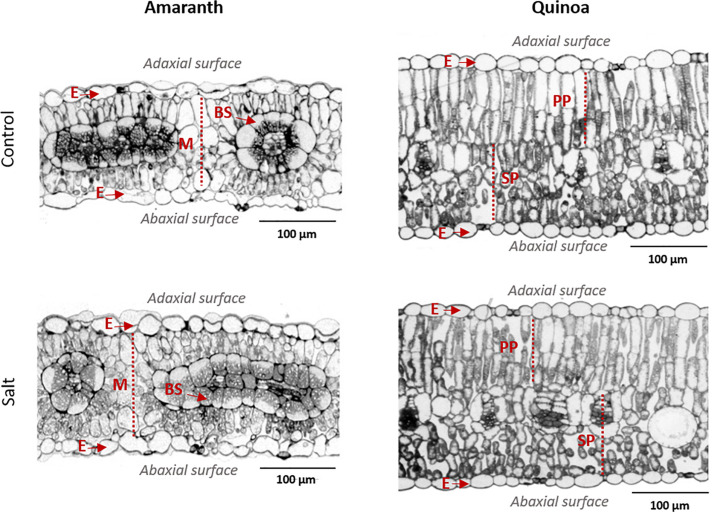
Anatomical differences in leaves of amaranth and quinoa, from plants grown in control and salt stress (100 mM NaCl) for 20 days. Representative light microscopy images of leaf transversal sections: E, epidermis; M, mesophyll; BS, bundle sheath; PP, palisade parenchyma; SP, spongy parenchyma.

The anatomy of amaranth and quinoa leaves is very different, as observed in light microscopy images (Figure 3), which might influence the regulation of water loss (Franco-Navarro et al., 2016). Thus, amaranth showed the typical Kranz type leaf anatomy of C_4_ plants, with the bundle sheath (BS) cells containing centripetally located chloroplasts, and a layer of mesophyll cells surrounding the BS. These cells were those altered by salinity, as mesophyll cells significantly reduced their size with salinity and increased their cell density, while BS cells remain unmodified (Figure 3 and Table 1). Leaves from quinoa showed the typical anatomy of C_3_ plants, where the specialized Kranz type is absent. No morphological alterations were induced by salinity in quinoa leaves (Figure 3 and Table 1).

**TABLE 1:** Anatomical parameters determined by light microscopy in leaves of amaranth and quinoa.

Amaranth	Control	Salt
**Leaf thickness (μm)**	160.6 ± 2.4 a	173.5 ± 4.2 a
**Epidermis**		
*Adaxial cell size* (*μm^2^*)	186.9 ± 13.9 a	205.2 ± 9.6 a
Abaxial cell size (μm^2^)	190.7 ± 11.7 a	222.0 ± 9.6 a
**Mesophyll**		
Cell size (μm^2^)	134.2 ± 6.1 a	71.1 ± 11.6 b
Cell density (cell mm^–2^)	6327 ± 230.4 b	7673 ± 279.9 a
**Bundle sheath**		
Cell size (μm^2^)	290.8 ± 31.6 a	327.2 ± 19.0 a
Cell density (cell mm^–2^)	3203 ± 327.4 a	3606 ± 387.20 a

**Quinoa**	**Control**	**Salt**

**Leaf thickness (μ**m)****	228.2 ± 8.7 a	255.97 ± 10.0 a
**Epidermis**		
Adaxial cell size (μm^2^)	194.0 ± 41.7 a	170.5 ± 8.2 a
Abaxial cell size (μm^2^)	110.5 ± 22.4 a	127.2 ± 10.3 a
**Palisade parenchyma**		
Cell size (μm^2^)	267.5 ± 27.2 a	265.8 ± 2.3 a
Cell density (cell mm^–2^)	3718 ± 111.5 a	3287 ± 219.4 a
**Spongy parenchyma**		
Cell size (μm^2^)	147.0 ± 2.3 a	124.5 ± 9.1 a
Cell density (cell mm^–2^)	5848 ± 231.4 a	6530 ± 471.1 a

*Values are means ± SE of six randomly selected images from nine plants per species and treatment. Different letters indicate significant differences by Tukey’s test between salt and control for each species and cell type (p ≤ 0.05).*

### Changes Induced by Salinity in Oxidative Homeostasis of Leaves

**FIGURE 4:**
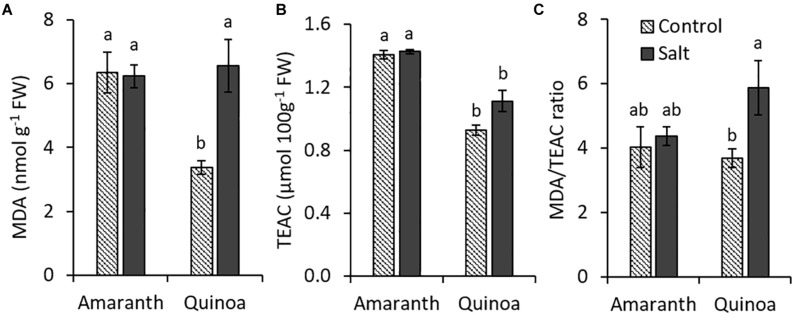
Changes induced by salinity in oxidative homeostasis in amaranth and quinoa. **(A)** Production of malondialdehyde (MDA, an indicator of oxidative status of plants), **(B)** total antioxidant capacity in Trolox equivalents (TEAC) and **(C)** MDA/TEAC ratio in leaves of amaranth and quinoa plants grown in control and salt stress (100 mM NaCl) for 20 days. Values are means ± SE (*n* = 9). Different letters indicate significant differences between species and treatment (Tukey’s test, *p* ≤ 0.05).

We have analyzed MDA accumulation (an indicator of membrane lipid peroxidation) as a marker of oxidative stress of plants, as well as the total antioxidant capacity measured as TEAC in leaves of both species (Figures 4A,B). In amaranth, MDA levels were similar in control and salt-treated plants. In quinoa, however, the MDA content in leaves from plants in the control condition was approximately half of that of amaranth in the same condition and increased in salt stress until achieving a similar level to that of amaranth (Figure 4A). The total antioxidant capacity determined as TEAC was higher in amaranth than in quinoa, and athough a trend to increase it with salinity was observed in both species, especially in quinoa, significant differences were not achieved (Figure 4B). When the MDA/TEAC ratio was calculated, similar values were found for amaranth (in control and salt stress) and for quinoa in control condition, while the ratio significantly increased (37.5%) in salt-treated plants of quinoa (Figure 4C). These results suggest that salinity did not induce changes in oxidative homeostasis in amaranth, so it seems it did not suffer oxidative damage by salt stress.

### Changes Induced by Salinity in the Na^+^ and K^+^ Accumulation

**FIGURE 5:**
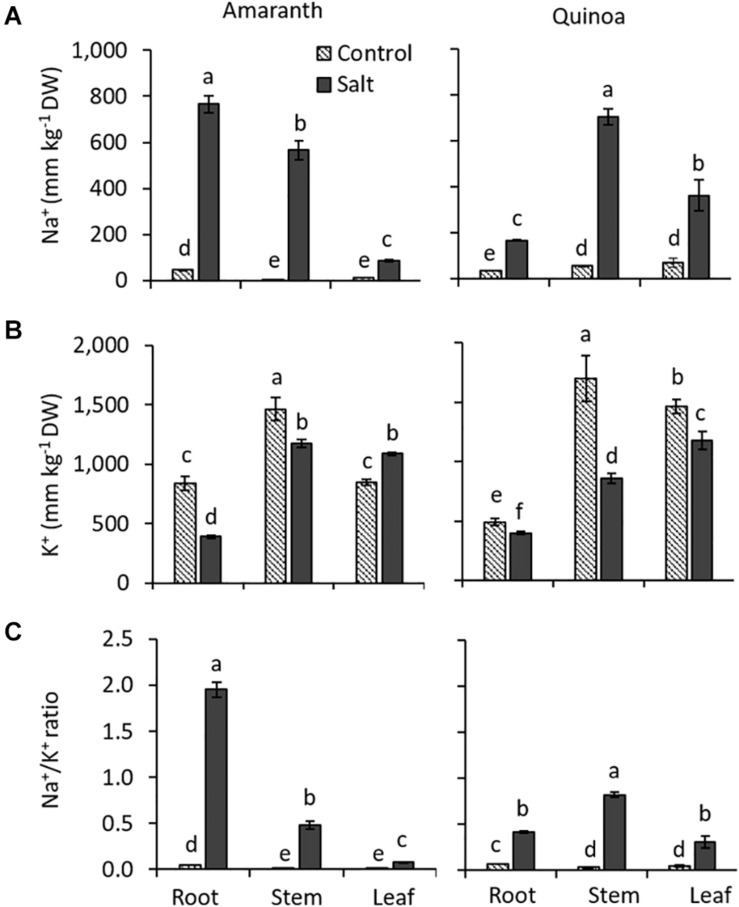
Different changes induced by salinity in Na^+^ and K^+^ accumulation in amaranth and quinoa. **(A)** Na^+^ and **(B)** K^+^ contents, and **(C)** Na^+^/K^+^ ratio analyzed in roots, stem, and leaves of amaranth and quinoa plants grown in control and salt stress (100 mM NaCl) for 20 days. Values are means ± SE (*n* = 9). Different letters indicate significant differences between treatment and plant tissue in each species (Tukey’s test, *p* ≤ 0.05).

Both species showed opposite behaviors regarding Na^+^ distribution within the plant (Figure 5A). In amaranth, the Na^+^ accumulation was higher in the roots, followed by the stem, and it was much lower in the leaf, exhibiting an excluder character. Contrarily, quinoa showed a Na^+^ pattern typical of halophytes, featured by their includer character, since Na^+^ accumulation is much greater in the shoot than in the root, being the accumulation twice higher in the stem than in the leaf.

Generally, Na^+^ accumulation is accompanied by K^+^ reduction, as it was found in the different organs analyzed from amaranth and quinoa salt-treated plants (Figure 5B). Thus, a K^+^ reduction of 50% was observed in salt-treated amaranth root, just the plant organ with higher Na^+^ accumulation, while it increased with salinity in amaranth leaves. An inverse relation seems also to exist in quinoa, where K^+^ reduction was small in the root, followed by the leaf, and showing the highest reduction in the stem. The different behaviors of both species with respect to ion homeostasis is clearly shown in the Na^+^/K^+^ ratio. This was fourfold higher in the root than in the stem of salt-treated plants of amaranth and around 24-fold higher in the root compared to that in leaf. In quinoa, however, the Na^+^/K^+^ ratio was twice higher in the stem than in the root under salinity, but the salt-treated plants maintained low levels of Na^+^/K^+^ ratio in leaves (Figure 5C).

### Expression in Different Plant Organs of Key Genes Involved in Na^+^ and K^+^ Transport

In order to analyze the transcript levels of the main genes involved in uptake and long-distance Na^+^ transport, the previous step was to identify amaranth *AhSOS1*, *AhHKT1s*, and *AhNHX1* orthologs to those of quinoa, *CqSOS1*, *CqHKT1;1*, and *CqHKT1;2*, and *CqNHX1*, respectively (Supplementary Figures 5–10 and Supplementary Table 1). In the case of amaranth *HKT1s*, we found in databases two proteins of this family (AH AHYPO_003592-RA and AH 014429-RA), but only one had homology with quinoa *CqHKT1*;1 and *CqHKT1;2*, the protein AHYPO_003592-RA, which was named *AhHKT1;1* since it is the isoform showing higher homology to the quinoa one (Supplementary Figure 5B and Supplementary Table 1). The other protein of the *HKT1* family (AH 014429-RA) in amaranth had higher homology with *HKT1;3* of *Beta vulgaris* and *HKT1;1* of *Suaeda salsa* and *Salicornia bigelovii*. However, its expression was practically zero in amaranth in the control condition as well as in salt stress, and therefore, it was not included in the gene expression analyses.

**FIGURE 6:**
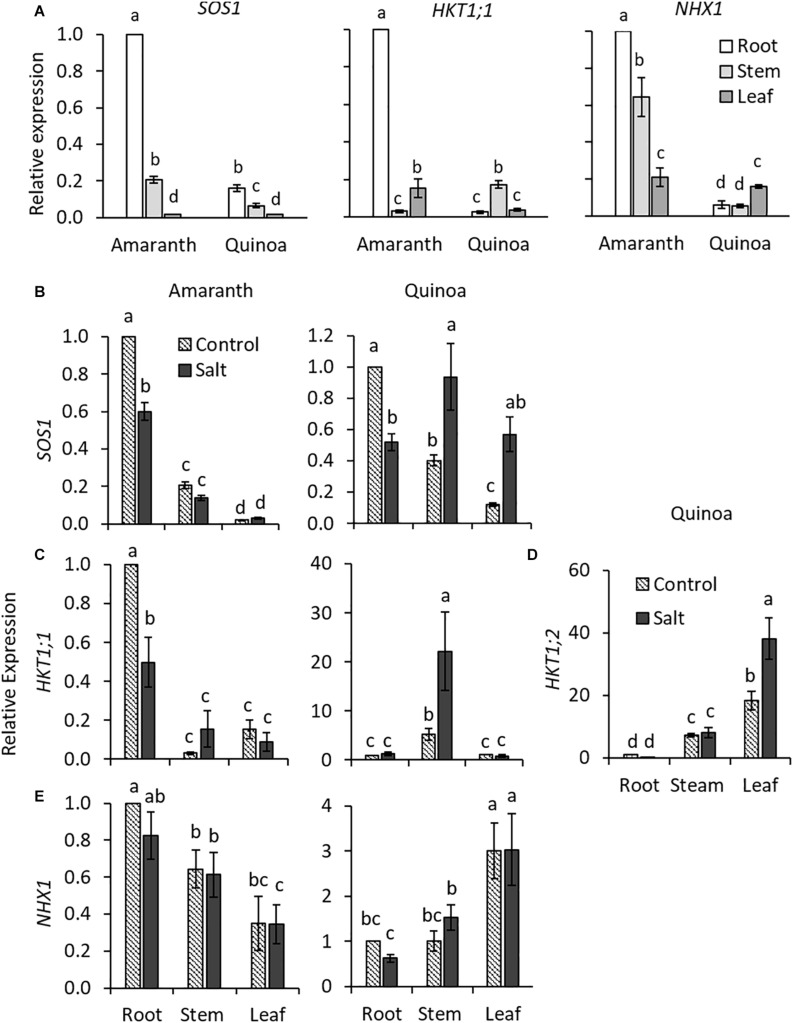
Constitutive and relative expression of genes involved in Na^+^ homeostasis in amaranth and quinoa. **(A)** Comparative constitutive expression of *SOS1*, *HKT1;1*, and *NHX1* genes analyzed in root, stem, and leaf of plants grown in control condition. Relative expression of **(B)** *SOS1*, **(C)** *HKT1;1*, and **(E)** *NHX1* for amaranth and quinoa root, stem, and leaves, and **(D)** *HKT1;2* only for quinoa. Plants were grown in control and salt stress (100 mM NaCl) for 20 days. For constitutive expression, expression in root of amaranth was set to one **(A)**. For relative expression, root expression in control was set to one for each species **(B–E)**. Values are means ± SE of three biological replicates (each one of three plants, *n* = 9). Different letters indicate significant differences among mean values of constitutive expression levels of *SOS1*, *HKT1;1*, and *NHX1* genes **(A)** and between treatment and plant tissue in each species in **(B–E)** (Tukey’s test, *p* ≤ 0.05).

The gene expression levels were analyzed in roots, stem, and leaves of the control and salt-treated plants for 20 days. Constitutively, the expression of *SOS1*, *HKT1;1*, and *NHX1* genes were all much higher in the roots of amaranth than in those of quinoa (Figure 6A). In amaranth, the expression of *SOS1*, involved in Na^+^ extrusion, was downregulated under salinity in roots, where the highest accumulation of Na^+^ was found (Figure 5A), while it was maintained in stem and leaf in similar levels as in control plants (Figure 6B). In quinoa, where the highest Na^+^ accumulation occurred in the shoot (Figure 5A), expression of *SOS1* significantly increased in the stem and especially in the leaf under salinity, while the opposite behavior, a reduction of expression, was observed in the root (Figure 6B). Regarding *HKT1;1* basal expression, which product is involved in transporting Na^+^ from xylem into the cells, the highest expression in amaranth was observed in the root, while in quinoa, it was in the stem (Figure 6A). Salinity induced a reduction in the expression of this gene in the root of amaranth, indicating a reduced Na^+^ uploading from xylem, while a high upregulation of *HKT1;1* expression was observed in the stem of quinoa, indicating a retention of Na^+^ in this tissue (Figure 6C), which is in accordance with the highest Na^+^ accumulation found in the stem of salt-treated plants of quinoa (Figure 5A). In quinoa, the other *HKT1* isoform, *HKT1;2*, was preferentially expressed in leaf, where its expression significantly increases with salinity, indicating that this isoform is mainly responsible for the retention of Na^+^ in this plant organ (Figure 6D). This is in accordance to Na^+^ accumulation found in leaf of quinoa salt-treated plants (Figure 5A). The expression of *NHX1*, involved in Na^+^ accumulation into vacuoles, was constitutively more pronounced in the root and stem of amaranth than in those of quinoa, and salinity did not affect its expression level in any organ of both species (Figure 6E).

Expression of *HAK5*, involved in root K^+^ uptake, and of *SKOR*, involved in the root-to-shoot transport of K^+^ toward the xylem vessels, were analyzed in the roots. The initial step was to identify amaranth *AhHAK5*, ortholog to *CqHAK5* of quinoa, and amaranth *AhSKOR* and quinoa *CqSKOR*, orthologs to *A. thaliana AtSKOR* (Supplementary Figures 5D,E, 9, 10 and Supplementary Table 1). When analyzing gene expressions in salinity, *HAK5* was downregulated in the roots of both species under salt stress, and although *SKOR* was also significantly downregulated in amaranth root under salt stress, the basal level of *SKOR* and *HAK5* expression was much higher in the root of amaranth compared with that of quinoa (Supplementary Figures 11A,B).

### The Long-Term Salt Tolerance Is Maintained in Amaranth

**FIGURE 7:**
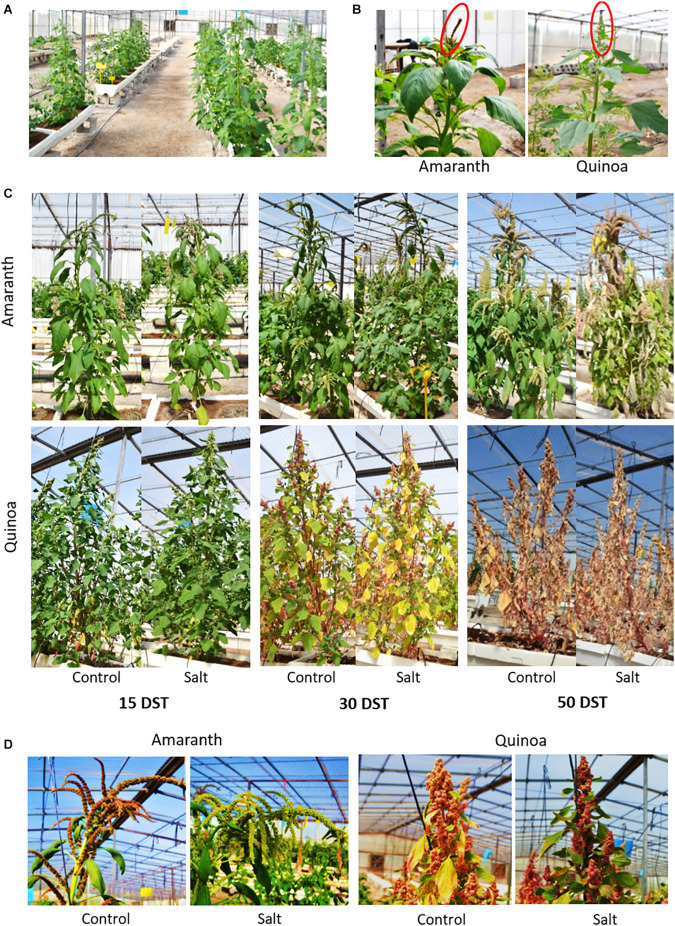
Phenotype of amaranth and quinoa plants through its life cycle in a greenhouse assay. Plants were transferred to a greenhouse at the fifth true leaf stage, and after 1 month, half of them were subjected to salt treatment (100 mM NaCl) for 50 days, whereas the other half was maintained in control condition. **(A)** General view of plants in the greenhouse just before applying salt treatment. **(B)** Details of the main panicles starting to grow at that time (just prior to salt treatment). **(C)** Images of representative phenotypes of amaranth and quinoa plants in control condition and after 15, 30, and 50 days of salt treatment (DST). The physiological maturity of quinoa plants was observed after 30 DST and was fully achieved at 50 DST, while in amaranth this was not observed after that time. **(D)** Representative images of main panicles of amaranth and quinoa, from plants in control condition and salt stress after 30 DST.

Finally, we carried out a long-term experiment in greenhouse to determine the salt tolerance on the basis of seed yield (Figure 7A). Here the salt treatment was applied just when the main panicle was beginning to grow (Figure 7B). It is interesting to point out the different phenotypic changes observed in amaranth and quinoa plants through their life cycle, as shown in representative images of plants after 15, 30, and 50 DST (Figure 7C). Thus, while no remarkable phenotypic changes were observed in amaranth, quinoa plants started to exhibit physiological maturity from 30 DST onward, which was reflected in the main panicles (Figure 7D), and this was completely achieved at 50 DST (Figure 7C).

**FIGURE 8:**
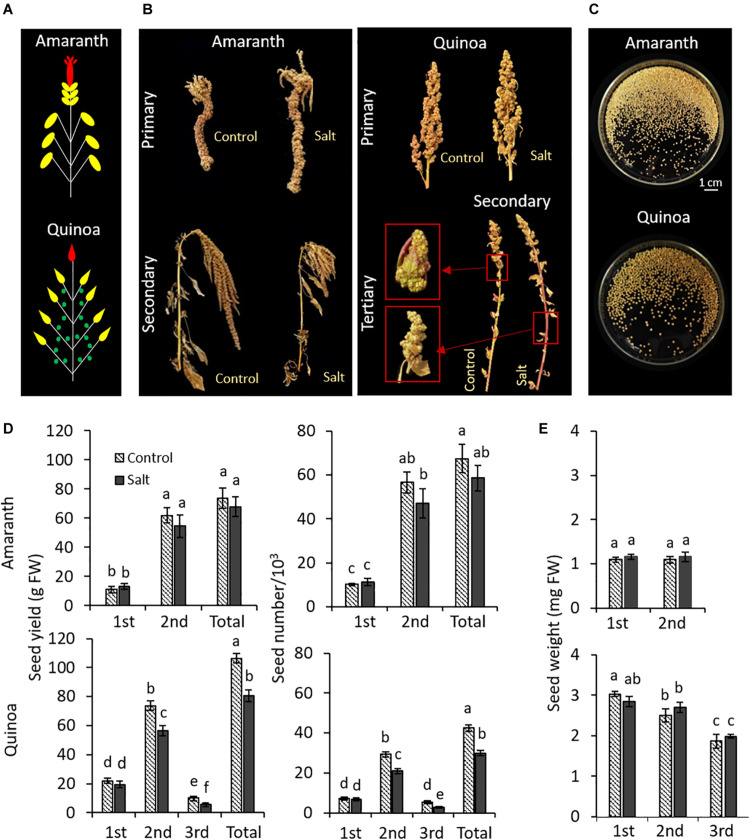
Effect of salinity on seed yields in different panicle types of amaranth and quinoa. Plants were grown in control and salt stress (100 mM NaCl) for 50 days and seeds were harvested when plants were fully dried (15 days after treatments were suppressed). **(A)** Diagram showing the locations of the main panicle (first panicle to emerge) colored in red, secondary panicles at the tip of each branch (second panicle, emerging after the main panicle) colored in yellow, and for quinoa also tertiary panicles from nodes within branches (third panicle, emerging after the second panicle) colored in green. **(B)** Representative images of different panicles of amaranth and quinoa on the day of harvesting, from plants in control condition and plants subjected to salt stress. **(C)** Images of seeds collected for each species. **(D)** Seed yield (g) and seed number produced for each panicle type and in total per plant of amaranth and quinoa, from plants in control condition and plants subjected to salt stress. **(E)** Average seed weight of a seed of each panicle type in amaranth and quinoa, from plants in control condition and plants subjected to salt stress. Values are means ± SE (*n* = 10 plants). Different letters indicate significant differences by Tukey’s test between treatment and panicles in each species (*p* ≤ 0.05).

Amaranth and quinoa plants develop different panicles, as may be observed in the diagram showing the locations of the main and secondary panicles for amaranth and quinoa; tertiary panicles were also observed in this latter species (Figure 8A). Representative images of panicles and seeds from plants in control and salt stress are shown in Figures 8B,C, respectively. The seed yield was not affected by salinity in amaranth, since both total seed grams and number were similar in the control and salt-treated plants (Figures 8D,E). However, in quinoa, the seed yield was significantly reduced in secondary and tertiary panicles (23.4 and 45.4%, respectively), and it is reflected by a reduction of total yield, which was due to a lower number of seeds, and not to a smaller size, which was not affected by salinity (Figures 8D,E).

## Discussion

### Amaranth Exhibits Salt Tolerance Throughout Its Life Cycle

Amaranth and quinoa are regarded as attractive crops that might assist to sustain food security in the current scenario of climate change (Topwal, 2019; Tovar et al., 2020). However, although both species are generally considered as salt-tolerant ones, the degree of this tolerance may vary among species and even among varieties (Kiani-Pouya et al., 2019). For example, plant growth was maintained at salt stress levels between 100 and 200 mM NaCl in quinoa (Hariadi et al., 2011), although in other studies, salinity progressively reduced plant biomass from 100 mM NaCl onward (Cai and Gao, 2020). Within the main grain species of amaranth, the shoot growth of *A. cruentus* was already reduced by 90 mM NaCl concentration (Lavini et al., 2016; Gandonou et al., 2018). In this study, we first selected a salt-tolerant variety for each species, amaranth and quinoa, on the basis of their salt tolerance during seed germination and in the development of young plants (Supplementary Figures 1–4). The selected amaranth variety (Kwicha Peru) belongs to the *A. caudatus* species, and it is an amaranth grain species practically unknown from the point of view of its potential salt tolerance.

It is known that the effects induced by salinity may be different at the vegetative and reproductive plant development stages. When salt tolerance of the two selected varieties of amaranth and quinoa was evaluated at mid-term (100 mM NaCl for 20 days), both showed a similar response as their shoot vegetative biomasses were not affected by salinity (Figure 1A). However, when we evaluated the long-term salt tolerance on the basis of seed yield, the most interesting salt tolerance trait in grain species, only amaranth maintained such tolerance, since grain production was not affected by the saline conditions applied (Figure 8D). The deleterious effect induced by long-term salinity in quinoa was observed in both secondary and tertiary panicles, which reduced their seed yields due to reduced seed number, while the seed size was not affected. Recently, Tovar et al. (2020) observed that heat stress affected quinoa seed yield, with yield losses being the result of lower number of seeds produced per plant, as it is observed in our study. It may be due to the fact that flowering is a developmental stage very susceptible to abiotic stress (Lesjak and Calderini, 2017; Egea et al., 2018), and we applied the salt treatment just when the main panicle began to emerge. In summary, the salt tolerance degree depends on the exposure time to such stress and the developmental stage in quinoa, while amaranth maintains a similar plant growth throughout its life cycle, both at the vegetative and reproductive levels.

### Amaranth Exhibits High Ability to Reduce the Water Loss Through the Leaves Under Salt Stress

Salinity tolerance is achieved through different mechanisms operating simultaneously, but these were differently activated in amaranth and quinoa. The first effect induced by salinity is reduction of water uptake by the root due to osmotic stress. However, the water contents in roots of amaranth and quinoa increased after 20 days of 100 mM NaCl treatment compared to control plants, which indicates that root ability to uptake water under salinity conditions was similar in both species, independent of the different root development of each. However, the differences between amaranth and quinoa came out in the leaves, as only amaranth increased its leaf water content with salinity but not quinoa (Figure 1B). The hydric status of the leaves depends on two processes, the ability to reduce osmotic potential and the ability to avoid the water loss through cuticle and stomata (Albaladejo, 2017). Quinoa exhibited a higher reduction of its leaf osmotic potential with salinity, while amaranth had lower basal levels of stomatal conductance and transpiration rate than quinoa. What is more, both traits experienced a higher reduction by salinity in amaranth compared to quinoa (Figure 1C). These results suggest that maintenance of osmotic homeostasis in salt stress in quinoa is mainly due to its high capacity of osmotic adjustment, so this species has a relatively minor need to reduce leaf water loss. The high osmotic adjustment ability of quinoa under salt stress was already observed by Hariadi et al. (2011), and the authors observed that this was mainly achieved by accumulation of inorganic solutes.

The other mechanism contributing to the high salinity tolerance of halophytes is reduced water loss through the leaves (Flowers and Colmer, 2008), and this mechanism seems to be the predominant one in amaranth. Thus, the stomatal density (SD) was lower in amaranth than in quinoa in leaves from control plants, which was even reduced by salinity in the abaxial surface (Figure 2A). Precisely, reduced SD has been proposed as an important mechanism for reduction of leaf water loss in salt-tolerant halophyte species (Orsini et al., 2011; Shabala et al., 2013). The reason is that cuticular pores in the leaf epidermis are concentrated around the stomata, and therefore, having a few fully opened stomata would be a best suited strategy to prevent water loss than having many partially closed ones because transpiration through cuticular pores or residual transpiration cannot be controlled and may be an important contribution to leaf water loss (Hasanuzzaman et al., 2017). This mechanism is observed in the abaxial surface of amaranth, showing lower SD and higher stomatal aperture (SA) compared with quinoa (Figures 2A,B). In addition to the particular characteristics of amaranth leaf ultrastructure, showing the typical Kranz type leaf anatomy (Ueno, 2001) (Figure 3), anatomical changes could also contribute to reduce the water loss. These could be related with the increase in cell number by reducing the cell size and increasing the density of mesophyll cells (Table 1), which could allow amaranth to attain a greater capacity to store water.

An interesting question is to elucidate whether there were differences between both species in the oxidative stress caused by salinity. Several studies have linked a high antioxidant capacity with a high osmotic stress tolerance in abiotic stress (Jaleel et al., 2009; Farmer and Mueller, 2013). Salinity did not induce any change in MDA content and TEAC values in amaranth, but in quinoa, the MDA content was significantly increased under such stressful condition (Figures 4A,B). With respect to MDA accumulation, it is necessary to keep in mind that MDA may act as a protection mechanism rather than being an indicator of oxidative damage, since MDA can exert a positive role by activating regulatory genes involved in oxidative homeostasis (Morales and Munné-Bosch, 2019). This could be occurring in quinoa leaves where the MDA content was twice higher in salt than in control condition. Both roles could be assigned, but the increase observed in the ratio MDA/TEAC in salinity suggests that quinoa is more sensitive to oxidative stress than amaranth under saline conditions (Figure 4C).

### The Na^+^ Excluder Character in Amaranth Is Associated to Constitutive High Basal Levels of Genes Involved in Na^+^ and K^+^ Homeostasis

In this study, it has been demonstrated that strategies used by amaranth and quinoa to maintain Na^+^ homeostasis under salinity are very different. Amaranth exhibited higher Na^+^ accumulation in the root followed by the stem and a much lower accumulation in the leaf, with this pattern similar enough to that shown by glycophytic or Na^+^ excluder species (Egea et al., 2018). Contrarily, quinoa exhibited a halophytic or Na^+^ includer behavior, where the highest Na^+^ accumulation was found in the shoot (Figure 5A). The includer behavior of quinoa is mainly due to the presence of EBCs in its leaves, which are able to accumulate very high amounts of Na^+^. In fact, Kiani-Pouya et al. (2020) found that different accessions of quinoa with a low number of EBCs used the strategy of Na^+^ exclusion at the root level, maintaining lower Na^+^ concentration in their leaves upon salinity exposure, similar to that observed in amaranth, compared with accessions with a high number of EBCs.

It has also been claimed that salt tolerance mechanisms are not really different between halophytes and glycophytes, but that halophytes may be best prepared against the stress and make a more efficient use of common mechanisms (Flowers et al., 2010; Volkov, 2015). In fact, the different expression patterns of main genes involved in Na^+^ homeostasis, both at basal level or changes in levels induced by salinity, supports the glycophytic or excluder behavior of amaranth, contrary to the halophytic or includer behavior of quinoa. Up to our knowledge, the expression levels of genes involved in Na^+^ and K^+^ homeostasis has not been analyzed in amaranth, and therefore, these would be the first results published. Regarding Na^+^ transporters, we have identified an amaranth ortholog of *CqHKT1;1*, which was named *AhHKT1;1*, and this was preferentially expressed in the root. The expression of this gene was analyzed together with *AhSOS1* and *AhNHX1*, as well as *HAK5* and *SKOR*, involved in K^+^ homeostasis. Interestingly, we observed a constitutively higher expression of these main genes involved in Na^+^ and K^+^ homeostasis in roots of amaranth than in quinoa (Figure 6A and Supplementary Figure 11A). In this regard, the different basal expressions of the main genes involved in stress tolerance have been proposed as a key point in the assessment of salt tolerance of halophytes. Thus, the halotolerant wild tomato species *S. pennellii* showed important constitutive gene expression differences with respect to cultivated tomato (Albaladejo, 2017). Another example is the salt-tolerant halophyte *Salicornia dolichostachya*, which exhibited constitutively high levels of *SOS1* expression in roots compared with its glycophyte relative *S. oleracea* (Katschnig et al., 2015). In summary, the different salt-tolerance mechanisms displayed by amaranth and quinoa could be due, at least partially, to changes in constitutive basal levels of genes involved in Na^+^ and K^+^ homeostasis.

According to the very high Na^+^ accumulation induced by salinity in amaranth root, *AhSOS1* was significantly reduced by salinity, indicating a lower Na^+^ extrusion out of the root (Figure 6B). Regarding *AhHKT1;1*, although an increase in the expression in the root induced by salinity would have been expected in order to reduce the Na^+^ loading into the xylem, the opposite response was observed (Figure 6C). However, despite this reduction, the highest expression level of *HKT1;1* was found in the root, both under the control and salt stress conditions, and this was also higher than in the quinoa root (Figure 6C), which could also be responsible for the greater retention of Na^+^ in the root in amaranth with respect to quinoa (Figure 5A). On the other hand, the possibility remains that the identified amaranth ortholog of *HKT1;1* could exert different roles than *CqHKT1;1* in quinoa, as it was recently observed in the barley transporter *HvHKT1;5*, which transported Na^+^ in the opposite direction to the orthologs in other cereal crops, that is, from the root to the xylem (Huang et al., 2020). Although future studies will solve this question, it cannot be discarded as a distinctive function in Na^+^ transport for the homolog of *HKT1;1* identified in amaranth. In quinoa, the highest basal *HKT1;1* expression was observed in the stem, in agreement with the highest Na^+^ accumulation found in that plant organ (Figures 5A, 6A). Meanwhile, *SOS1* expression was upregulated in both the shoot organs (leaf and stem), and either the HKT1-type gene in each different shoot organ analyzed: *CqHKT1;1* was induced by salinity in the stem and *CqHKT1;2* in the leaf (Figures 6B–D). It is not easy to explain the increased amounts of transcripts of both *CqSOS1* and *CqHKT1s* genes in quinoa shoot under salinity, since these two transporters seem to function antagonistically, and a futile cycle of Na^+^ loading and unloading might be occurring. Nevertheless, a possible explanation could be that *SOS1* is involved in fine-tuning the regulation of Na^+^ cytoplasmic concentration in quinoa shoot cells, as it is known that a too high concentration of Na^+^ in the cytosol is equally detrimental for both glycophytes and halophytes, and it must be avoided (Flowers and Colmer, 2008).

One trait correlated with salt tolerance is the Na^+^/K^+^ ratio, which has been not only confirmed in glycophytic crop species (Cuartero et al., 2006) but also in halophytes (Cai and Gao, 2020). In our study, both amaranth and quinoa roots decreased its K^+^ content under saline conditions, while the leaf of amaranth showed not only the lowest Na^+^ accumulation but also increased K^+^ content under salinity, which resulted in low Na^+^/K^+^ ratio in the leaf (Figure 5). When the expression level of *HAK5* (involved in high-affinity K^+^ uptake) was analyzed, we observed a marked decrease under saline conditions in roots in both species, as it occurred in *Arabidopsis* with *AtHAK5* in salt stress (Nieves-Cordones et al., 2010) (Supplementary Figure 11B). These results would explain the observed K^+^ decrease in amaranth and quinoa roots, as *HAK5* is involved in K^+^ uptake at the root level (Nieves-Cordones et al., 2010). Salinity also induced in amaranth a decrease in expression level of the *SKOR* gene, involved in the long-distance K^+^ transport from the root to the shoot through the xylem vessels (Nieves-Cordones et al., 2016). However, despite the decrease induced by salinity in the expression, the basal levels of *HAK5* and *SKOR* expressions in roots were much higher in amaranth than in quinoa, which could result in a more efficient maintenance of the K^+^ homeostasis in amaranth compared to that in quinoa (Supplementary Figure 11A). Taking together the results from the Na^+^ and K^+^ distribution and the expression patterns of the main genes involved in the homeostasis of these ions, it is possible to assert that amaranth uses a glycophytic strategy (Na^+^ excluder strategy) to confront salt stress.

In conclusion, the main strategy of amaranth to maintain osmotic homeostasis is based on its high ability to reduce water loss through the leaves under salt stress. On the other hand, amaranth displayed a higher root/leaf water content ratio than quinoa, both at the control and under salt stress, which suggests that amaranth presents a higher ability than quinoa to harbor Na^+^ ions in a non-toxic way in the roots. Finally, the high constitutive expression levels of genes involved in Na^+^ and K^+^ homeostasis could be a key point in the salt tolerance displayed by amaranth. These results constitute an important advance in the knowledge of the tolerance mechanisms operating in a promising species such as amaranth, able to maintain seed yield under salinity, and they point out the importance of considering simultaneously the anatomical, physiological, and molecular changes caused by salinity in order to develop efficient strategies to increase salt tolerance.

## Data Availability Statement

The original contributions generated for this study are included in the article/Supplementary Material, further inquiries can be directed to the corresponding author.

## Author Contributions

YE, AF-O, BM, FF, and IE performed the experiments. MB and IE contributed in the conceptualization of the study and design of the experiments. IE, MB, and JE-F conceived the project and research, and supervised the experiments. YE, FF, MB, and IE contributed to the analysis and interpretation of the results. YE, MB, and IE wrote the manuscript with contributions from all the authors. All authors have critically reviewed the manuscript.

## Conflict of Interest

The authors declare that the research was conducted in the absence of any commercial or financial relationships that could be construed as a potential conflict of interest.

## Footnotes

1 http://imagej.nih.gov/ij/index.html

2 https://phytozome.jgi.doe.gov/pz/portal.html

3 https://bioinformatics.psb.ugent.be/plaza/versions/plaza_v4_dicots/

4 http://ClustalW.ddbj.nig.ac.jp/

## Supplementary Material

Supplementary Figure 1 Seed germination percentage of different amaranth varieties at 0 (control), 50, 100, and 150 mM NaCl. B, Blanco; K1, Kwicha Perú; OB, Oscar Blanco; K2, Kwicha Granada, and Bur, Burganda amaranth varieties. Values are means ± SE of three plates of 50 seeds per salt level. Asterisks indicate significant differences between mean values of increasing salt levels with respect to control for each amaranth variety (Student’s *t*-test, *p* < 0.05).

Supplementary Figure 2 Salt responses of different amaranth varieties. Plants with two true leaves were treated with increasing salt levels: results before salt treatment (0 day), and after 200 mM NaCl for 5 days (5 days) plus 300 mM NaCl for 6 days (a total of 11 days of salt treatment, DST). **(A)** Representative phenotypes of amaranth plants before salt treatment and after 11 DST. **(B)** Shoot biomass and **(C)** chlorophyll content of plants after 0, 5, and 11 DST. B, Blanco; K1, Kwicha Perú; OB, Oscar Blanco; K2, Kwicha Granada, and Bur, Burganda amaranth varieties. Values are means ± SE (*n* = 9 plants). Asterisks indicate significant differences between mean values of increasing salt levels with respect to control for each amaranth variety (Student’s *t*-test, *p* < 0.05).

Supplementary Figure 3 Water loss rate of different amaranth varieties measured in detached 1st leaf of plants grown in control condition. B, Blanco; K1, Kwicha Perú; OB, Oscar Blanco; K2, Kwicha Granada, and Bur, Burganda amaranth varieties. Measures were taken during the first 6 h and after 24 h.

Supplementary Figure 4 Salt response of four quinoa varieties, QQ74 (QQ), Cherry Vainilla (CV), Inca (IN) and Cahuil (CA). **(A)** Representative images of the four quinoa varieties grown in pots 50 days after germination. **(B)** Seed germination percentage in control and 100 mM NaCl. Values are means ± SE of three plates of 50 seeds each per salt level. Asterisks indicate significant differences between mean values of salt with respect to control (Student’s *t*-test, *p* < 0.05). **(C)** Relative water loss of leaves for each variety of salt-treated plants with respect to control ones, after 1 and 2 days of salt treatment (100 mM NaCl). Values are means ± SE (*n* = 9 plants). Different letters indicate significant differences among quinoa varieties (LSD, *p* < 0.05).

Supplementary Figure 5 The phylogenic relationship between *Amaranthus hypochondriacus* and *Chenopodium quinoa* **(A)** SOS1, **(B)** HKTs, **(C)** NHX1, **(D)** HAK5, and **(E)** SKOR with proteins from other species of the Amaranthaceae family, *Arabidopsis thaliana* and *Oryza sativa*. The phylogenic trees were constructed using ClustalW method from the DNASTAR software package (RAxML bootstrap). Protein accession numbers and species names used for construction of the phylogenetic trees are showed in Supplementary Table 1.

Supplementary Figure 6 Alignment of SOS1 protein sequences. The multiple sequence alignment of SOS1 protein sequences was generated with ClustalW (http://www.ebi.ac.uk/Tools/msa/ClustalW2/). Identical amino acids are marked with an asterisk (^∗^) below. Protein accession numbers and species names used for amino acid sequences alignment are showed in Supplementary Table 1.

Supplementary Figure 7 Alignment of HKTs protein sequences. The multiple sequence alignment of HKTs protein sequences was generated with ClustalW (http://www.ebi.ac.uk/Tools/msa/ClustalW2/). Identical amino acids are marked with an asterisk (^∗^) below. Protein accession numbers and species names used for amino acid sequences alignment are showed in Supplementary Table 1.

Supplementary Figure 8 Alignment of NHXs protein sequences. The multiple sequence alignment of NHXs protein sequences was generated with ClustalW (http://www.ebi.ac.uk/Tools/msa/ClustalW2/). Identical amino acids are marked with an asterisk (^∗^) below. Protein accession numbers and species names used for amino acid sequences alignment are showed in Supplementary Table 1.

Supplementary Figure 9 Alignment of HAK protein sequences. The multiple sequence alignment of HAK protein sequences was generated with ClustalW (http://www.ebi.ac.uk/Tools/msa/ClustalW2/). Identical amino acids are marked with an asterisk (^∗^) below. Protein accession numbers and species names used for amino acid sequences alignment are showed in Supplementary Table 1.

Supplementary Figure 10 Alignment of SKOR protein sequences. The multiple sequence alignment of SKOR protein sequences was generated with ClustalW (http://www.ebi.ac.uk/Tools/msa/ClustalW2/). Identical amino acids are marked with an asterisk (^∗^) below. Protein accession numbers and species names used for amino acid sequences alignment are showed in Supplementary Table 1.

Supplementary Figure 11 Constitutive and relative expression of genes involved in K^+^ homeostasis in amaranth and quinoa. **(A)** Comparative constitutive expression of *HAK5* and *SKOR* genes analyzed in root, stem and leaf of plants grown in control condition. **(B)** Changes in relative expression levels of genes involved in K^+^ homeostasis (*HAK5* and *SKOR*) in root of amaranth and quinoa plants grown in control and salt stress (100 mM NaCl) for 20 days. The expression of root in control was set to 1 for amaranth. Values are means ± SE of three biological replicates (each one of 3 plants, *n* = 9). Different letters indicate significant differences among mean values of constitutive expression levels of *HAK5* and *SKOR* genes **(A)** (LSD test, *p* < 0.05). Asterisks indicate significant differences between control and salt treatments in **(B)** (Student’s *t*-test, *p* < 0.05).

Supplementary Table 1 Protein accession numbers and species of genes used in phylogenetic relationships, alignments and design of the degenerate primers.

Supplementary Table 2 Sequences of primers for RT-qPCR gene expression analysis used in this study.
