# Supplementary material for: Unraveling the Strategies Used by the Underexploited Amaranth Species to Confront Salt Stress: Similarities and Differences With Quinoa Species
Source: Front Plant Sci. 2021 Feb 10;12:604481. doi: 10.3389/fpls.2021.604481 (PMC7902779; doi:10.3389/fpls.2021.604481)
Supplement: Supplementary Figure 1 — Seed germination percentage of different amaranth varieties at 0 (control), 50, 100, and 150 mM NaCl. B, Blanco; K1, Kwicha Perú; OB, Oscar Blanco; K2, Kwicha Granada, and Bur, Burganda amaranth varieties. Values are means ± SE of three plates of 50 seeds per salt level. Asterisks indicate significant differences between mean values of increasing salt levels with respect to control for each amaranth variety (Student’s t-test, p < 0.05). [file Data_Sheet_1.pdf]

Supplementary Material

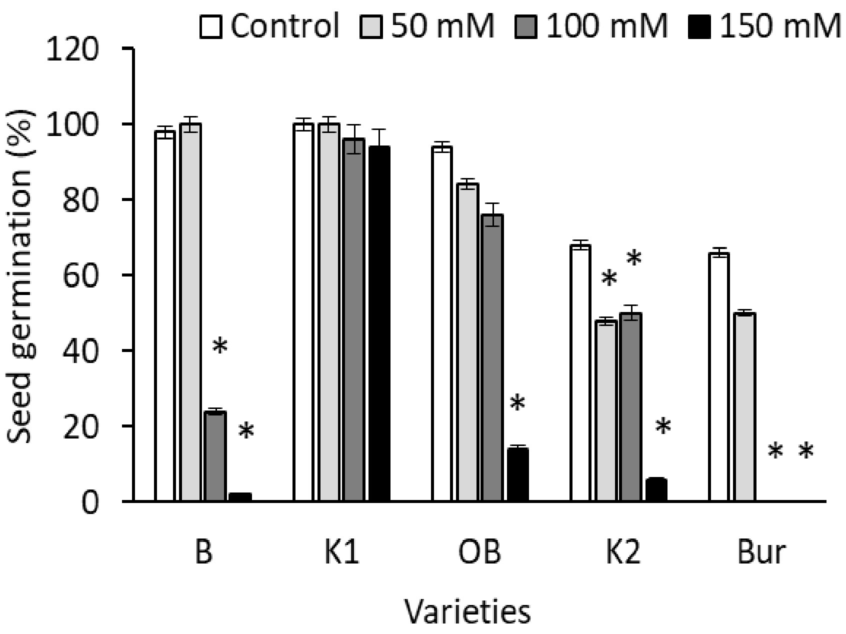

Supplementary Figure S1. Seed germination percentage of different amaranth varieties at 0 (control), 50, 100 and 150 mM NaCl. B: Blanco; K1: Kwicha Perú; OB: Oscar Blanco; K2: Kwicha Granada, and Bur: Burganda amaranth varieties. Values are means ± SE of three plates of 50 seeds per salt level. Asterisks indicate significant differences between mean values of increasing salt levels with respect to control for each amaranth variety (Student's *t*-test, $p \leq 0.05$).

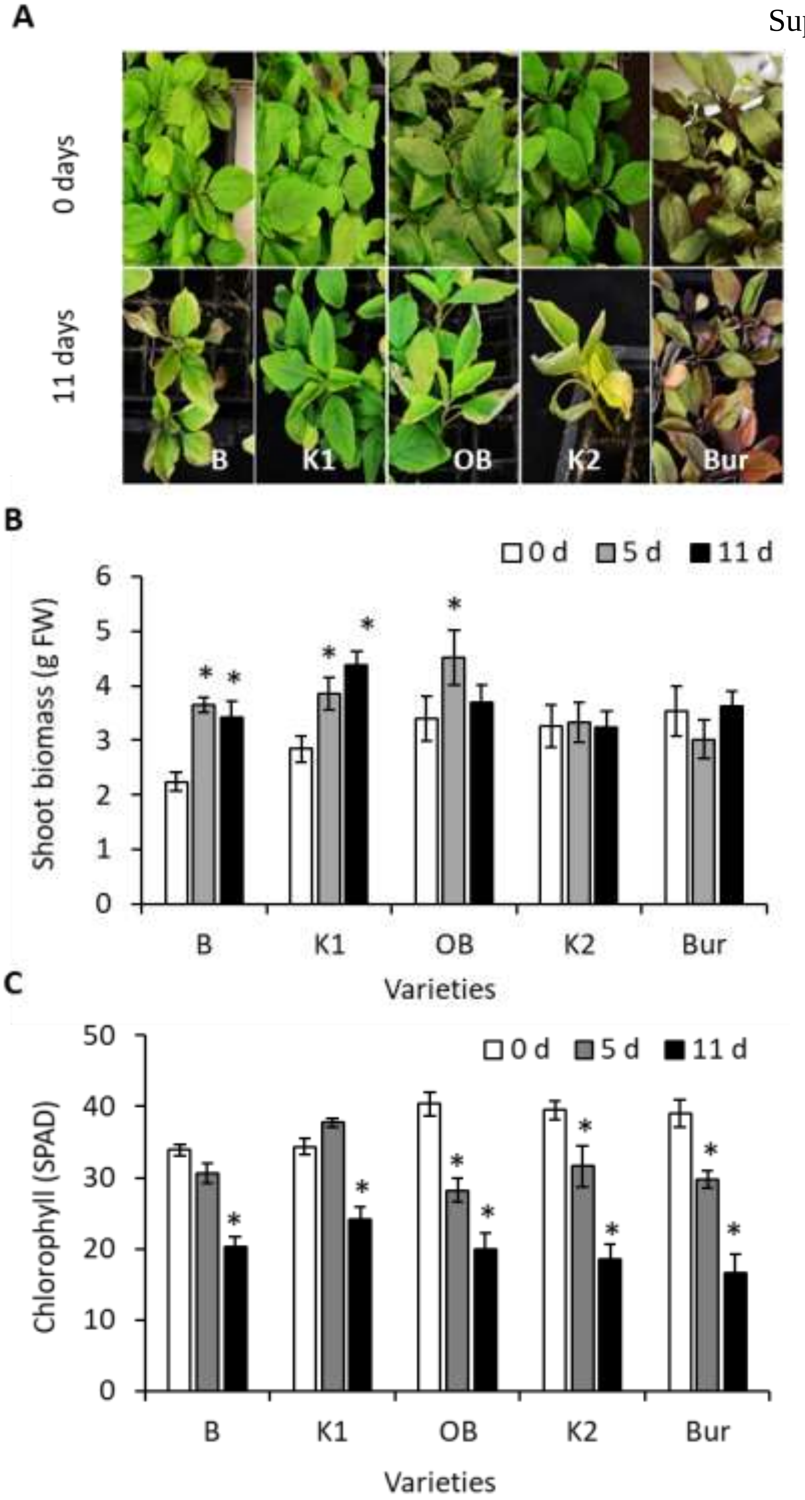

Supplementary Figure S2. Salt responses of different amaranth varieties. Plants with two true leaves were treated with increasing salt levels: results before salt treatment (0d), 200 mM NaCl for 5 days (5d) plus 300 mM NaCl for 6 days (a total of 11 days of salt treatment, DST). (A) Representative phenotypes of amaranth plants before salt treatment and after 11 DST. (B) Shoot biomass and (C) chlorophyll content of plants after 0, 5 and 11 DST. B: Blanco; K1: Kwicha Perú; OB: Oscar Blanco; K2: Kwicha Granada, and Bur: Burganda amaranth varieties. Values are means ± SE (n = 9 plants). Asterisks indicate significant differences between mean values of increasing salt levels with respect to control for each amaranth variety (Student's *t*-test, $p \leq 0.05$).

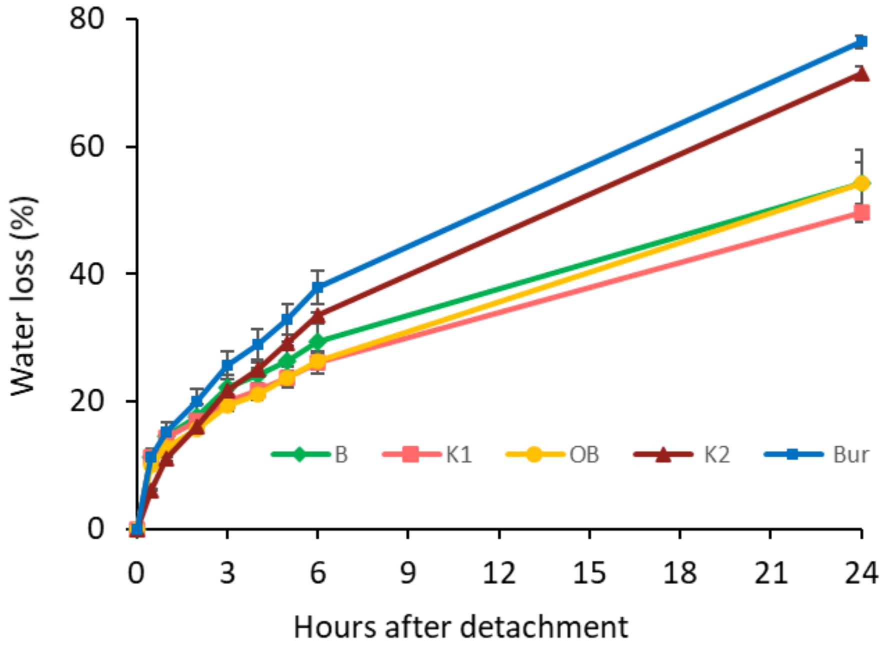

Supplementary Figure S3. Water loss rate of different amaranth varieties measured in detached 1st leaf of plants grown in control condition. B: Blanco; K1: Kwicha Perú; OB: Oscar Blanco; K2: Kwicha Granada, and Bur: Burganda amaranth varieties. Measures were taken during the first 6 h and after 24 h.

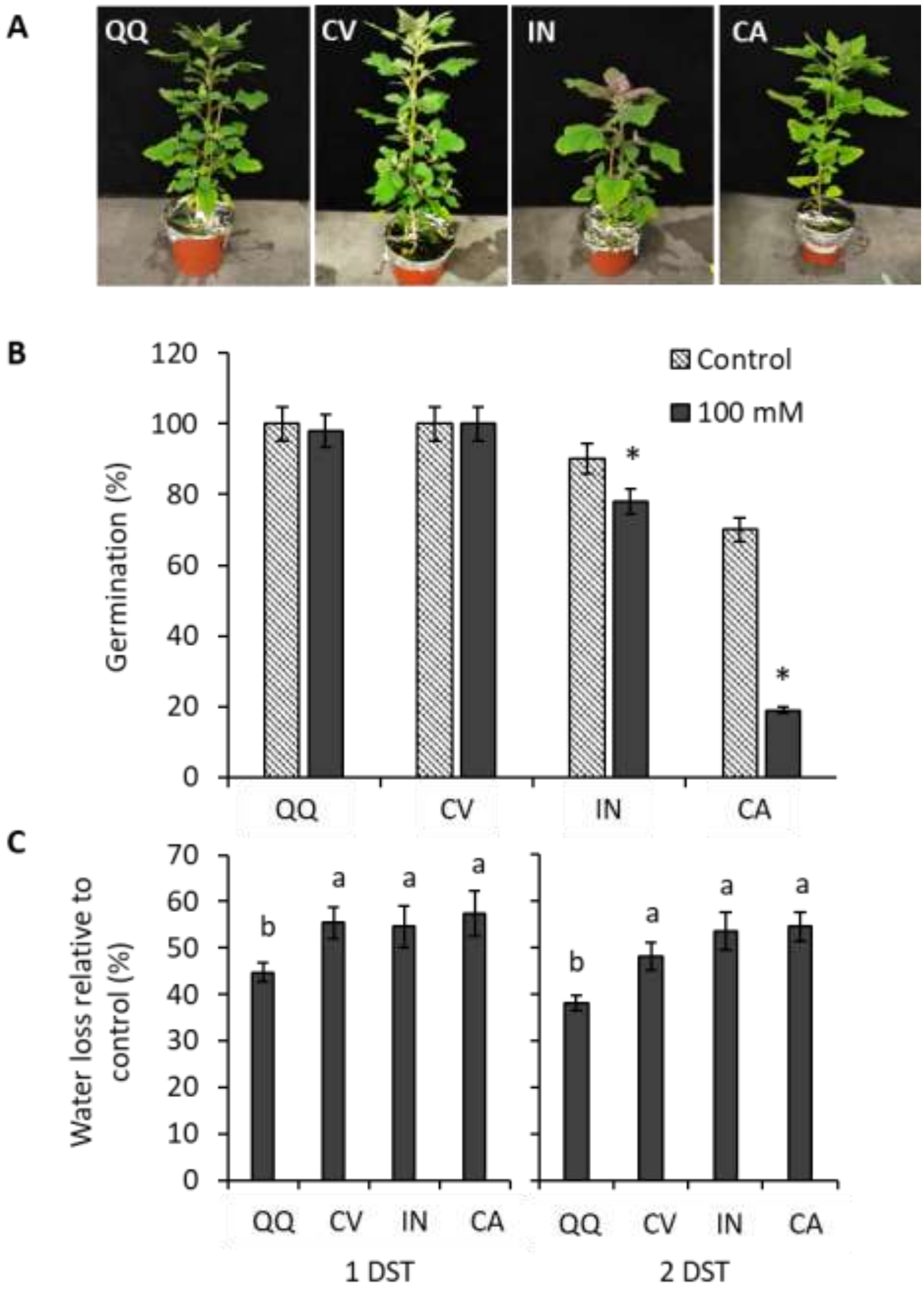

Supplementary Figure S4. Salt response of four quinoa varieties, QQ74 (QQ), Cherry vainilla (CV), Inca (IN) and Cahuil (CA). (A) Representative images of the four quinoa varieties grown in pots 50 days after germination. (B) Seed germination percentage in control and 100 mM NaCl. Values are means ± SE of three plates of 50 seeds each per salt level. Asterisks indicate significant differences between mean values of salt with respect to control (Student's *t*-test, $p \leq 0.05$). (C) Relative water loss of leaves for each variety of salt-treated plants with respect to control ones, after 1 and 2 days of salt treatment (100 mM NaCl). Values are means ± SE (n = 9 plants). Different letters indicate significant differences among quinoa varieties (LSD, $p \leq 0.05$).

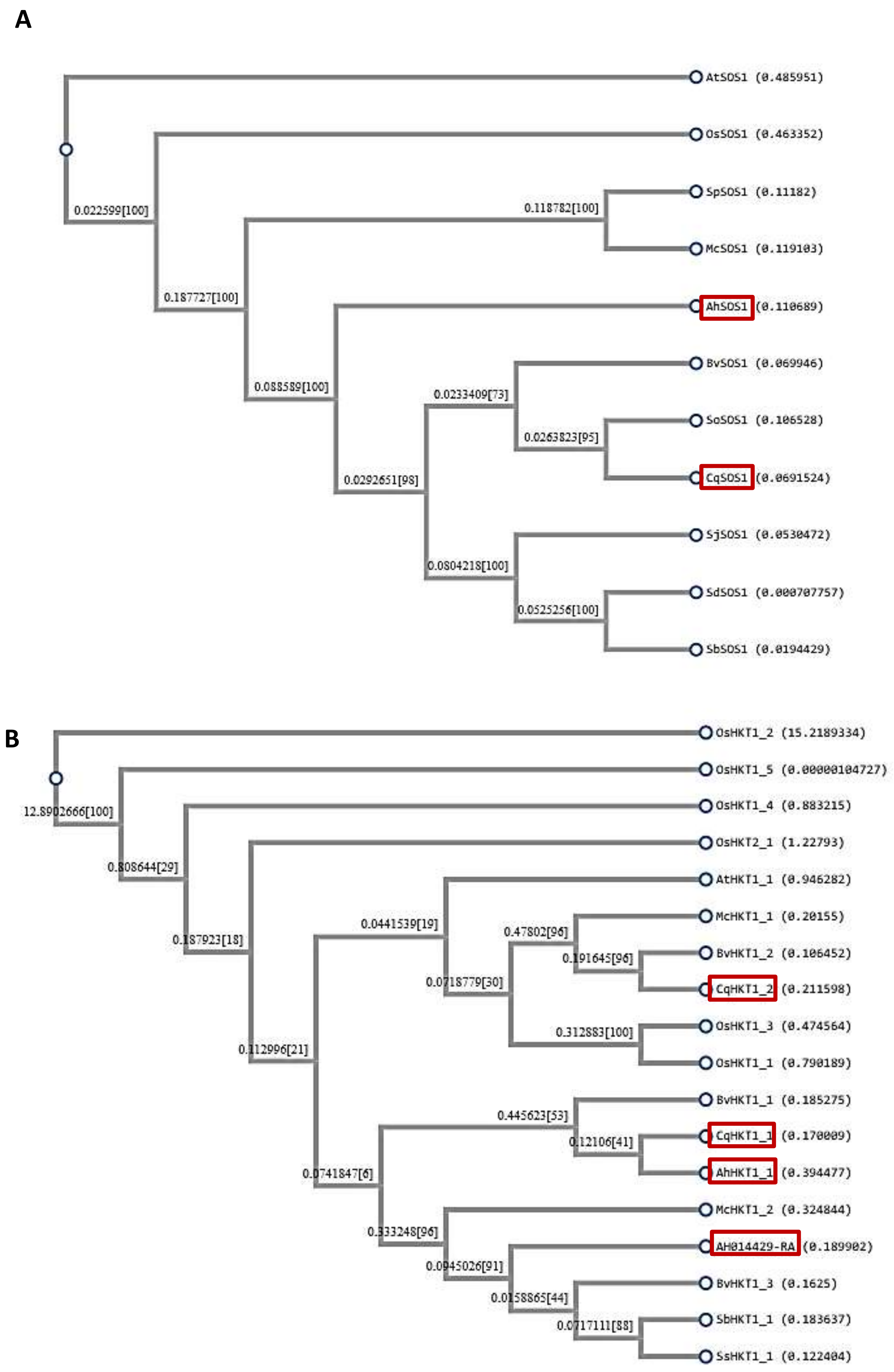
A
AtSOS1 (0.485951)
OsSOS1 (0.463352)
SpSOS1 (0.11182)
McSOS1 (0.119103)
AhSOS1 (0.110689)
BvSOS1 (0.069946)
SoSOS1 (0.106528)
CqSOS1 (0.0691524)
SjSOS1 (0.0530472)
SdSOS1 (0.000707757)
SbSOS1 (0.0194429)
0.022599[100]
0.187727[100]
0.118782[100]
0.088589[100]
0.0233409[73]
0.0263823[95]
0.0292651[98]
0.0804218[100]
0.0525256[100]
B
OsHKT1_2 (15.2189334)
OsHKT1_5 (0.00000104727)
OsHKT1_4 (0.883215)
OsHKT2_1 (1.22793)
AtHKT1_1 (0.946282)
McHKT1_1 (0.20155)
BvHKT1_2 (0.106452)
CqHKT1_2 (0.211598)
OsHKT1_3 (0.474564)
OsHKT1_1 (0.790189)
BvHKT1_1 (0.185275)
CqHKT1_1 (0.170009)
AhHKT1_1 (0.394477)
McHKT1_2 (0.324844)
AH014429-RA (0.189902)
BvHKT1_3 (0.1625)
SbHKT1_1 (0.183637)
SsHKT1_1 (0.122404)
12.8902666[100]
0.808644[29]
0.187923[18]
0.112996[21]
0.0441539[19]
0.47802[96]
0.191645[96]
0.0718779[30]
0.312883[100]
0.445623[53]
0.12106[41]
0.0741847[6]
0.333248[96]
0.0945026[91]
0.0158865[44]
0.0717111[88]

C

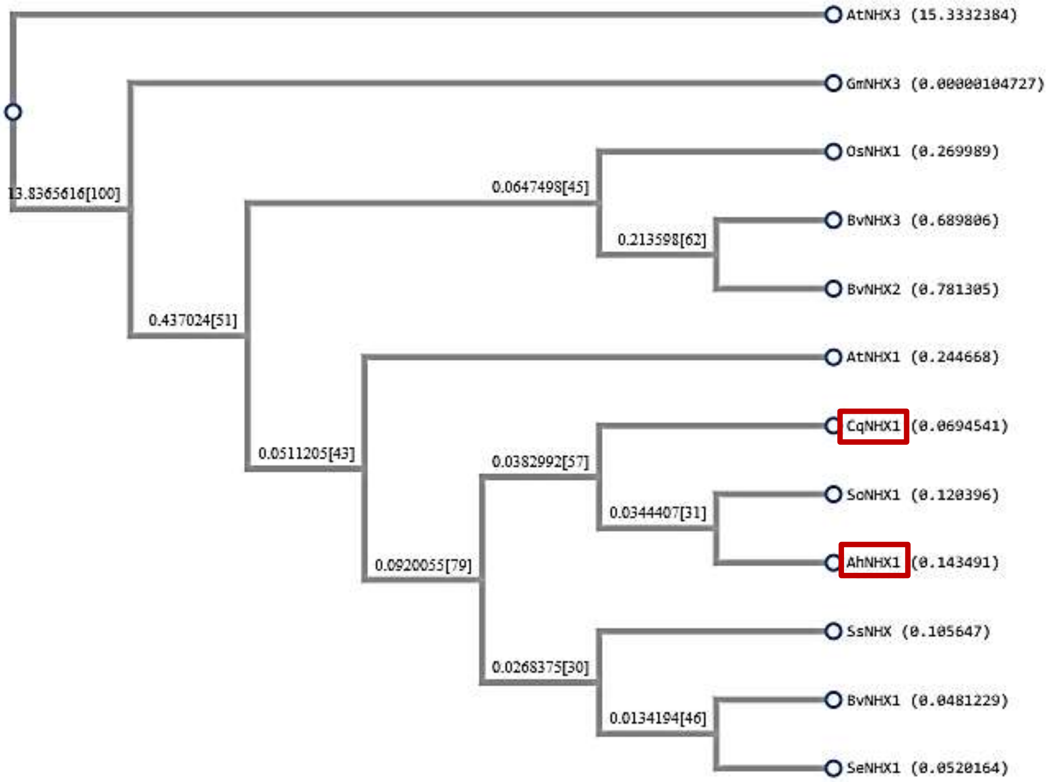
AtNHX3 (15.3332384)
GmNHX3 (0.00000104727)
OsNHX1 (0.269989)
BvNHX3 (0.689806)
BvNHX2 (0.781305)
AtNHX1 (0.244668)
CqNHX1 (0.0694541)
SoNHX1 (0.120396)
AhNHX1 (0.143491)
SsNHX (0.105647)
BvNHX1 (0.0481229)
SeNHX1 (0.0520164)
13.8365616[100]
0.437024[51]
0.0647498[45]
0.213598[62]
0.0511205[43]
0.0382992[57]
0.0344407[31]
0.0920055[79]
0.0268375[30]
0.0134194[46]

D

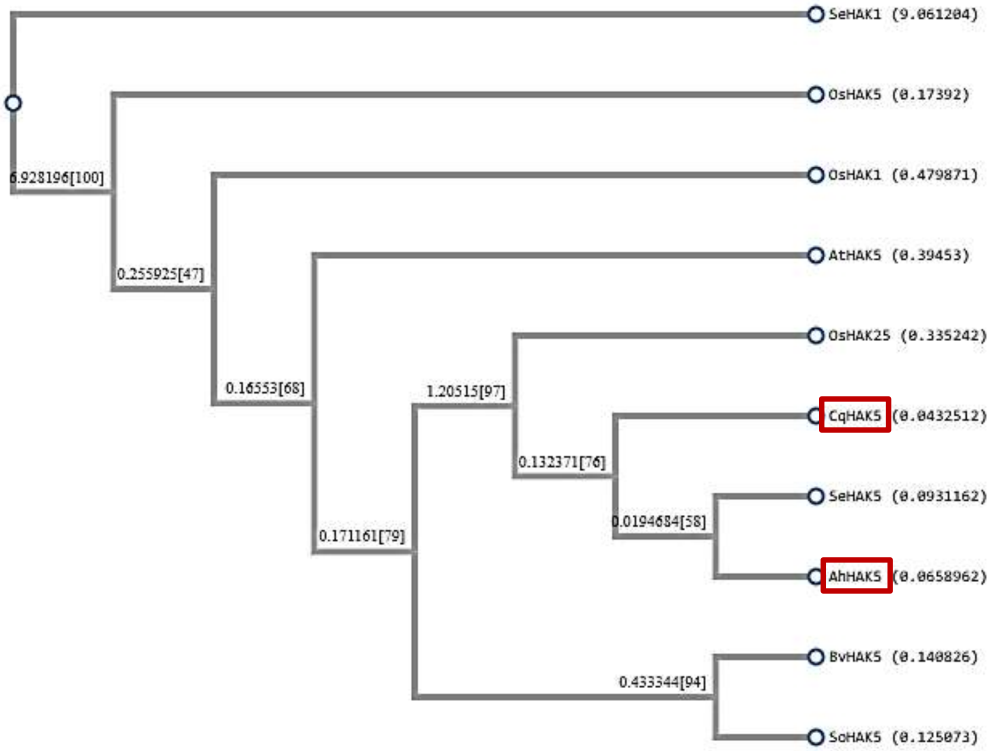
SeHAK1 (9.061204)
OsHAK5 (0.17392)
OsHAK1 (0.479871)
AtHAK5 (0.39453)
OsHAK25 (0.335242)
CqHAK5 (0.0432512)
SeHAK5 (0.0931162)
AhHAK5 (0.0658962)
BvHAK5 (0.140826)
SoHAK5 (0.125073)
6.928196[100]
0.255925[47]
0.16553[68]
1.20515[97]
0.132371[76]
0.0194684[58]
0.171161[79]
0.433344[94]

E

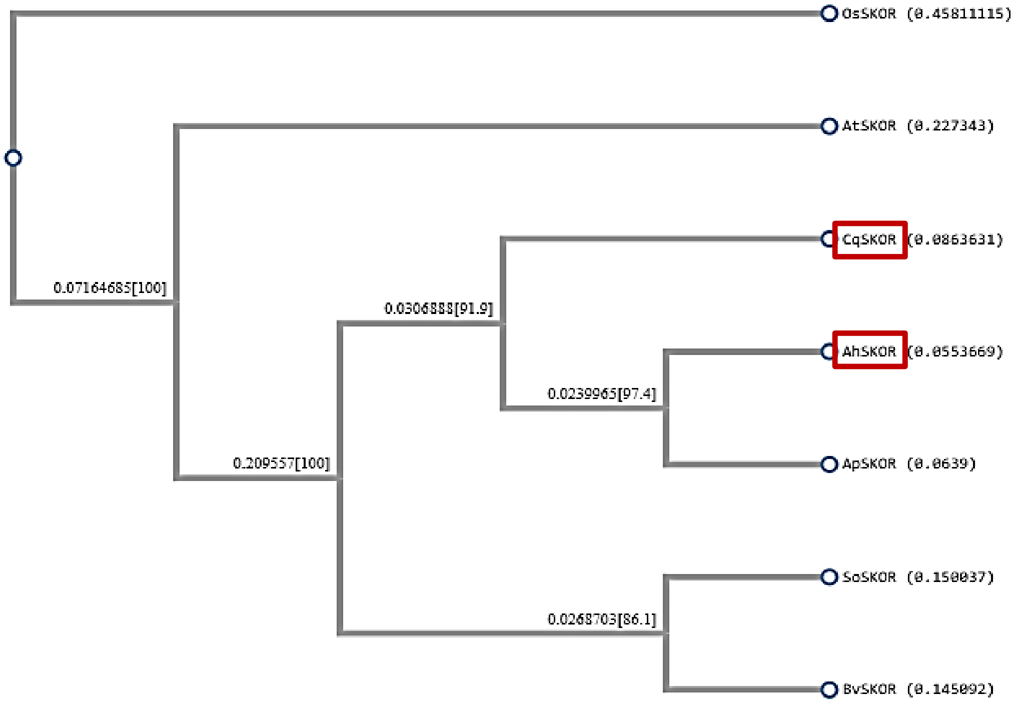

Supplementary figure S5. The phylogenic relationship between *Amaranthus hypocondriacus* and *Chenopodium quinoa* (A) SOS1, (B) HKTs, (C) NHX1, (D) HAK5 and (E) SKOR with proteins from other species of the Amaranthaceae family, *Arabidopsis thaliana* and *Oryza sativa*. The phylogenic trees were constructed using ClustalW method from the DNASTAR software package (RAxML bootstrap). Protein accession numbers and species names used for construction of the phylogenetic trees are showed on Supplementary Table S1.

```
McSOS1          -----------MAALTDFQLPLRLAEEVAANATSAAAAVAAESETNPTDAVIFVGVSLVL
SpSOS1          -----------MAALTDLPFPFRLTELEST-SNSTSTVVAEESSSNPTDAVIFVGVSLVL
SbSOS1          MAASRIEVPFPFILAEDVTAPTT--------SNSTSSSSVMEEESNPSDAVIFFGVSLIL
SdSOS1          MAASRIEVPFPFILAEDVTAPTT--------SNSTSSSSVMEEESNPSDAVIFFGVSLIL
SjSOS1          MAASRIEVPFPFMLAEDVTSPSS--------SNSTVS-AVLEEESNPTDAVIFFGASLIL
CqSOS1          MAAYRIAVPFPLMLAEVGAETT---APMN--STISASAMAEESESNPTDAVIFFGVSLIL
SoSOS1          MAAYRIAVPFPLMLAEVEPASS---AATTSMNTTSAAALAEESASNPSDAVIFFGVSLVL
BvSOS1          MAAYRIAVPLPFMLAEAETVTTNTTN-------SAVVAALVEEESNPTDAVIFFGVSLIL
AhSOS1          --------------------------------------MEESSSNPTDAVIFFGVSLVL
OsSOS1          --------------------------------------MDNPEAEPDDAVLFVGVSLVL
AtSOS1          --------------MTTVIDATMAYRFLEEATDSSSSSSSSKLESSPVDAVLFVGMSLVL
                                                         :  :.* ***:*.* **:*

McSOS1          GIACRHFLRGTKVPYTVALLIIGIGLGSLEYGTKHGLGRFGDGIRLWEHIDPELLLAVFL
SpSOS1          GIACRHFLRGTRVPYTVALLIIGIGLGSLEYGTKHGLGKFGNGIRLWEHIDPDLLLAVFL
SbSOS1          GIACRHFLRGTRVPYTVALLLIGIGLGSLEYGTKHGAGRFGDGIRIWENIDPELLLAVFL
SdSOS1          GIACRHFLRGTRVPYTVALLLIGIGLGSLEYGTKHGAGRFGDGIRIWENIDPELLLAVFL
SjSOS1          GIACRHFLRGTRVPYTVALLVIGIGLGSLEYGTKHGVGRFGDGIRIWENIDPELLLAVFL
CqSOS1          GIACRHFLRGTRVPYTVALLIIGIGLGSLEYGTKHGLGRFGDGIRIWENIDPELLLAVFL
SoSOS1          GILR-----GTRVPYTVALLIIGIGLGSLEYGTKHGVGRFGDGIRIWENIDPELLLAVFL
BvSOS1          GIACRHFLRGTRIPYTVALLVIGIGLGSLEYGTKHGVGRFGDGIRIWENIDPDLLLAVFL
AhSOS1          GIACRHFLRGTRVPYTVALLIIGIGLGSLEYGTKHGAGRFGDGIRIWESINPELLLAVFL
OsSOS1          GIASRHLLRGTRVPYTVALLVLGVALGSLEFGTKHGMGKLGAGIRIWANINPDLLLAVFL
AtSOS1          GIASRHLLRGTRVPYTVALLVIGIALGSLEYGAKHNLGKIGHGIRIWNEIDPELLLAVFL
                **       **::*******::*:.*****:*:**. *::* ***:*  *:*:*******

McSOS1          PALLFESSFSMEVHQIKRCIIQMLILAGPGVLISTFCLGSALKLSFPYDWNWKTSLLLGG
SpSOS1          PALLFESSFSMEIHQIKRCIVQMFLLAGPGVLISTFCLGAALKYSFPYDWNWKTSLLLGG
SbSOS1          PALLFESSFSMEIHQIKRCIAQMVLLAGPRVLISTFCIGVALKLSFPYDWNWTTSLLLGG
SdSOS1          PALLFESSFSMEIHQIKRCIAQMVLLAGPGVLISTFCIGVALKLSFPYDWNWTTSLLLGG
SjSOS1          PALLFESSFSMEIHQIKRCIAQMVLLAGPGVLLSTCILGAALKLTFPYGWNWKTSLLLGG
CqSOS1          PALLFESSFSMEIHQIKRCAAQMILLAGPGVLISTFCLGAALKLSFPYDWSWKTSLLLGG
SoSOS1          PALLFESSFSMEIHQIKRCAAQMVILAGPGVLISTFCLGAALKFSFPYDWNWKTSLLLGG
BvSOS1          PALLFESSFSMEVHQIKRCIAQMVLLAGPGVLISTFALGAALKLSFPYDWNWKTSLLLGG
AhSOS1          PALLFESSFSMEIHQIKRCMAQMVLLAGPGVLISTFCLGTALKLSFPYDWNWKTSLLLGG
OsSOS1          PALLFESSFSMEIHQIKKCMAQMVLLAGPGVLISTFFLGSALKLTFPYNWNWKTSLLLGG
AtSOS1          PALLFESSFSMEVHQIKRCLGQMVLLAVPGVLISTACLGSLVKVTFPYEWDWKTSLLLGG
                ************:****:*  **.:** * **:**  :*  :* :*** *.*.*******

McSOS1          LLSATDPVAVVALLKELGASKKLSTIIEGESLMNDGTAIVVYQLFFQMVLGRSFNVAQII
SpSOS1          LLSATDPVAVVALLKELGASKKLSTIIEGESLMNDGTAIVVYTLFYQMVFGRSFNWGEIV
SbSOS1          LLSATDPVAVVALLKELGASKKLNTIIEGESLMNDGIAIVVYHLFYQMVLGRSFTWAAIL
SdSOS1          LLSATDPVAVVALLKELGASKKLSTIIEGESLMNDGIAIVVYQLFYQMXLGRSFTWVAIL
SjSOS1          LLSATDPVAVVALLKELGASKKLSTIIEGESLMNDGTAIVVYQLFYQMVLGRSFTWVAIV
CqSOS1          LLSATDPVAVVALLKELGASKKLSTIIEGESLMNDGTAIVVYQLFLKMILGRTFNWASIL
SoSOS1          LLSATXPVAVVALLKELGASKKLSTIIEGESLMNDGTAIVVYQLFYQMVLGRSFNWASIL
BvSOS1          LLSATDPVAVVALLKELGASKKLSTIIEGESLMNDGTAIVVYQLFFQMVLGRTFNWASIV
AhSOS1          LLSATDPVAVVALLKELGASKKLSTIIEGESLMNDGTAIVVYQLFFQMALGRSFSWAAIL
OsSOS1          LLSATDPVAVVALLKELGASKKLSTIIEGESLMNDGTAIVVYQLFYRMVLGRTFDAGSII
AtSOS1          LLSATDPVAVVALLKELGASKKLSTIIEGESLMNDGTAIVVFQLFLKMAMGQNSDWSSII
                ***** *****************.************ ****: ** :* :*:.     *:

McSOS1          KFLVQVSLGAVGIGLAFGVVSVLWLGFIFNDTIIEISLTLAVSYVAYFAAQEAADVSGVL
SpSOS1          KYLLQASLGAVGIGLAFGVVSVLWLGFIFNDTVIEISLTLAVSYVAFYSAQEAAEVSGVL
SbSOS1          KFLLQMALGTVPMGILFGAATNLWLAFIFHETGIETPLTLAVSYVAYFTAQEGADVSGVL
SdSOS1          KFLLQVALGAVGMGILFGAASVLWLGFIFNDTVIEISLTLAVSYVAYFTAQEGADVSGVL
SjSOS1          KFLLQVALGAVGMGIVFGAASVLWLGFIFNDTVIEISLTLAVSYVAYFTAQEGADVSGVL
CqSOS1          KYLVQVSFGAVGFGIAFGIASVLWLGFIFNDTIIEITLTLAVSYAAYFTAQEGADVSGVL
SoSOS1          KYLVQVSFGAVGFGIAFGVASVLWLGFIFNDTVIEITLTLAVSYVAYFTAQEGADVSGVL
BvSOS1          KFLVQVALGAVGMGIAFGAASVLWLGFIFNDTVIEITLTLAVSYVAYFTAQEGAAVSGVL
AhSOS1          KFLIKVSLGAVGMGIAFGALSVLWLGYIFNDTVIEITLTLAVSYVAYFTAQEGSDVSGVL
OsSOS1          KFLSEVSLGAVALGLAFGIASVLWLGFIFNDTIIEIALTLAVSYIAFFTAQDALEVSGVL
AtSOS1          KFLLKVALGAVGIGLAFGIASVIWLKFIFNDTVIEITLTIAVSYFAYYTAQEWAGASGVL
                *:* : ::*:* :*: **  : :** :**::* ** .**:**** *:::**:   .****

McSOS1          TVMTLGMFYAAAARTAFKGESQESLHHFWEMVAYIANTLIFILSGAVIAEGVLNSGHIFE
SpSOS1          AVMTLGMFFAAAARTAFKGESQESLHNFWEMVAYIANTLIFILSGAVIAEGVLNSGNIFE
SbSOS1          TVMTLGMFYAAAARTAFKGESQQSLHHFWEMVAYIANTLIFILSGAVIAQGVLSSDNIFD
SdSOS1          TVMTLGMFYAAAARTAFKGESQQSLHHFWEMVAYIANTLIFILSGAVIAQGVLSSDNIFD
SjSOS1          TVMTLGMFYAAAARTAFKGESQQSLHHFWEMVAYIANTLIFILSGAVIAQGVLSSDNIFE
CqSOS1          TVMTLGMFYAAAARTAFKGESQQSLHHFWEMVAYIANTLIFILSGAVIAQGVLSSDNIFE
SoSOS1          TVMTLGMFYAAAARTAFKGESQQSLHHFWEMVAYIANTLIFILSGAVIAQGVLSSDNIFQ
BvSOS1          TVMTLGMFYAAAARTAFKGESQQSLHHFWEMVAYIANTLIFILSGTVIAQGVLSSDSIFQ
```

```
AhSOS1          TVMTLGMFFAAAARTAFKGESQQSLHHFWEMVAYIANTLIFILSGAVIAQGVLSSDNIFE
OsSOS1          TVMTLGMFYAAFAKTAFKGDSQQSLHHFWEMVAYIANTLIFILSGVVIADGVLENNVHFE
AtSOS1          TVMTLGMFYAAFARTAFKGDSQKSLHHFWEMVAYIANTLIFILSGVVIAEGILDSDKIAY
                :*******:** *:*****:**:***:*****************.***:*:*...

McSOS1          NNGISWGYLVLLYVYVLASRAVVVTVLFPFLRYFGYGLEWKEAVILTWSGLRGAVALSLS
SpSOS1          NHGIAWGYLVLLYAYVLASRTVVVTVLFPFLRYFGYGLEWKEACILTWAGLRGAVALALS
SbSOS1          NHGIAWGYLILLYAYVLVSRVVAVGVLFPFLPYFGYGLEWKEALMLIWAGLRGAVALSLS
SdSOS1          NHGIAWGYLILLYAYVLVSRVVAVGVLFPFLXYFGYGLEWKEALMLIWAGLRGAVALSLS
SjSOS1          NHGIAWGYLFLLYAYVLVGRAIVVGVLFPFLRYFGYGLEWREALILIWAGLRGAVALSLS
CqSOS1          NHGTAWGYLILLYVYVLVARGVVVGVLYPFLCYFGYGMEWKEAMILVWAGLRGAVALSLS
SoSOS1          NHGIAWGYLILLYVYVLVARGVVVGVLFPFLRYFGYGLDWKEAMILVWSGLRGAVALSLS
BvSOS1          NHGITWGYLILLYVYVLVARGLVVGALFPFLRYFGYGLDWKEAIILVWSGLRGAVALSLS
AhSOS1          NHGISWGYLILLYAYVQVSRVVVVVVLFPFLRYFGYGLDWKEAIILIWSGLRGAVALSLS
OsSOS1          RHGASWGFLLLLYVFVQISRILVVVILYPLLRHFGYGLDLKEATILVWAGLRGAVALSLS
AtSOS1          Q-GNSWRFLFLLYVYIQLSRVVVVGVLYPLLCRFGYGLDWKESIILVWSGLRGAVALALS
                . * :* :*.***.::  .* :.*  *:*:*  ****:: :*: :* *:********:**

McSOS1          LSVKRS-SGDASLLSAQTGTLFVFFTGGIVFLTLIVNGSTTQFVLHFLGMDRLSAAKRRI
SpSOS1          LSVKRS-SGDPAHLTSRTGTLFVFFTGGIVFLTLIVNGSTTQFVLHFLGMSKLSAAKRRI
SbSOS1          LSVKRS-SGDPTLLSTQTGTLFVFFTGGIVFLTLIINGSTTQFLLRFLGMDKLSKAKRRI
SdSOS1          LSVKRS-SGDPTLLSTQTGTLFVFFTGGIVFLTLIINGSTTQFLLRFLGMDKLSKAKRRI
SjSOS1          LSVKRS-SGDPALLSTQTGTLFVFFTGGIVFLTLIVNGSTTQFLLSSLGMNKLSKAKRRI
CqSOS1          LSVKRS-SGDPAYLSTQTGTLFVFFTGGIVFLTLIINGSTTQFVLQFLGMDKLSKAKRRI
SoSOS1          LSVKRS-SGDPALLSSQTGTLFVFFTGGIVFLTLIINGSTTQFVLRFLGMDKLSKAKRRI
BvSOS1          LSVKRS-SGDPALLSTQTGTLFVFFTGGIVFLTLIINGSTTQFVLRYLGMQKLSKEKRRI
AhSOS1          LSVKSS-SSNQGPLSLQTGTLFVFFTGGIVILTLIINGSTTQFILHYLGMDKLSKAKRRV
OsSOS1          LSVKRASDAVQTHLKPVDGTMFVFFTGGIVFLTLIFNGSTTQFLLHLLGMDRLAATKLRI
AtSOS1          LSVKQS--SGNSHISKETGTLFLFFTGGIVFLTLIVNGSTTQFVLRLLRMDILPAPKKRI
                **** :  .     :.    **:*:*******:****.*******:*  * *. *.  * *:

McSOS1          LEFTKFEMEKRALEAFGDLGEDEELG-PADWPTVKRYIKSLN-SGGEQIHPHDNSTS---
SpSOS1          LEYTKFEMQKRALEAFGDLGEDEELG-PADWPTVKRYIKCLNNVDGEQIHPHDGSVD---
SbSOS1          LDFTKYEMEKKALDAFGDLGDDEELG-PADWPTVKRYIKSLNTLDGERIHPHENDGPSET
SdSOS1          LDFTKYEMEKKALDAFGDLGDDEELG-PADWPTVKRYIKSLNTLDGERIHPHENDGPSET
SjSOS1          LDFTKYEMEKKALDAFGDLGEDEELG-PADWATVKRYIKSLNTLDEERIHPHEASG-TEN
CqSOS1          LEFTKYEMEKKALEAFGDLGEDEELG-PADWPTVKRYIKSLNSIDGDRIHPHDASDN---
SoSOS1          LEFTKYEMEKKALEAFGDLGEDEELG-PADWPTVKRYIKSLNSIDGDRTHPHDVSDASDN
BvSOS1          LDFTKYEMEKKALEAFGDLGEDEELG-PADWNTVKRYIKSLNSIDGDRIHPHDASDAPDN
AhSOS1          LEFTQYEMEKKALEAFGDLGEDEELG-PTDWPTVKRYIKSLS-ISGERIHPHDASESSEI
OsSOS1          LNYTKYEMLNKALEAFGDLRDDEELGPPADWVTVKKYITCLNDLDDEPVHPHAVSDR---
AtSOS1          LEYTKYEMLNKALRAFQDLGDDEELG-PADWPTVESYISSLKGSEGELVHHPHNGSK---
                *::*::** ::** ** ** :***** *:** **: **..*.     :  *    .

McSOS1          GGDLDPMSLTDIRVRLLNGVQAAYWVMLDEGRISQSTANILMQSVDEALDSVTHEPLNDW
SpSOS1          GGDLDPMSLRDIRVRLLNGVQAAYWVMLDEGRITQTTANILMQSVDEALDSVSHEPLCDW
SbSOS1          DGYLDPMNLKDMRVRLLNGVQAAYWAMLDEGRITQNTANALMQSVDEALDKVDHEPLCDW
SdSOS1          DGYLDPMNLKDMRVRLLNGVQAAYWAMLDEGRITQNTANALMQSIDEALDKVDHEPLCDW
SjSOS1          DGYLDPMNLKDMRVRLLNGVQAAYWAMLDEGRITQNTANVLMQSVDEALDKVDYEPLCDW
CqSOS1          -GFLDPMNLKDMRVRLLNGVQAAYWAMLDEGRITQSTANVLMQSVDEALDSVDHEPLCDW
SoSOS1          GGFLDPMNLKDMRVRLLNGVQAAYWVMLDEGRITQSTANVLMQSVDEALDSVDHEPLCDW
BvSOS1          GGFLDPMNLKDMRVRLLNGVQAAYWVMLDEGRITQTTANILMQSVDEALDVVHHEPLCDW
AhSOS1          GGHLDLMNLKDMRVRLLNGVQAAYWVMLDEGRITQATASILMQSVDEALDLVDHEPLCDW
OsSOS1          NDRMHTMNLRDIRVRLLNGVQAAYWGMLEEGRITQTTANILMRSVDEAMDLVPTQELCDW
AtSOS1          IGSLDPKSLKDIRMRFLNGVQATYWEMLDEGRISEVTANILMQSVDEALDQVSTT-LCDW
                 . :.  .* *:*:*:******:** **:****:: **. **:*:***:* *    * **

McSOS1          NGLKRNVHFPNYYRFLQGSSMWPRKLVTFFTVERLESGCYICAAFLRAHRIARRQLYDFI
SpSOS1          KGLKRNVHFPSYYRFLQG-SMWPRKLVTFFTVERLESGCYICAAFLRAHRIARRQLYDFI
SbSOS1          RGLKNSVQFPTYYRFLQS-SIYPKKLVTFFTVERLESACSICAAFLRAHRIVRGQLQDFV
SdSOS1          RGLKNSVQFPTYYRFLQS-SIYPKKLVTFFTVERLESACSICAAFLRAHRIVRGQLHDFI
SjSOS1          KGLKSSVQFPNYYRFLQG-SIYPKKLVTFFTVERLESACSICAAFLRAHRIVRGQLHDFI
CqSOS1          KGLKNSVHFPKYYRLLQG-GIYPKKLVTFFTVERLESACYICAAFLRAHRTARGQLHDFI
SoSOS1          KGLKSNVHFPNYYRFLQG-SIYPKKLVTFFTVERLESACYICAAFLRAHRIARGQLHDFI
BvSOS1          KGLKSNVHFPNYYRFLQG-SMYPKKLVTFFTVERLESACYICAAFLRAHRIARGQLHDFI
AhSOS1          KGLKQNVYFPNYYRFLQR-SIYPKKLVTFFTVERLESACYICAAFLRAHRIARRQLHDFI
OsSOS1          KGLRSNVHFPNYYRFLQM-SRLPRRLITYFTVERLESGCYICAAFLRAHRIARRQLHDFL
AtSOS1          RGLKPHVNFPNYYNFLHS-KVVPRKLVTYFAVERLESACYISAAFLRAHTIARQQLYDFL
                .**:  * **.**.:*:      *::*:*:*:******.* *.*******  .* ** **:

McSOS1          GESEIATAVINESETEGEEARKFLEDVRITFPEVLRVVKTRQVTHSVLKHLIDYIHSLEK
SpSOS1          GESDIASAVISESETEGEEARKFLEDVRITFPEVLRVVKTRQVTHSVLQHLIDYIHSLEK
SbSOS1          GDSEVSFAIINESEAEGEEARKFLEDGRITFPQVLRVVKTRQATYVVLQRLIHYIESLEK
SdSOS1          GDSEVSFAIINESEAEGEEARKFLEDVRITFPQVLRVVKTRQATYAVLQHLIHYIESLEK
SjSOS1          GDSEVSFAIINESEAEGEEARKFLEDVRITFPQVLRVVKTRQATFAVLQHLIHYIESLEK
CqSOS1          GYSEISSAVITESETEGEEARKFLEDVRTTFPEVLRVVKTRQVTYAVLQHLIEYIESLEK
SoSOS1          GDSEISFAVINESEAEGEEARNFLEDVRTTFPEVLRVVKTRQVTYAVLQHLIEYIESLEK
BvSOS1          GDSEISSAIINESEAEGEEARNFLEDVRITFPEVLRVVKTRQVTYAVLQHLLHYIQSLEK
AhSOS1          GDSDISTAVINESEAEGEEARKFLEDVRTTFPQVLRVVKTRQVTHAVLQHLIDYVQSLEK
OsSOS1          GDSEVARIVIDESNAEGEEARKFLEDVRVTFPQVLRVLKTRQVTYSVLTHLSEYIQNLQK
AtSOS1          GESNIGSIVINESEKEGEEAKKFLEKVRSSFPQVLRVVKTKQVTYSVLNHLLGYIENLEK
                * *::.  :* **: *****::***. * :**:****:**:*.*. ** :*  *:..*:*
```

```
McSOS1          AGLLEEKEIHHLHDAVQTDLKRVLRNPPLVKLPKAKDLVSTHPLLGALPATARDVLVNST
SpSOS1          AGLLEEKEIHHLHDAVQTDLKRVLRNPPLVKIPKVKDLITTHPLLGALPVTARDVLVGST
SbSOS1          AGILEEKEMLHLHDAVQTDLKRLLRNPPTVKLPKVGDLITTHPFLGALPEALRNLLVGST
SdSOS1          AGILEEKEMLHLHDAVQTDLKRLLRNPPTVKLPKVGDLITTHPFLGALPEALRNLLVGST
SjSOS1          AGILEEKEMVHLHDAVQTDLKRLLRNPPTVKLPKVGDLICTHPFLGALPEGLRNRLVGST
CqSOS1          AGILEEKEMLHLHDAVQTDLKRLVRNPPTVKIPKIGELISMHPFLGALPSGVRDLLVGST
SoSOS1          AGLLEQKEMLHLHDAVQTDLKRLVRNPPTVKIPKIGELISMHPFLGALPSTVRDLLVGST
BvSOS1          AGILEEKEMVHLHDAVETDLKRLQRNPPTVKIPKVGDLITTHPFLGALPSNLRDLLVGST
AhSOS1          AGLLEEKEMLHLHDTVQTDLKRLLRNPPTVKIPKVGDLVSTHPFLGALPANVRELLVGST
OsSOS1          TGLLEEKEMAHLDDALQTDLKKFKRNPPLVKMPRVSDLLNTHPLVGALPAAMRDPLLSST
AtSOS1          VGLLEEKEIAHLHDAVQTGLKKLLRNPPIVKLPKLSDMITSHPLSVALPPAFCEPLKHSK
                .*:**:**: **.*:::*.**:. **** **:*:  :::  **:  ***    : *  *.

McSOS1          KEVVKVRGLTLYKEGSRPNGIWLISNGVVKWGSKSRVIKHAFHPTFTHGSTLGLYEVLIG
SpSOS1          KELVKVRGSTLYKEGSRPNGIWLISNGVVKWDSKTRRSKHAFHPTFTHGSTLGLYEVLIG
SbSOS1          KEEVKVRGMSLYKEGGKPNGIWLISNGVVKWASKSSKNKHSLHPAFTHGTTLGLYEVLIG
SdSOS1          KEEVKVRGMSLYKEGGKPNGIWLISNGVVKWASKSSKNKHSLHPAFTHGTTLGLYEVLIG
SjSOS1          KEEVKVRGMTLYKEGGKPNGIWLISNGVVKWASKSSKNKHVLHPTFTHGSTLGLYEVLIG
CqSOS1          KEEVKVRGMTLYKEGGKPNGIWLISNGVVKWACKVKKNKHALHQTVTHGSTLGLYEVLIG
SoSOS1          KEEVKVRGMTLYKEGGKSNGIWLISNGVVKWGSKNRKNKHALHRTFTHGSSLGLYEVLIG
BvSOS1          KEEVKVRGMALYKEGGKPNGIWLIANGVVKWASKNRTNKHALHRTFTHGSTLGLYEVMIG
AhSOS1          KEEVKVRGMTLYKEGGKPSGIWLISNGVVKWASKNRKNKHALHPTFTHGSTLGLHEVLIG
OsSOS1          KETVKGHGTILYREGSRPTGIWLVSIGVVKWTSQRLSSRHSLDPILSHGSTLGLYEVLIG
AtSOS1          KEPMKLRGVTLYKEGSKPTGVWLIFDGIVKWKSKILSNNHSLHPTFSHGSTLGLYEVLTG
                ** :* :*  **:**.:..*:**:  *:*** .:    .* :.  .:**::***:**: *

McSOS1          KPYLCDMITDSVVVCFFVEAEKILSVLGSDFEMENFLWKES-------------------
SpSOS1          KPYLCDMITDSVVVCFFIDADKILSVLGSDHDMETFLWKES-------------------
SbSOS1          KPYFCDMVTDSVAVCFFIESEKIHAVLGSDPAVEHFFWKES-------------------
SdSOS1          KPYFCDMVTDSVAVCFFIESEKIHAVLGSDPAVEHFFWKES-------------------
SjSOS1          KPYFCDMITDSVAVCFFIEAEKIQAVLGSDPAVEHFFWKES-------------------
CqSOS1          KPYLCDMITDSVAVCFYIETEKILAALGSDPAVEHFFWKES-------------------
SoSOS1          KPYLCDMITDSVVVCFFIETEKILAVLGSDPAVEHFFWKES-------------------
BvSOS1          KPYFCDMITDSVVVCFFVETEKILAVLGSDPAVEHFFWKES-------------------
AhSOS1          KPYFCDMITDSVAVCFFVEAEKILSVLGSDSAVEHFFWKEGGKRVNHRVELGREQEKLCW
OsSOS1          KPYICDMITDSVVHCFFIEAEKIEQLRQSDPSIEIFLWQES-------------------
AtSOS1          KPYLCDLITDSMVLCFFIDSEKILSLQ-SDSTIDDFLWQES-------------------
                ***:**::***:. **:::::**     **  :: *:*:*.

McSOS1          -------VIGLAKILLPQIFEKMSMHDLR-LLIER-STMNTYLSGENVEVPPHAIGFLLE
SpSOS1          -------VIALAKILLPQYFEKMSMQDLR-VLIAERSSMNIYLSGETVEVPPQSIGFLLE
SbSOS1          -------VIVLAKVLLPRVFENMSMQDMR-MLTAERSTLNTYLRGETIEVPPHSIGFLLE
SdSOS1          -------VIVLAKVLLPRVFENMSMQDMR-MLTAERSTLNTYLRGETIEVPPHSIGFLLE
SjSOS1          -------VIVLAKVLLPRFFENKSMQDMR-MLTAERSTLNTYLRGETIEVPPHSIGFLLE
CqSOS1          -------VIVLAKVLLPRVFENMSMQDMR-KLTAERSTLNTYLRGETIEVSSHSIGFLLE
SoSOS1          -------VIVLAKVLLPRVFENMSMQEMR-MLTAERSTLNTYLRGETIEVPPHSIGFLLE
BvSOS1          -------VIVLAKVLLPRVYENMSMQDMR-MLTAERSTLNTYLRGENIEVPPHSIGFLLE
AhSOS1          RTHVSESVIVLAKVLLPQVFESLSLQDMR-MLAAERSTLNTYLRGETVEVPPHSIGFLLE
OsSOS1          -------ALVVARLLLPMMFEKMATHELR-VLITERSTMNIYIKGEEIELEQNFIGILLE
AtSOS1          -------ALVLLKLLRPQIFESVAMQELRALVSTESSKLTTYVTGESIEIDCNSIGLLLE
                       .: : ::* *  :*. : :::*  :  . *.:. *: ** :*:  : **:***

McSOS1          GFIKCHSLHEELITSPAALWPAQGNSSFLSQDGTG-YKSTSFYS--QGCSYYVETRARVI
SpSOS1          GYLKTHSLTEELIMPPAALWPAQGNSSFLSQDGSA-YKSASFYYNHQGCSYYVETRARVI
SbSOS1          GFIKSHSLVEELITSPAPLWPAQGNASFLNQNGSG---YKSASFSHQGFSYYVETRARVL
SdSOS1          GFIKSHSLVEELITSPAPLWPAQGNASFLNQNGSG---YKSASFSHQGFSYYVETRARVL
SjSOS1          GFIKSHSLVEELITSPAPLFPAQGNSSFLNQNHNGSGYNKSTSFSHQGASYYVETRARVL
CqSOS1          GFVKSHPLAEELIPSPAALWPAQGNSSFLSQEGSG---YKSTSFLHQGTSYYVETRARVL
SoSOS1          GFIKSHSLVEELITSPAALWPAQGNSSFLSKMVLD---INQPAFHTREPRTMLRQELEFF
BvSOS1          GFIKSHSLVEELITSPAALWPAQGNSSFLCQDGSG---YKSTSFLHQGTSYYVETRARVL
AhSOS1          GFIKSHSFVEELITSPAVLWPARENSSFLSQESSG---YKSNNFSHRGTSYSVETRARVI
OsSOS1          GFLKTK--NQTLITPPGLLLPPNADLNLFGLESSA---INRIDYCYTAPSYQVEARARIL
AtSOS1          GFVKPVGIKEELISSPAALSPSNGNQSFHNSSEAS--GIMRVSFSQQATQYIVETRARAI
                *::*     : ** .*. * *.. : .:                        :. . . :

McSOS1          VFDINSFQHDK---TLMRRKSSLLLHDQST----ISLTSRDHGGLVSWPEN---ARPERE
SpSOS1          VFDIAAYHADKSHKTLLRRKSSLLLHDQST----MSLTR-EHGGLVSWPEN---AQSEQH
SbSOS1          LIDMSQSQPES---TLQRRKSSFLLPEQSA----RNLTNKEHSGLLSWPDTQ--YKSHQH
SdSOS1          LIDMSQSQPES---TLQRRKSSFLLPEQSA----RNLTNKEHSGLLSWPDTQ--YKSHQH
SjSOS1          LIDMSQSQPES---TLQRRKSSFLLPEQSA----RNLTNREHSGLLSWPENQ--YRSHQQ
CqSOS1          LIDMVPIQADN---TLLRRKSSLLLHDQSS----RSLSSRDHAGLLSWPENQ--YKSYQH
SoSOS1          XLTWLQRKLII---LSSGGXSSVLLRDQSS----RSLAR-EHGSLLSWPEHQ--YKSHHR
BvSOS1          LIDMAATPADS---TLQRRKSSILLRDQSS----RSLIR-DHAGLVSWPENH--YKSHQQ
AhSOS1          LMDMATLQADS---TLQKRKSSLSLHEQSS----RSLTR-EHGSLLSWPEHH--YNSHQH
OsSOS1          FVEIGRPEIEAD----LQRSASLISQTLEL----PRTQSKEHSGLLSWPESFRKSRGAQN
AtSOS1          IFNIGAFGADR---TLHRRPSSLTPPRSSSSDQLQRSFRKEHRGLMSWPENIY-------
                 .                  :*.     .           :* .*:***:
```

```
McSOS1          GEDEEEIDEHEQNMSARARQLSIYG-------SMLKEKDPLLKTVSFHESVLNKPSSHSL
SpSOS1          QQDEEDPDEDEHNLSAKAMQLSIFG-------STVKQP---LYKAASFQDIGQNKGAHSL
SbSOS1          LPVGEEIEDDEN-LSAKAMQLSIYG-------SMVKDAPIRGQSFKGDNLGNP--SRSYP
SdSOS1          LPVGEEIEDDEN-LSAKAMQLSIYG-------SMVKDAPLRGQSFKGDNLGNP--SRSYP
SjSOS1          LPTGQEIEESEN-LSAKAMQLSIYGSMVPSRGESFKEAPMRGQSFKGDNLGNPSHVRSYP
CqSOS1          LPDGQEIDDSQN-LSAKAMRLSIYG-------STAKDVPVRGLSFQGYTLGNPSHVRSYP
SoSOS1          LP-GQEIEDTQN-LSAKAMHLSIYG-------STVNVVPSRGYSLKGDTLGNPSHFRSYH
BvSOS1          LPDGKEIDDNQS-LSAKAMQLSIYG-------STVKDAPTRGFSFQGDNLGNPSHFRSYH
AhSOS1          LADDKETDENRNNLSAKAMQLSMFG-------STVRDAPFRTRSFQGYTGGNPSHIHSYP
OsSOS1          GASLTEIRDHPASFSARALQLSMYGSMINDMKSGQGQGQRRQRHRHTKASSNKAHSSSYP
AtSOS1          AKQQQEINKTTLSLSERAMQLSIFG--------SMVNVYRRSVSFGGIYNNKLQDNLLYK
                     :  .    :* :* :**::*

McSOS1          SYPTVPDAQGRSLTTVKSEGSHTIRKRLEGDLPDIPSVPHS--------RRPSQLP--ES
SpSOS1          SYPKIPETQGRTLTSVKSEGSTTVRKRLAEELAGKLPPPSH--------SRKQSRAQEES
SbSOS1          HVPFSDVEQPRTLTSVKSEGSTAVRKKHEEDVIRQELLPP--L----HS-RQPSRAVDDS
SdSOS1          HVPFSDVEQPRTLTSVKSEGSTAVRKKHEEDVIRQELLPP--L----HS-RQPSRAVDDS
SjSOS1          QVPFSDQEQ-RSLTSVKSEGSTAVRKRLAEEGIKQELLPP--A----HSSRHPSHARDDS
CqSOS1          QVPIG--QKQLPLTSVKSEGSNTVRKRLGEDVMREELLPP-------THSRHPSRAVDDS
SoSOS1          QVPES--QEPRGLPSVKSEGSNTV------------------------------------
BvSOS1          HVPVG--PEQRPLTTVKSEGSNTVRKRLEDDVMRQGLLPPSGP----SHSRHPSRAVDDS
AhSOS1          RITLG--QDRRPLTSAKSEGSTTVRKRLEEEVTRKELLPTS-------HTRKPSQARDDS
OsSOS1          RVPSRSSNTQRPLLSVQSEGANMTTARQAAAAGASLPPEPEEAGRRRRRQRKAIEEDEDN
AtSOS1          KLPLN---PAQGLVSAKSESSIVTKKQLETRKHACQLPLKG-------ESSTRQNTMVES
                  .         * :.:**.:

McSOS1          SDESGGEEDVIVRIDSPSRLSFRHAP----------------------------------
SpSOS1          SDESGGEDDLIVRIDSPSGLTFPRPAP---------------------------------
SbSOS1          SSESGGDDDVIVRIDSPSNLTFRQAP----------------------------------
SdSOS1          SSESGGDDDVIVRIDSPSNLTFRQAPXWDFLPTPIVLLYFCFLSYPVIFILPICMISYVY
SjSOS1          SSESGGDEDVIVRIDSPSKLSFRQAP----------------------------------
CqSOS1          SSESGGEDEVFVRIDSPSKLSFRQAP----------------------------------
SoSOS1          ------------------------------------------------------------
BvSOS1          SSDSGGEDDVIVRIDSPSKLSFRQAP----------------------------------
AhSOS1          SSESGGEDDVVVKIDSPSNLSFRQAP----------------------------------
OsSOS1          SSDESAGEEVIVRVDSPSMLTFRQPSSAADR-----------------------------
AtSOS1          SDEEDEDEGIVVRIDSPSKIVFRNDL----------------------------------
```

Supplementary Figure S6. Alignment of SOS1 protein sequences. The multiple sequence alignment of SOS1 protein sequences was generated with ClustalW (http://www.ebi.ac.uk/Tools/msa/ClustalW2/). Identical amino acids are marked with an asterisk (*) below. Protein accession numbers and species names used for amino acid sequences alignment are showed on Supplementary Table S1.

```
OsHKT1_5        ---------------------------------------------MSSLDATTPRYDEFK
OsHKT1_4        ------------------------------------------------------------
SsHKT1_1        --------------------------MLSFKFIVEKCKHFYTSLYLLLVYVFTSLYWLIS
SbHKT1_1        -------------------------MLSINNIVVNKYKHLCNSLYLIILHLFSSLHWISS
BvHKT1_3        --------------------------MLRYKFLVEKYKHFYSFLSICVVYLITSICWITS
AH014429-RA     ------------------------------------------------------------
McHKT1_2        --------------------------MGRFGFLKEKVQQLYGCLCVGLFYLLSTLFWVSN
BvHKT1_2        ----------------------MQLLPHTHKMMEKYLVFLHEKLDDLLKIYHKKVEPFFS
CqHKT1_2        ----------------------MQLHHHLQEMMEKYLVIQHEILDQIISFFHKNIKPYIS
McHKT1_1        --------------------------------MEKYLALFHKKLDQFFNFCYTKALSYIS
BvHKT1_1        ---------------------------MNYFAFLANKLAHLCKHYFPKRPTFTTSYSLKN
CqHKT1_1        ---------------------------MNYFSSLGNKFKQLCIEYYHKKHPFLLSS-MKT
AhHKT1_1        ------------------------------------------------------------
OsHKT1_1        MHPPSLVLDTLKRIKLYIAMKLLLPNSEVLRIYWEKAQHLCGFLSMKLISRARCVASSVK
OsHKT1_3        -------------------------MNHCLVVSHKKLQTFRTFAASKFSSFTKSAQKSIK
AtHKT1_1        ---------------------------------------------------MDRVVAKIA
OsHKT2_1        --------------------------------MTSIYHDFIHNKLQSFGRIGRYFVNFVV
OsHKT1_2        ------------------------------------------------------------

OsHKT1_5        RIYHLFLFHAHPFWLQLLYFLFISLLGFLMLKALP------MKTSMVPRPMDLDLIFTSV
OsHKT1_4        MPTSRRALAGGALSMHVAYFLAISCLGYGLLGVLK------VRE-PGAAPRRIDRFFTAV
SsHKT1_1        KIYDFIMVYVCHLIIELCYFILVSSFGFLFLKTLIPKSSNNNNNNNS--INDLDLFFTSV
SbHKT1_1        KTCDFIIINISHFIIELCYFILVSSFGFLILKTLNPKSIHNNNNHIINPIKDLDLFFTSV
BvHKT1_3        KINEFIISHTTHFMIEVFYFILVSICGFVSLKNIDPRTG-LLSSYPNKNIRDIDMFFTSV
AH014429-RA     ------------------------------------------------------------
McHKT1_2        KIYDFITYRMSHFSIEVCYFIFVSCLGFLILRNIKPRTYPVKP-------GELDMFFTSV
BvHKT1_2        HLFEYLLFQINPYWHHLFYYILVSLLGYISLKGS---KQNSSVSSNPHY--DLDLIFTSV
CqHKT1_2        HGFEYLLFQINPYWHHLFYYITISFLGYISLKGT---KQSSSLASNPHHDLDLDLIFTSV
McHKT1_1        SLYEYIIFQVHPFWHHLFYYIIVSLLGYFSLKATS-RKQRSSPVFSDPRHDDLDLFFTSV
BvHKT1_1        SMFHYIISHAKPFWLHLTYFIVVSLLGYMALKVS-------KPNTNANDLSNFDLFFTSV
CqHKT1_1        SMFNYIISHAKPFWLHLCYFLVVSLIGVMALKVS-------KPRTDVDQLTSLDLFYTAV
AhHKT1_1        ------------------------------------------------------------
OsHKT1_1        QSYSFLVCKSNPLVVQLVYFVIISFAGFLALKNLK-------PQGKP-GPKDLDLLFTSV
OsHKT1_3        YSFQFIYQ-NNPLFVHVAYFALISFAGYGSLKVLK-------PRDKSNTLKDLDVLFTSV
AtHKT1_1        KIRSQLTKLRSLFFLYFIYFLFFSFLGFLALKITKP--------RTTSRPHDFDLFFTSV
OsHKT2_1        LAHRFIALHIHPFWIQLSYFLLISILGSVLLMFLK-------PSNPEFRPGYIDMLFLST
OsHKT1_2        -----------------------------------------------------------M

OsHKT1_5        SATTVSSMVAVEMESFSNSQLLLITLLMLLGGEVFTSILGLYFTNAKYSKMIPTLPDDDD
OsHKT1_4        SAATVSSMSTVEMEVFSNGQLVVLTVLMLLGGEVFVSLVGLASKWSKLRSDAMDRSRRVE
SsHKT1_1        SATTVSSMSTLEMEVFSNSQLIVLTILMFIGGEVFTSMVGLHFSASKLVYTPLQARSRVN
SbHKT1_1        SATTVSSMSTLEMEVFSNSQLIVLTILMFIGGEVFTSMIGLHFSASKLVYKPLKSRSRVN
BvHKT1_3        SATTVSSMSTIEMEVFSNSQLVIITILMFIGGEVFTSMVGLHFSASKLTYTPLSARSRVN
AH014429-RA     -------MSTVEMEVFSNTQLIILTILMFIGGEIFTSMVGLHFSASKLKYTPK-ARSRVN
McHKT1_2        SAATVSSMATVEMEVFSDAQLHIMTILMFIGGEVFTSMVGLHFKASRLGNTPLGVKSRAN
BvHKT1_2        SATTISSMSTIEMENFSSTQLGVLVILMFSGGEVFLSLLGLQIRKLKHK-----RRARNH
CqHKT1_2        SATTISSMSTVEMEDFTSTQLIVLVILMFSGGEVFLSLLGLQIRRLKHK-----RRARNH
McHKT1_1        SATTISSMSTIEMEDFSSPQLVVLIILMLSGGEVFLSLLGLQLRKSKHR-----KREQKN
BvHKT1_1        SASTVSSMTALEMEVFSNDQLIVMTILMILGGEVFTSMLGLLLRRCKSL-----SNESKK
CqHKT1_1        SASTVSSMSAVEMEVFSNYQLVVMTILMLLGGEVFTSMLSLQLRRFKFS-----SYESTK
AhHKT1_1        ------------------------------------------------------------
OsHKT1_1        STLTVSSMATVEMEDLSDRQLWVLILLMLMGGEVFTSMLGLYFNNANAN-------RNEN
OsHKT1_3        SASTVSSMATVEMEDFSSAQLWVLTILMLIGGEVFTSMLGIHFMRAEFG-------TKES
AtHKT1_1        SAITVSSMSTVDMEVFSNTQLIFLTILMFLGGEIFTSFLNLYVSYFTKFVFPHNKIRHIL
OsHKT2_1        SALTLSSLITIEMEVLSSSQIVVITLLMLLGGEVFVSFLGLMLRLNHKHNPEFSGDK---
OsHKT1_2        AVDLMGCYAPRRADDQLAIQEAATAGLRSLEMLVSS------------------------

OsHKT1_5        HGGNGKPPPPTTSPSSTLVELELAPPMDVVVVNPTTTATTHDEVELGLGRRNKRGCTCTT
OsHKT1_4        SHG----------------DVALADIDGGDVENPTS------------------SGEEAAS
SsHKT1_1        SVA------------------------SLPLPPESIELGVIIPSSTT-TQEIRVSSSTIE
SbHKT1_1        SVA------------------------SLTLPCKPIELGLIVVTP-----QENNTSTTMQ
BvHKT1_3        SVA------------------------NLPLPLESIELGVIIPCSS---TQEISNNTTME
AH014429-RA     SVA------------------------SLPLPPESLDLGIITQSSTTITQDIITSSTTME
McHKT1_2        SVA------------------------SLPCPPEDFDHIELGIITTT-TTTTTTTTTTLQ
BvHKT1_2        VLN------------------------PSPASEGDGMKFRS-------------------
CqHKT1_2        ALN------------------------PNPTSEGEGIKSKT-------------------
McHKT1_1        ILL------------------------NSPS---EGMKYKS-------------------
BvHKT1_1        IDN------------------------TSQPTINSIELGTINQMLPLPSNTRGSSFNIES
CqHKT1_1        VDN------------------------SKG--EESIELGIINQFSSTSNDNNKSYS----
AhHKT1_1        ------------------------------------------------------------
OsHKT1_1        SQR------------------------SLPSISLDIESNSPANNGDHKITECGQSEETMS
OsHKT1_3        VST------------------------RDHSPCIDIESITSTKFG--PSTQGTKVTVSFS
AtHKT1_1        GSY------------------------NSDSSIEDRCDVETVTDYR--------------
OsHKT2_1        ----------------------------VSSVPIELDTINSASTVISCEELQLEAAIPEV
OsHKT1_2        ------------------------------------------------------------
```

```
OsHKT1_5         THTSSSPSASKTTTTRLLMFVVMGYHAVVHVAGYTAIVVYLSVVGGAGAVVAGKGISAHT
OsHKT1_4         RRRPMDADTLRHNAVRALFYIVLAIFAVVHVVGAVAVAAYVLASPGARRTLGDKSLNTWT
SsHKT1_1         KTKSEIDFLIKSKSIRVLGFVVLSYLIIVHFLGISMVLTYINTIPNAKNVLDKKGLKTFT
SbHKT1_1         KTKSEIDFFIKSKSIRVLGFLVLLYLFTIHILGISMVLLYLNITPNAINVLHKKGIETFT
BvHKT1_3         KTKSEIDFLIKSKSTRVLGLLVLGYLLIVHILGISMVFAYLNIFSSAHKILEKKGLKTFT
AH014429-RA      KTKSEIDFLIKSKSVKVL--------------------VYVNIISSVKNVLKKKGLKTFT
McHKT1_2         KTKSEIDFLLKSRSIRVLGFVVLAYLLIVHVLGTIMVYAYLRIEPSAKRVLETKGLKSIT
BvHKT1_2         --------------LRALNHVVLGYLLVSHILGYSLVSLYFSIVPSASNVLARKKIETHF
CqHKT1_2         --------------LRVLNHVVLGYILVSHILGYISVSLYMNIISSANNVHVIKKIDSHL
McHKT1_1         --------------LVALGHVVLGYLLVCHIIGYSLASLYISISRSASNVLERKHIEMHL
BvHKT1_1         FISADDWSSKYNNSIKCLGYVVLGYIITMHLVGSTLVTMYISLTPSASNVLERKGLNLHT
CqHKT1_1         -------SFNYNYSIKLLGYVVLGYMISLLTIGSSLVTMYMILTPSANHVLKNKRLILHT
AhHKT1_1         --------------------------------------MYISLTPSAKHVLESKGLVLQT
OsHKT1_1         QNQVQQNKSITYNPCAVLVRIVTGYFVATVISSSVIIIIYFWIDSDARNVLKSKEISMYT
OsHKT1_3         ELRMENGGHVEPKTIKFLGFVVMGYLLITNLGGSLLIYLYLNLVPSAHKILKRKGIGIIV
AtHKT1_1         ----EGLIKIDERASKCLYSVVLSYHLVTNLVGSVLLLVYVNFVKTARDVLSSKEISPLT
OsHKT2_1         PSSTIKDLKRSKRLRWFLGFVVFSYFVVIHVAGFLLVLWYISRVSSAKAPLKKKGINIAL
OsHKT1_2         ----------------------------------------LSSSSQAAGAHKASPQQQPFG
                                                               .

OsHKT1_5         FAIFTVVSTFANCGFVPTNEGMVSFRSFPGLLLLVMPHVLLGNTLFPVFLRLAIAALERV
OsHKT1_4         FAVFTTVSTFSNCGFMPTNENMVVFKRDAPLQLLLVPQVLAGNTLFAPLLAACVWAAAAA
SsHKT1_1         FSIFSVVSTFASCGFIPTNENMQVFSKNSGLLLMLIPQILLGNTLFPSFLRFSIWMLGKF
SbHKT1_1         FSIFTIVSTFASCGFIPTNENMQVFSKNSGFLLILIPQILLGNTLFPSFLRFSIWVLGKF
BvHKT1_3         FSLFTIVSTFASCGFIPTNENMQVFSKNSGLLLILIPQILLGNTLFPSFLRFSIWVLGKF
AH014429-RA      FSIFTIVSTFASCGFIPTNENMLVFSKNSGLLLILIPHNLLGNTLFPSFLRFSIWVLSKI
McHKT1_2         FAIFTSVSTFSSCGFVPTNENMIIFRQNSGLLLMLIPQVLLGNTLLPSFLRLTIWVLGKF
BvHKT1_2         FSLFTTVSTFANCGFIPTNENMIPFKKNSGLLLILIPQILMGNKLYPSCLRLVIWVLEKV
CqHKT1_2         FSIFVIVSTFANCGFIPINENMMPFNKNSGLLLILIPQILMGNKLYPSCLRVVIWVLEKF
McHKT1_1         FSLFVTVSTFSNCGFIPTNENMMVFKGNSGLLLILIPQILLGNKLYPSCLRLVLWVLEKL
BvHKT1_1         FSFFIVVSTFASCGFAPTNENMMVFRKNPGLLLILMPQVFLGNSLYPPFLRFVIWVLERF
CqHKT1_1         FSFFVIVSTFSSCGFVPTNENMMIFRQNPGLLIIIPYLFLGNSLYPVFLRSVTWVLERF
AhHKT1_1         FSFFVIISTFSNCGYIPTNENMIVFRKNSGLLLILIPQIFLGNTLFSPCLRLVIWVLERF
OsHKT1_1         FCIFTAVSSFANCGFTPLNSNMQPFRKNWVLLLLVIPQILAGNTLFSPLLRLCVWVLGKV
OsHKT1_3         FSVFTAISSVGNCGFTPVNENMIIFQKNSILLLLILPQILAGNTLFAPCLRLMVWSLEKI
AtHKT1_1         FSVFTTVSTFANCGFVPTNENMIIFRKNSGLIWLLIPQVLMGNTLFPCFLVLLIWGLYKI
OsHKT2_1         FSFSVTVSSFANVGLVPTNENMAIFSKNPGLLLLFIGQILAGNTLYPLFLRLLIWFLGKV
OsHKT1_2         EIADQAVSKFRKVISILDRTGHARFRRGPVESSAPAAPVAAAPLPPPPPPAPVAAALAPT
                       :*.. .      . .   *                .    .

OsHKT1_5         TGWPELGELLIRRRRGGGEGYDHLLPSSRTRFLALTVAVLVVAQLALFCAMEWGSDGLRG
OsHKT1_4         TRREELVEMAREGGRAAAAGYAHLMPARRCWMLAATVAAFVAVLMALVCGMEWGG-ALQG
SsHKT1_1         VKKDETKYLMRNSKE---IEYHHLLSSKHSRFLIVTVLGFILVQFIMFCSMEWNFDGLNN
SbHKT1_1         AKKDEAKYLLRNAKE---IGYHHLLPSKHSRLLIMTVLGFIMVQLVMFCAMEWGNEGINE
BvHKT1_3         TKKDETKYLMRNSKE---IGYHHLLPSKHSKLLVVTVFGFILVQFILFCAMEWSFEGFNG
AH014429-RA      GKKDEAKFLMRNTKE---IGYHHLVPSKHSKLLVVTVLGFILVQFVMFCGMEWRNEGLNG
McHKT1_2         TKKDESKYLMRNTKE---IAYHHLLPTKHSKYVVVTVFGFILASLIMFCSMDWNLKGLSD
BvHKT1_2         TKKEEYSFMLQNHGG---LGYGNLTSSYKAFLLGITAIGLVMVQFVVFGILEWNALVLEG
CqHKT1_2         TGKEEYNYMLRNHDD---LGYGNLISSFKAFLLSFTAIGLVMIQFLVFTTLEWNSLVFQG
McHKT1_1         TGKAEFSYILKKNDE---LGYGLFFSQVDALLLAVTAVGLVMVQFVVFCILEWNSVALMD
BvHKT1_1         TKRKEFDYMLNNYQE---LKYGHLLSSKKCWYLTATTLAFVVLQFALFCAMEWSSGVMEG
CqHKT1_1         TKRKEFYYMLNNYQE---MGYDHLMSSKKTWFLGATTIGFWVLQIAVFSGMEWSSGVMEG
AhHKT1_1         TKKKEYNYMLNNYKE---MGFDGLMCSKKSWFLVGSVFGLYMLQIVVFSGMEWSSGVMEE
OsHKT1_1         SGKAEYAYILQHPGE---TGYKHLHVRRNSVYIVLSVTGLILLQVMFICSFEWNSESLEG
OsHKT1_3         TGKKDCRYILEYPKA---IGYKHLMSTRESVYLTLTVVSLIILQTVLFLSLEWSSVALDG
AtHKT1_1         TKRDEYGYILKNHNK---MGYSHLLSVRLCVLLGVTVLGFLIIQLLFFCAFEWTSESLEG
OsHKT2_1         TKLRELKLMIKNPEE---LQYDYLLPKLPTAFLASTVIGLMASLVTLFGAVDWNSSVFDG
OsHKT1_2         SSQPQTLTLDFTKPN---LTMSAATSVTSTSFFSSVTAGEGSVSKGRSLLSSGKPPLSGH
                     :   :                       .   .              .

OsHKT1_5         -LTAGQKLVGALFMAVNSRHSGEMVVDLSTVSSAVVVLYVVMMYLPPYTTFVPVQDKHQK
OsHKT1_4         -MSPWEKVVNALFLAVNARHTGESTVDLSILAPAILVLFVLMMYLPPYTTWFPFEENSTT
SsHKT1_1         DHNIYQKLVGILFQCVNSRHTGESIVDLSSINSAMLVIFIVMMYLPPYTSFLPIKDEEKE
SbHKT1_1         GHNVYQKLVGILFQCVNSRHTGESIVDLSSIAPAMLVVFIVMMYLPPYTSFVPIKDKEKE
BvHKT1_3         -LNVYQKLVGVLFQCVNSRHTGESIVDLSSIASAILVLFIVMMYLPPYTSFVPIKDEEGE
AH014429-RA      -LNVYEKIVGILFQCVNTRHTGESIVDLSSISSAILVLFMAMMYLPPYTSFLPIEDEEED
McHKT1_2         -LNVYQKLVGALFQCVNARHTGETIVDLSTIASAVLVVFVIMMYLPPYTSFLPTKDGEEE
BvHKT1_2         -QNGYQKFVGSLFQTVNSRHSGESIVDISLVSHATLVLFVVMMYLPSTTTFVPISYNKES
CqHKT1_2         -QNWYTKIVGSLFQTVNSRHSGESIVDVSLLQPATLALFVVMMYLPSTTIFVPIGYDKES
McHKT1_1         -LNWHQKLVGSLFQTVNSRHSGESIIDLSLISPATMVLFVVMMYLPSYTTFIPVGYDKEI
BvHKT1_1         -MSSYQKIVGSFFQTANIRHSGESIVDISLLSGAVLVLFTVMMYLPAYTSFLPINDKQEE
CqHKT1_1         -MSSYEKFVGTIFQTASSRHSGEYIVDLSIISPVVLVLFIIMMYLPAYTSFLPVSNEHQE
AhHKT1_1         -MGWYEKFVGSLFQTANSRHSGESIVDLSMVS----------------------------
OsHKT1_1         -MNWLQKLVGLLFQSVNTRQAGESILDISTLSPSTLLLFAVVMYLPSDASFLTANADNQP
OsHKT1_3         -MSNYQKIVSALFQSVNARHAGESVTDLSNLSSAILVLYTIMMYLPGYTSFLPRHDGEDS
AtHKT1_1         -MSSYEKLVGSLFQVVNSRHTGETIVDLSTLSPAILVLFILMMYLPPYTLFMPLTEQKTI
OsHKT2_1         -LSSYQKIINALFMAVNARHSGENSIDCSLIAPAVLVLFIILMYLPPSTTFALSNGDEKT
OsHKT1_2         KRKPCAGGHSEATANGGRCHCSKRRKNRVKRTIRVPAISSKIADIPPDEYSWRKYGQKPI
                          .      .  :  .:   :
```

```
OsHKT1_5        TGAQSG-------QEGSSSSSIWQKLLMSPLSCLAIFIVVICITERRQIADDPINYSVLN
OsHKT1_4        KDSNAE-------NQG---IRLLESTLLSQLSYLTIFVIAICITERRKLKEDPLNFSVLS
SsHKT1_1        YPN------MVLFNREKKRRKILKNFLFSQLGYIAIFIIIICITEKQKIKDDPLNFNVFN
SbHKT1_1        YT-------HILCKEEDKSRKILKNVLFSQLSYLAIFTIIICIIEKQNMKDDPLNFNVFN
BvHKT1_3        -------LPQILCKSEKKSKIMLKNIIFSQLSYLAIFIMIICITERQNIKDDPLNFNIFN
AH014429-RA     QYPHI--IYPCKKHVEKKRKKLFKNLILSQLSYIVIFIIIICIIERQRIKDDPLNFNVFN
McHKT1_2        YP-------LVYKGEKTKGKLILDNVVFSQLSYLVIFIILVCITERKSMKEDPLNFNVLN
BvHKT1_2        ALIENSSQSQTSRSSKKQGSSILENLKFSPLSYLAIFVILVCITESKSLKEDPINFSVFN
CqHKT1_2        TLLAENRQSKSSKK-QKQESNILKNLTFSPLSYLAIFVMLICITEQSSLKQDPLNFTVFN
McHKT1_1        SPEMSSKKSK------KQGCSIAENLKFSQLSYLAIFVMIVCITERRNLVEDPLNFNVFN
BvHKT1_1        ALKNN------ATMKKQNKKKIVENMLFSQLSYLVIFVIVICITERKSLKEDPLNFNVLN
CqHKT1_1        EVPESN----IITKKQNEKKRIIENMLLSQISYLVIFIMLVCITERKSLKEDPLNFSVLN
AhHKT1_1        --------------------------------------------------------SAIL
OsHKT1_1        LTDKKTN---------SISRALWRNFTVNKLSCLAMFTFLACITERKSISSDPLNFNIFS
OsHKT1_3        KTEKIN-----------KRKGLLENWIFSHMSYLAIFVMLICITERDSMATDPLNFNVFS
AtHKT1_1        EKEGGD------DDSENGKKVKKSGLIVSQLSFLTICIFLISITERQNLQRDPINFNVLN
OsHKT2_1        ANKKAK----------RKLGLVVQNLAFSQLACISVFVIVAFITERSRLRNDPLNFSALN
OsHKT1_2        KGSPYP------------------------------RGYYKCSTVRGCPARKHVERATDD


OsHKT1_5        IVVEVISAYGNVGFSTGYSCARQVRP--DGSCRDLWVGFSGKWSKQGKLTLMAVMFYGRL
OsHKT1_4        IVVEVVSAYGNVGFSMGYSCSRQINP--DHLCTDKWTGFVGRWSDSGKLILIFVMFFGRL
SsHKT1_1        IAFEVISAYGNVGFSTGYSCEKQLKG--DPNCVNKLYGFLGSWSDEGKLVLIIVMLFGRL
SbHKT1_1        IAFEVISAYGNVGFSTGYSCGKQLKG--DPKCVDKWYGFAGRWSDQGKLVLILVMFFGRL
BvHKT1_3        IAFEVISAYGNVGFSTGYSCGRQLKV--DSTCVSKWYGFAGYWSDQGKIVLIIVMFFGRL
AH014429-RA     IAFEVV------------------------------------------------------
McHKT1_2        IVVEVISAYGNVGFSTGYSCSRQLKP--DANCVDKWYGFVGKWSDQGKIILIFVMFFGRL
BvHKT1_2        IIVEVVSAYGNVGFSMGYSCKRRLEN--FSNCKDSWYGFAGRWSWEGKFLLILVMLFGRL
CqHKT1_2        IIVEVVSAYGNVGFSMGYSCKRRLDN--FSNCKDSYYGFVGRWSWKGKFLLIFVMLFGRL
McHKT1_1        IIVEVISAYGNVGFSMGYSCKRRLGD--IGTCKDAGYGFVGRWSRQGKMVIILVMLFGRL
BvHKT1_1        IVFEVISAYGNVGLSTGYSCKRRLKI--DGNCEDKFYGFCGRWSNGGKFTLILVMFFGRL
CqHKT1_1        IVFEIISAYGNVGFSTGYSCKRRLTT--DKYCEDKLYGFSGRWSNGGKCVLILVMFFGRL
AhHKT1_1        VVFIIMIAYGNVGFSTGYSCERRLKT--KEFCEDKMYGFVGKWSNGGKAMLILVMLFGRL
OsHKT1_1        IVFEIISAFGNVGYSLGYSCQKLLKP--DATCKDASYGFVGRWTEEGKLIVILVMFLGRL
OsHKT1_3        ILFEVVSAYGNVGFSVGYSCKRLLNH--DARCKDASYGFAGKWSDNGKAILIIVMLFGRL
AtHKT1_1        ITLEVISAYGNVGFTTGYSCERRVDIS-DGGCKDASYGFAGRWSPMGKFVLIIVMFYGRF
OsHKT2_1        MIFEIISAYGNVGLSTGYSCSRLQKLHPGSICQDKPYSLSGWWSDEGKLLLVFVMLYGRL
OsHKT1_2        PAMLVVTYEGEHRHTPGP--------------------------LPAPPAAAAVAAMPVS
                  . ::

OsHKT1_5        KKFSLHGGQAWKIE----
OsHKT1_4        KKFSMKGGKAWKLS----
SsHKT1_1        KKFNLKGGKAWKLL----
SbHKT1_1        KKFNLKGGKAWKLL----
BvHKT1_3        KKFNLKGGKSWKLL----
AH014429-RA     ------------------
McHKT1_2        KKFNMKGGRAWKLL----
BvHKT1_2        KRFHFQSGKAWKLSL---
CqHKT1_2        KRFHFNSGNAWKLTF---
McHKT1_1        KRFNFKGGKAWKLSL---
BvHKT1_1        KIFNMHGGKAWTML----
CqHKT1_1        KKFNMHGGKAWKIL----
AhHKT1_1        KKFNLHGGKAWIPLS---
OsHKT1_1        KEFILK------------
OsHKT1_3        KTFNMKGGRAWKLR----
AtHKT1_1        KQFTAKSGRAWILYPSSS
OsHKT2_1        KAFTKGTGEYWRLW----
OsHKT1_2        VAVSTGNGHV--------
```

Supplementary Figure S7. Alignment of HKTs protein sequences. The multiple sequence alignment of HKTs protein sequences was generated with ClustalW (http://www.ebi.ac.uk/Tools/msa/ClustalW2/). Identical amino acids are marked with an asterisk (*) below. Protein accession numbers and species names used for amino acid sequences alignment are showed on Supplementary Table S1.

```
BvNHX1          MMEQLSSVFFSKMNSLSTSDHASIVSMNLFVALLCG-CIVLGHLLEENRWMNESITALLI
SeNHX1          MLSQLSSLFYSKMDMLSTSDHASVVSMNLFVALLCG-CIVIGHLLEENRWMNESITALLI
SsNHX           MLSQLSSFFASKMDMVSTSDHASVVSMNLFVALLRG-CIVIGHLLEENRWMNESITALLI
CqNHX1          MWSQLSSLLSGKMDALATSDHASVVSMNMFVALLCG-CIVIGHLLEENRWMNESITALLI
SoNHX1          -------------------DHASVVSMNLFVALICG-CIVIGHLLEENRWMNESITALLI
AhNHX1          -MMLLLSSLFSKIDALTTSDHGSVVSMNMFVALLCG-CIVIGHLLEENRWMNESITALLI
AtNHX1          ----MLDSLVSKLPSLSTSDHASVVALNLFVALLCA-CIVLGHLLEENRWMNESITALLI
OsNHX1          --MGMEVAAARLGALYTTSDYASVVSINLFVALLCA-CIVLGHLLEENRWVNESITALII
GmNHX3          -MWSQLSSVVSKLQTLSTSDHASVVSMNLFVALLCCGCIVIGHLLEEN-WMNESITALLI
BvNHX2          ----MVLDLGVQIANMGESGHAQVVPIAVFVAVLCF-CMVIGHLLEENRWVNESITAILI
BvNHX3          -----MFGVPNIVHESSPFDTSTVISITVFISLICL-CIIVGHLLEENRWANESITSLLL
AtNHX3          -----------------------------------------GRMMNCPKILRQLGSKVLL
                                                         *::::     .:  : :::

BvNHX1          GLSTGVVILLISGGKSSHLLVFSEDLFFIYLLPPIIFNAGFQVKKKQFFRNFITIIMFGA
SeNHX1          GLCTGVVILLISGGKSSHLLVFSEDLFFIYLLPPIIFNAGFQVKKKQFFRNFITIIMFGA
SsNHX           GLSTGIIILLISGGKSSHLLVFSEDLFFIYLLPPIIFNAGFQVKKKQFFRNFITIILFGA
CqNHX1          GLATGVVILLISGGKSSHLLVFSEDLFFIYLLPPIIFNAGFQVKKKQFFRNFITIILFGA
SoNHX1          GLATGVIILLISGGKSSHLLVFSEDLFFIYLLPPIIFNAGFQVKKKQFFRNFITIILFGA
AhNHX1          GLATGVVILLISGGKSSHLLVFSEDLFFIYLLPPIIFNAGFQVKKKQFFRNFITIMMFGA
AtNHX1          GLGTGVTILLISKGKSSHLLVFSEDLFFIYLLPPIIFNAGFQVKKKQFFRNFVTIMLFGA
OsNHX1          GLCTGVVILLMTKGKSSHLFVFSEDLFFIYLLPPIIFNAGFQVKKKQFFRNFMTITLFGA
GmNHX3          GVCTGIVILLFSGGKSSHILVFSEDLFFIYLLPPIIIFNAGFVKKKQFFVNFMTIMIMFG
BvNHX2          GCLTGMIILLISKGKNSHIVRFDEELFFIYLLPPIIFNAGFQVKKKQFFQNFLTIMLFGV
BvNHX3          GVVSGAVVLLIRRGQNSRILVFNEELFFIYLLPPIIFNAGFQVKKKQFFKNFSTILSFGV
AtNHX3          PLTYERINKSMN--KSIHIVVTMAKSLENSVENKIVSLDPSEAGPPRYLG----------
                          :   :. ::.    . :   :   *:      .   :::

BvNHX1          IGTLISFTIISLGAMTIFKEMDIGSLELGDYLAIGAIFAATDSVCTLQVLNQDETPLLYS
SeNHX1          IGTLVSFSVISLGAMTIFKKMDIGSLELGDYLAIGAIFAATDSVCTLQVLNQDETPLLYS
SsNHX           VGTLVSFIIISLGSIAIFQKMDIGSLELGDLLAIGAIFAATDSVCTLQVLNQDETPLLYS
CqNHX1          VGTLVSFTIISFGALSIFKKLDIGSLDLADYLAIGAIFAATDSVCTLQVLNQDETPLLYS
SoNHX1          IGTLVSFTIISLGAMSIFKKLDIGSLELADYLAIGAIFACVQILFAHC------------
AhNHX1          VG-----------AMIFFKKMDIGSLDLIDYLGI--------YLLCIMVLNQDETPLLYS
AtNHX1          VGTIISCTIISLGVTQFFKKLDIGTFDLGDYLAIGAIFAATDSVCTLQVLNQDETPLLYS
OsNHX1          VGTMISFFTISIAAIAIFSRMNIGTLDVGDFLAIGAIFSATDSVCTLQVLNQDETPFLYS
GmNHX3          ALGTLVVFIITLGATQIFKRLDVGPLELGDFLAIGAIFAATDSVCTLQVLNQDETPLLYS
BvNHX2          IGVFISTTIISTGSWWLFPKVGFAGLTARDYLAIGTIFSSTDTVCTLQILNQDETPFLYS
BvNHX3          LGTLISFCLVSSGVLLLLKKIGLTQLSVNDYMALGAILSATDSVCTLQVLNQDETPLLYS
AtNHX3          -----DRYKFYLENLTLGIRESRKEDEGWYLMTLEKNVSVQRFCLQLRLYEQVSTPEIKV
                                :  . .         : :

BvNHX1          LVFGEGVVNDATSVVLFNAIQSFDLTHIDHRIALQFSGNFLYLFFASTLLGAMTGLLSAY
SeNHX1          LVFGEGVVNDATSVVLFNAIQNFDLTNIDHRIAIQFSGNFLYLFFASTMLGAMTGLLSAY
SsNHX           LVFGEGVVNDATSVVLFNAIQNFDLTHIDHRIAFQFGGNFLYLFFASTLLGAVTGLLSAY
CqNHX1          LVFGEGVVNDATSVVLFNAIQSFDLTTIDHRIALQFMGNFLYLFIASTILGAFTGLLSAY
SoNHX1          ------------------------------------------------------------
AhNHX1          LVFGEGVVNDATSVVLFNALKSFDLSKIDHRIALTFMGNFLYLFFTSTVLGAMVILL---
AtNHX1          LVFGEGVVNDATSVVVFNAIQSFDLTHLNHEAAFHLLGNFLYLFLLSTLLGAATGLISAY
OsNHX1          LVFGEGVVNDATSIVLFNALQNFDLVHIDAAVVLKFLGNFFYLFLSSTFLGVFAGLLSAY
GmNHX3          LVFGEGVVNDATSVVLFNAIQSFDLNQIDPSIAGHFFSGNFLYLFFASMLGVLTGLLSAY
BvNHX2          LVFGEGVVNDATSVVLFNAIQKLNVKSFG--DWTHVIGDFLYLFFASTALGITTGLLTAY
BvNHX3          VVFGEGVVNDATSIVLFNAVQKLDLSNLSAMTALALLGTFLYLLVTSTLLGIFAGLLSAF
AtNHX3          LNKTQ--ENGTCTLILGCTVEKGD------------------------------------

BvNHX1          IIKKLYFGRHSTDREVALMMLMAYLSYMLAELFYLSGILTVFFCGIVMSHYTWHNVTESS
SeNHX1          VIKKLYFGRHSTDREVALMMLMAYLSYMLAELFYLSGILTVFFCGIVMSHYTWHNVTESS
SsNHX           VIKKLYFGRHSTDREVALMMLMAYLSYMLAELFYLSGILTVFFCGIVMSHYTWHNVTESS
CqNHX1          IIKKLYFGRHSTDREVALMMLMAYLSYMLAELFYLSGILTVFFCGIVMSHYTWHNVTESS
SoNHX1          ------------------------------------------------------------
AhNHX1          ---------HSTDREVALMMLMAYLSYMLAELFYLSGILTVFFCGIVMSHYTWHNVTESS
AtNHX1          VIKKLYFGRHSTDREVALMMLMAYLSYMLAELFDLSGILTVFFCGIVMSHYTWHNVTESS
OsNHX1          IIKKLYIGRHSTDREVALMMLMAYLSYMLAELLDLSGILTVFFCGIVMSHYTWHNVTESS
GmNHX3          IIKKLYIGRHSTDREVALMMLMMAYLSYLAELSYLSGILTVFFCGIVMSHYTWHNVTESS
BvNHX2          ALKALYFGRHSTDREIALMTLMAYLSYMLAELSQLSGILTVFFCGIVMSHYAWHNITENS
BvNHX3          VIKKLYIGRHSTDREIALMMLMAYLSYMIAELMELSGILTVFFCGVVMSHYTWHNVSESS
AtNHX3          -------------------------------------------------HVAYS------

BvNHX1          RVTTKHAFATLSFVAEIFLFLYVGMDALDIEKWRFVSDSPGTSIAVSSILIGLVMVGRAA
SeNHX1          RVTTKHAFATLSFVAEIFLFLYVGMDALDIEKWRFVSDSPGTSVAVSSILLGLLMVGRAA
SsNHX           RVTTKHAFATLSFVAEIFIFLYVGMDALDIEKWRFVSDSPGTSVAVSSILLGLHMVGRAA
CqNHX1          RVTTKHAFATLSFVAEVFLFLYVGMDALDIEKWRFVSDSPGTSVAVSSILLGLIMVGRAA
SoNHX1          ------------------------------------------------------------
AhNHX1          RITTKHAFATLSFVAETFLFLYVGMDALDIEKWKFVSDSPGTSVAVSSILLGLVMVGRAA
AtNHX1          RITTKHTFATLSFLAETFIFLYVGMDALDIDKWRSVSDTPGTSIAVSSILMGLVMVGRAA
OsNHX1          RVTTKHAFATLSFIAETFLFLYVGMDALDIEKWEFASDRPGKSIGISSILLGLVLIGRAA
GmNHX3          RITTKHSFATLSFVAEIFIFLYVGMDALDIEKWKFVVSDSPGSVATSSVLLGLILLVGRA
BvNHX2          RITTRHIFATMSFIAETFIFLYVGTDVFDMEKWKMTKMGFGTLVGIYTTVVFLILLGRAA
BvNHX3          RITSKHAFATMSFISETFIFLYVGMDTLDIDKWKESSTSAGTYLAVSTTMLALVLIGRAA
AtNHX3          --------------------------------WSEKAGTHPLNPANSSHLLSLTLGPQHA
```

```
BvNHX1          FVFPLSLLMNLSKKSHSEKVTFNQQV----------VIWWAGLMRGAVSMALAYNQFTRS
SeNHX1          FVFPLSLLINFSKKSHSEKITFNQQI----------VIWWAGLMRGAVSMALAYNQFTRS
SsNHX           FVFPFAFLMNLSKKSNSEKVTFNQQI----------VIWWAGLMKSAVSVALAYNQFSRS
CqNHX1          FVFPLSWLMNFAKKSHSEKVSLNQQV----------VIWWAGLMRGAVSMALAYNQFTRS
SoNHX1          ------------------------------------------------------------
AhNHX1          FVFPISLLTNFVKKSSSEKITVKQQASFYHAYSNIIIIWWAGLMRGAVSMALAYNQFTKG
AtNHX1          FVFPLSFLSNLAKKNQSEKINFNMQV----------VIWWSGLMRGAVSMALAYNKFTRA
OsNHX1          FVFPLSFLSNLTKKAPNEKITWRQQV----------VIWWAGLMRGAVSIALAYNKFTRS
GmNHX3          AFFPLSFLSNLAKKSPNEKISFFNQQ----------IVWWAGLMRGAVSIALAYNQFTMS
BvNHX2          FVFPLSAISNRMNREKENSGRRSTITFR-----KQVIIWWAGLMRGAVSIALAFKQFTYS
BvNHX3          FVFPLASILNYTRRGNDQKIDLSRQF----------IVWWAGLMRGAVTVALSYNAFSGS
AtNHX3          DNIYICTVSNPISNNSQTFSP------------------WPGCRTDPS------------


BvNHX1          GHTQLRGNAIMITSTISVVLFSTMVFGLLTKPLISFLLPHPKHFTSASTVSDMG----SP
SeNHX1          GHTQLRGNAIMITSTITIVLFSTMVFGLLTKPLILFLLPHSKHFNSASTVSDLG----SP
SsNHX           GHTQLRGNAIMITSTITVVLFSTMVFGLLTKPLILFMLPQPKHFTSASTVSDLG----SP
CqNHX1          GHTQLRGNAIMITSTITVVLFSTMVFGLLTKPLIMFLLPQPKHFTSCSTVSDLG----SP
SoNHX1          ------------------------------------------------------------
AhNHX1          GHTQLRGNAIMITTTISIVLFSTMVFGLLTKPLILFLLPQPKHFTSASTVSDLG----SP
AtNHX1          GHTDVRGNAIMITSTITVCLFSTVVFGMLTKPLISYLLPHQN--ATTSMLSDDN----TP
OsNHX1          GHTQLHGNAIMITSTITVVLFSTMVFGMMTKPLIRLLLPASGHPVTSEPSSPKS----LH
GmNHX3          GHTSLRSNAIMITSTITVVLFSTVVFGLLTKPLIRLLLPHTPHHKESSITITTDPSTPSP
BvNHX2          GITMDTVNATMVTTTVIVVLFTTLVFGFLTKPLILYLVPHTPTQETKSPKDDLT------
BvNHX3          GETVSNERSLMISCTIIVVLFSTLLFGTVTKPLIKAILGAPRRAVSDATDIPSL------
AtNHX3          ------------------------------------------------------------


BvNHX1          KSFSLPLLEDRQDSEADMGN--------YEESTNRSIPRPGSLRMLLNAPTHTVHFYWRK
SeNHX1          KSLSLPLLDGGQDSEADMDNQDEAVNNRYEGTHNRTIARPGSLRMLLNAPTHTVHYYWRK
SsNHX           KSFSLPLLEDRQDSEADLGNDDE------EAYPRGTIARPTSLRMLLNAPTHTVHHYWRR
CqNHX1          KAYSPLLEGHQDYEVDVGNHDNG---------TEPTIVRPSSLRMLLNAPTHTVHHYWRK
SoNHX1          ------------------------------------------------------------
AhNHX1          KSFIFPLLDGQDHEVNNDDVTDR------------NLIHPNSLRMLLNAPTHTVHYYWRK
AtNHX1          KSIHIPLLDQDSFIEPSGNHNVP---------------RPDSIRGFLTRPTRTVHYYWRQ
OsNHX1          SPLLTSMQGSDLESTTNIVR-------------------PSSLRMLLTKPTHTVHYYWRK
GmNHX3          KSVTVPLLGSAQESEVDIDGHDIHR--------------PSSIRAL--------------
BvNHX2          ----LPMLSMDESAATNLSRAKD------------------SLSMLIERPVHTIHSYWRK
BvNHX3          EYLDIPFLETPETNQNNGDDPER---------------QRRGFSLLMKYPTGAVHHIWRK
AtNHX3          ------------------------------------------------------------
```

Supplementary Figure S8. Alignment of NHXs protein sequences. The multiple sequence alignment of NHXs protein sequences was generated with ClustalW (http://www.ebi.ac.uk/Tools/msa/ClustalW2/). Identical amino acids are marked with an asterisk (*) below. Protein accession numbers and species names used for amino acid sequences alignment are showed on Supplementary Table S1.

```
CqHAK5          ------------MMDPESGLSQ-------------------------------IQEVKKK
AhHAK5          -------------MMDPESGFY-------------------------------ENQVKKK
SeHAK5          -------------MMDPESGFY-------------------------------QNQLKK-
OsHAK25         --------MDLEAAHGAAAAPG-------------------------------KRRRRAR
BvHAK5          --------MPKSNVSSNNEQDSVNFENQGRPKLRRHDSLDLESSNVKGHHHVGGGHSAKD
SoHAK5          --------MPKSNISSNPDSDELHG---KTTRLRRHDSLDLESSNVKGHYAG-AGHFAKN
OsHAK1          --------------------------------LKRHDSLFGDAEKVS----GGKHHGGSA
OsHAK5          MTEPLHTSSNGGAERGPNAAFESEKTLQTTTRLQRFDSLHMEAGKIP----GGQSHT-AK
AtHAK5          ---MDGEEHQIDGDEVNNHENKLNEKKKSWGKLYRPDSFIIEAGQTP----TNTGRR-SL
SeHAK1          ------------------------------------------------------------


CqHAK5          ESWRQVLILAYQSLGVVYGDLSTSPLYVYKSAFA-EDIQHSETNEEIFGVLSFVFWTLTI
AhHAK5          ESWRQVLILAYQSLGVVYGDLSTSPLYVYKSTFA-EDIQHSESNDEIFGVLSFVFWTLTI
SeHAK5          ESWKQVLILAYQSLGVVYGDLSTSPLYVYKSAFA-EDIEHSDTSEEIYGVLSFVFWTLTI
OsHAK25         ESWGASLLLAYQSLGVVYGDVATSPLYVYKSAFAGDDIQHSAGNEEIYGVLSFVFWTLTL
BvHAK5          ASWAVVLSLAFQSLGVIYGDIGTSPLYVYASTFT----DGIQDRDDILGVLSLIYYTITL
SoHAK5          ASWAVVLSLAFQSLGVIYGDIGTSPLYVFASTFT----DGIKHTDDILGVLSLIYYTITL
OsHAK1          VSWAVTLHLAFQSVGIIYGDIGTSPLYVYSSTFP----DGIGHRDDLVGVLSLILYTLII
OsHAK5          VGWATTLHLAFQSIGVVYGDMGTSPLYVFSSTFT----NGIKDTNDILGVMSLIIYTVVL
AtHAK5          MSWRTTMSLAFQSLGVVYGDIGTSPLYVYASTFT----DGINDKDDVVGVLSLIIYTITL
SeHAK1          ------MLSINNIVVNKYKHLCNSLYLIILHLFS------------SLHWISSKTYDFVI
                      :    : :   * .: .*   :    *.                :*   : . :

CqHAK5          VPLLKYVFIVLRADDNGEGGTFALYTLLCRHARVNSLPNCQLADEDLSEYKKDGVVIPSE
AhHAK5          VSLLKYVFIVLKADDNGEGGTFALYSLLCRHARVNSLPNCQLVDEELFEYKKD-VVVPIE
SeHAK5          IPLLKYVFIVLRADDNGEGGTFALYSLLCRHARVNSIPNCQLADEDLSEYKKD-GVVPAQ
OsHAK25         ISLVKYVLIVLRADDGGEGGTFALYSLICRHVRAGLLP-GGAGDELAVGGRRD-------
BvHAK5          LPVIKYVFIVLRANDNGKGGTFALYSLLCRNTKVGLLPSQQAEDQELSNYQLELPNKK-I
SoHAK5          IPVIKYVFIVLRANDNGKGGTFALYSLLCRNAKVGLLPSQQVEDQEVSNYQLDLPTKCNQ
OsHAK1          IPMLKYVFIVLYANDNGDGGTFALYSLISRYAKIRMIPNQQAEDAMVSNYSIEAPSSQ--
OsHAK5          LPLIKYCFIVLRANDNGDGGTFALYSLISRYARISLIPNQQAEDAMVSHYKLESPSNR--
AtHAK5          VALLKYVFIVLQANDNGEGGTFALYSLICRYAKMGLIPNQEPEDVELSNYTLELPTTQ--
SeHAK1          INISHFIIELCYFILVSSFGFLTLKTLNP-------------------------------
                : : :: : :      .. * ::* :*

CqHAK5          STIGSRLKSILEKYRVLQKLLLVLALIGTCMVIGDGVLTPAISVFSAVSGLELSMSHEHH
AhHAK5          SGFGSRLKSILEKYRVLQKLLLVLALLGTCMVIGDGVLTPAISVFSAVSGLELSMSHEHH
SeHAK5          TNFGSGLKSILEKHRVLQKLLLILALIGTCMVIGDGVLTPAISVFSAVSGLELSMSHEHH
OsHAK25         ARAMSRLRAMLERYRVLQRLLLLFALLGTCMVIGDGVLTPAVSVYSAVSGLELSMEHEHH
BvHAK5          ASVPLKLKSFLENSIISKYILLFVTMLATSLVIGDGVLTPSISVLSAISGLKAASPVFTE
SoHAK5          LGVSLKLKTFLENSKISKYILLIVTMLSTSLVIGDGVLTPSISVLSAIGGLKGASPVFTE
OsHAK1          LRRAQWVKHKLESSRAAKMALFFLTILGTSMVMGDGTLTPAISVLSAVSGIREKAPNLTQ
OsHAK5          VKRAHWIKEKMENSPNFKIILFLVTILATSMVIGDGVLTPCISVLSAVGGIKESAKSLTQ
AtHAK5          LRRAHMIKEKLENSKFAKIILFLVTIMGTSMVIGDGILTPSISVLSAVSGIKS----LGQ
SeHAK1          -KSIHKNDKININNPIKDLDLFFTSVSATTVSSMSTLEMEVFSNSQ--------------
                 .  *:. :: .* :   .      .*  .

CqHAK5          KYVEVPVACIILIGLFALQHFGTHRVGFLFAPIVVTWLFCISAIGLYNIIHWNPHVYQAL
AhHAK5          KYVEVPIACLILIGLFALQHFGTHRVGFLFAPIVVTWLFCISAIGLYNIIYWNPQVYQAL
SeHAK5          KYVEVPVACLILIGLFALQHYGTHRVGFLFAPIVVAWLFCISSIGLYNIIYWNPHIYQAL
OsHAK25         KYVQLPVTCAILIGLFALQHYGTHRVGFIFAPIVCVWLLCISAIGVYNIVHWNHHVYRAL
BvHAK5          GRIVW-ISVGILILLFSAQRFGTDKVGYSFAPIVSVWFLFNAGIGIYNFIKYDPTVIKAV
SoHAK5          GRVVW-ISIGILILLFSAQRFGTDKVGYSFAPIVTVWFLFNAGIGVYNFAKYDPTVIKAV
OsHAK1          TQVVL-ISVAILFMLFSVQRFGTDKVGYTFAPIISVWFLLIAGIGLYNLVVHEITILKAF
OsHAK5          GQIAG-IAIAILIVLFLVQRFGTDKVGYSFGPIILTWFIFIAGTGVYNLFKHDTGVLKAF
AtHAK5          NTVVG-VSVAILIVLFAFQRFGTDKVGFSFAPIILVWFTFLIGIGLFNLFKHDITVLKAL
SeHAK1          -------LIVITILMFIGGEVFTSMIGLHFSASNLVYKPLKSRSRVNSVASLTLPFEPIE
                          * : :*   .  *  :*  *..   .:        : ..      .

CqHAK5          SPYYMYKFLKKTQTGGWMSLGGILLCITGSEAMFADLGHFSQSSIKIAFTFLVYPSLILA
AhHAK5          SPYYMYKFLKKTQAGGWMSLGGILLCITGSEAMFADLGHFSQLSIKIAFSFVVYPSLILA
SeHAK5          SPYHMYKFLKKTQTGGWMSLGGILLCITGSEAMFADLGHFSQLSIKIAFSFVVYPSLILA
OsHAK25         SPYYMYQFLKKTQTGGWMSLGGILLCVTGSEAMYADLGHFSQSSIKIAFMSVVYPALVLA
BvHAK5          NPWYIVQYFQRNGRNAWISLGGIFLCVTGTEALFADVGHFSVKSIQISMSFVTYPALMLT
SoHAK5          NPWYIVQYFRRHGKDAWISLGGVFLCITGTEALFADVGHFSVKSIQLSMSFVTYPALMLT
OsHAK1          NPWYIVQYFRRNGKKGWVSLGGVVLCVTGTEGMFADLGHFNIRAVQISFNCILFPSVALC
OsHAK5          NPKYIVDYFERNGKQGWISLGGVILCITGTEAMFADLGHFNVRAIQIGFSVVLLPSVLLA
AtHAK5          NPLYIIYYFRRTGRQGWISLGGVFLCITGTEAMFADLGHFSVRAVQISFSCVAYPALVTI
SeHAK1          LGLIVVTPQENNTS-------------TPTMQKTKSETDFFIKSKSIRVLGFLVLLYLFT
                    :  ..                  * :     .  .*    : .: .  .

CqHAK5          YMGQAAYLSKHHVMATSYQIGFYVSVPEKLRWPVLGIAILAAVVGSQAIITGTFSIIKQC
AhHAK5          YMGQAAYLSKHHVMASGYRIGFYVSVPG--------------------------------
SeHAK5          YMGQAAYLSKHHVVATDYRIGFYVSVPETLRWPVLIIAILAAVVGSQAIITGTFSIIKQC
OsHAK25         YMGQAAYISQHHSFENAYHIGFYVSVPEKLRWPVLVIAILAAVVGSQAVITGTFSIIKQC
BvHAK5          YSGQASFLRMNQNLVSNT---FYEAIPGPLYWPMFVVAILATIVASQAMISGTFSIIQQS
SoHAK5          YSGQASFLRLHQNQVKDT---FYDCIPGPLYWPMFVVAVLATIVASQAMISGTFSIIQQS
OsHAK1          YIGQAAYLRKFPENVSDT---FYKSIPGPLFWPTFIVAILAAIIASQAMLSGAFAILSKA
OsHAK5          YIGQAAYLRIYPEHVADT---FYKSIPDPLYWPTFVVAVAAAIIASQAMISGAFAIIAQS
AtHAK5          YCGQAAYLTKHTYNVSNT---FYDSIPDPLYWPTFVVAVAASIIASQAMISGAFSVISQS
SeHAK1          IHILGISMVLLYLNITPN-----------------AINVLHKKGIETFTFSIFTIVSTF
                    .  :
```

```
CqHAK5          QALGCFPRVKIVHTSSKIHGQIYIPEINWTLMILCLAVTIGFRDTKRMGNASGLAVITVM
AhHAK5          -----------------------------------------------------LAVITVM
SeHAK5          QALGCFPRVKIVHTSSKIHGQIYIPEINWTLMILCLAVTIGFRDTKRMGNASGLAVITVM
OsHAK25         SSLSCFPGVKIVHTSSTVHGQIYIPEINWILMILCLAVTLGFRNTKHLANAQGLAVITVM
BvHAK5          LALGCFPRVKVVHTSAKYEGQVYIPELNYILMLSCVAVTVGFRTTENIGHAYGIAVALAE
SoHAK5          LALGCFPRVKVVHTSAKYEGQVYIPEINYFLMLACVGVTLGFRTTENIGHAYGIAVAFAE
OsHAK1          LSLGCLPRVRVIHTSKKYEGQVYIPEVNFMMGLASIIVTIAFRTTTSIGNAYGICVVTTF
OsHAK5          QILGCFPRVRVIHTSTKFHGQVYIPEINYVLMVLCVAVTAIFQTTDKIGNAYGIAVVFVM
AtHAK5          LRMGCFPRVKVVHTSAKYEGQVYIPEINYLLMLACIAVTLAFRTTEKIGHAYGIAVVTVM
SeHAK1          ASCGFIPTNENMQVFSKNSG------------------------------FLLILIPQIL
                                                                     : :

CqHAK5          LVTTCLMSLVIVICWRKSVFVALAFVFFFGAFEALYFSASLIKFLEGAWVPVALSLIFMA
AhHAK5          LVTTCLMSLVIVLCWRKSVFIALAFVIFFGAFEALYFSASLIKFLEGAWVPIALSLIFMF
SeHAK5          LVTTCLMSLVIVLCWRKSVFVALAFVLFFGAFEALYFSAALIKFLEGAWVPVALSLIFMI
OsHAK25         LVTTCLMSLVIVLCWNKSIFLALGFLIFFGTIEVLYFSASLVKFHEGAWVPITLSFIFMI
BvHAK5          SITSSFMILVMLIIWKKHIILVIIYVVCIMSVELLYLSSLLYKFPHGGYFPIAFASVFLV
SoHAK5          TITSAFMVLVMLVIWKKHIALVILYVCCIWSVELIYLSSVLYKFPHGGYFPIALASVFLA
OsHAK1          MVTTHLMTVVMLLIWKKHLVFILLFYCVFGFTEVVYLSSILSKFVDGGYLPFCFAMVLMT
OsHAK5          FITTLLVTLVMVMIWKTSLLWIALFPVIFGGAELIYLSSAFYKFTQGGYLPLVFSAILMF
AtHAK5          VITTLMVTLIMLVIWKTNIVWIAIFLVVFGSIEMLYLSSVMYKFTSGGYLPLTITVVLMA
SeHAK1          LGNTLFPSFLRFSIWVLGKFAKKDEAKYLLRNAKEIGYHHLLPSKHSRLLIMTVLGFIMV
                  .: :  .: .  *             :           :     .  . . .  .::

CqHAK5          IMYIWHYGTLKKYEFDVQNKVSINWLLSLGPSLG-IVRVRGMGLIHTELVSGIPAIFSHF
AhHAK5          IMYAWHYGTLKKYEYDVQNKVSINWLLSLGPSLG-IVRVRGIGLIHSELVSGIPAIFSHF
SeHAK5          IMYVWHYGTRKKYEFDVQNKVSINWLLSLGPSLG-IVRVRGIGLIHTELVSGIPAIFSHF
OsHAK25         VMCVWHYGTIKKYEFDFQNKVSVNWLLNLGPSLG-IVRVRGIGLIHTELVSGIPAIFSHF
BvHAK5          VMAIWNYVYRKKYYYETENKVSPNTLKDVANQSN-CSRIPGLAIFYSKLVDGIPPIFGHY
SoHAK5          LMVIWNYVYRKKYYYEMDNKISPNTLRDIANQTN-MSRIPGLAIFYSELVDGIPPIFGHY
OsHAK1          MMATWHYVHVRRYWYELDHIVPTAELASLLEENGGVRRVPGVGLLYTELVQGIPPLFPRL
OsHAK5          IMATWHYVHVHRYKYELRNKVSNNYVAELAVKQN-LARLPGIGFLYSELVQGIPPILPHL
AtHAK5          MMAIWQYVHVLKYRYELREKISRENAIQMATSPD-VNRVPGIGLFYTELVNGITPLFSHY
SeHAK1          QLVVFCAMEWSNEGINKGHNVYQKLVGILFQCVN--SRHTGESIVDLSSIAPAMLVVFIL
                 :  :      .   :  . :       :    .   *  * .:.  . :     :.

CqHAK5          VTNLPAFHQVLVFLCVKSVPVPHVKPEERFLVGHIGPKEYRLYRCIVRYGYRDFHKDDFE
AhHAK5          VTNLPAFHQVLVFLCVKSVPVPHVKPEERFLVGHIGPKEYRLYRCIVRYGYRDFHKDDFE
SeHAK5          VTNLPAFHQVLVFLCVKSVAVPHVRPEERFLVGHIGPKEYRLYRCIVRCGYRDFHKDDFE
OsHAK25         VTNLPAFHQVLVFLCVKSVPVPHVQPEERFLVGRIGPKEYRLYRVIVRYGYRDVQKDDIE
BvHAK5          VDNIPALHSVLVFASVKSLPVSTVPPEERFLFRRVYPKELYVFRCVVRYGYTDARNEEEP
SoHAK5          VENISVLHSVLVFASVKSLPISTVPAEERFLFRRVHPRELYVFRCVVRYGYTDARNEEEP
OsHAK1          VRKIPSVHAVFVFISIKHLPIPHVAAAERFLFRQVGPRARRVFRCVARYGYTDALEEPRE
OsHAK5          VEKVPSIHSVLVIISIKYLPISKIETKERFLFRYVEPKEYRVFRCVVRYGYNDKVEDPAE
AtHAK5          ISNLSSVHSVFVLISIKTLPVNRVTSSERFFFRYVGPKDSGMFRCVVRYGYKEDIEEPDE
SeHAK1          MMYLPPYTSFVPIK-----------DEEKEYTHILCKEEDKRRKILKNVLFSQLSHLAI
                :  :.  ..   :              *.     :  :     * : :    .  ..

CqHAK5          FENDLVCSVAEFIRGEGNKMN--GLKDESDKDSDEKMTVVGSPSTHVDGIRMHEDMDEDN
AhHAK5          FENDLVCSIAEFIRAEGTKMN--GIKAESDKESNEKMTVVGSPSTYVDGIRMHEDIEHN-
SeHAK5          FENDLVCSVAEYVRAESSKVNENGFKDESEKDHDERMTVVGSPSTYADGIKMHEDEVEVE
OsHAK25         FEKDLVSSIAEFIRSGDSHHN--GVLEDTDKSCEKLSSISNGIPLWME------------
BvHAK5          FERMLIEQLKEFMRDDFRFQA---QMEHSPPRVEHEMEDDQSDITHDNG-----------
SoHAK5          FERLLIDRLKEFIKDEFWVYT---QKDHPLSLQHVQEHESSDHIHEGDHNIENTLTRAN-
OsHAK1          FAAFLVDGLKMFIQEESAFAP---HQEMIDAAADDDDEAAARPRRSTSSAVHSEEAI---
OsHAK5          FESLVIENLKQFIHEESLYSQ---SSHSLEGESIKEIGGVTDPTSEVQDAMSSRNNS---
AtHAK5          FERHFVYYLKEFIHHEHFMSG---GGGEVDETDKEEEPNAETTVVPSSNYVPSSGRIGSA
SeHAK1          FTIIICIIEKQNMKDDPLNFN---------------------------------------
                *   .       ::

CqHAK5          ----EDLPGTSELKEIRSPLTTPTPKKRVRFLLPETPKMEDKSREELQDLMEAREAGIAY
AhHAK5          ----EDLPGTSELKEIRSPIITPK--KKVRFLLPESPKMGDGAKEELQDLMEAREAGIAY
SeHAK5          VDNHQDLPGTSEVREIRSPVTSTAK-KRVRFSLPESPKMENVSREELKDLMEAREAGIAY
OsHAK25         ---DGEVDASASPHKETDTQIISPNRKKARFVLPKNAQVDSEVRRELQELMDAREAGMSF
BvHAK5          ----------------------------------------ALEREIEVVDKALKAGVVH
SoHAK5          ---------------------------------------EEALEKEIEVVDKAWNSGVVH
OsHAK1          ----------------QAASSGRTTASSVQLQAGGEPPAAMDVEEEKRLIDREVGRGVVY
OsHAK5          ----------------DQHTTEPRNGCMDEIQS----------------IHKEMGNGVVH
AtHAK5          ---------------HSSSSDKIRSGRVVQVQS---------VEDQTELVEKAREKGMVY
SeHAK1          ----------------------------------------VFNIAFEVISAYGNVGFST
                                                                 :      *.
```

```
CqHAK5          ILGHAHVRAKAGSSWVKKLVINVGYDFLRRNCRAPSYPLSVPHASTLEVGMICHI-
AhHAK5          ILGHAHVRAKQGSSWIKKVVINIGYDFLRRNCRAPSYPLSVPHVSTLEVGMICHI-
SeHAK5          ILGHAHVKAKNGSNWLKKLVINFGYDFLRRNSRAPSYPLSVPHASTLEVGMVCHI-
OsHAK25         ILGHSYMKAKSGSSFIKRIVINFFYEFLRRNSRGPSYAATIPHASTLEVGMVYQV-
BvHAK5          MMGESEVMANKGAGIGKKIVIDTVFSILKRNLRASDKMFDIPRKRLIKIGMTYEL-
SoHAK5          MMGESQVVANKGASIGKKIVIDFAFNFMKKNLRQSDKMFDIPRKRLLKVGMTYEL-
OsHAK1          LMGEANVSAGPNSSILKRIAVNYIYTFLRKNLTEGHRALAIPNDQLLKVGITYEI-
OsHAK5          LLGETNVVAEPNADFLKKIIVDYVYNFIRKNFRQPEKITCVPHNRLLRVGMTYEI-
AtHAK5          LMGETEITAEKESSLFKKFIVNHAYNFLKKNCREGDKALAIPRSKLLKVGMTYEL-
SeHAK1          GYSCGKQLKGDPKCVEKWYGFAGRWSDQGKLALIIVMFFGRLKKFNLKGGKAWKLL
                  .             *   .   :    :            .   :. *   .:
```

Supplementary Figure S9. Alignment of HAK protein sequences. The multiple sequence alignment of HAK protein sequences was generated with ClustalW (http://www.ebi.ac.uk/Tools/msa/ClustalW2/). Identical amino acids are marked with an asterisk (*) below. Protein accession numbers and species names used for amino acid sequences alignment are showed on Supplementary Table S1.

```
AhSKOR        MTPKQLAKIISGKRMSFNREEGS----------TSSNSNHEEDFVIEKPRDRLKSSRGSR
ApSKOR        MTPKQLARMISGKKIMSRNREGGDGDGDERGEGSTSSSNKEEDFVIERPRDRMKSSRGSR
CqSKOR        MKPKQIARIMMTRGREERMEGSTS----------SSSSNREEDFMVERPRERLYASVGSR
BvSKOR        MTPKKLVRLISGKTMSTRREEGEEVEG------STSPSNHKRDYTVATPRERLKSSRGKQ
SoSKOR        MTPKKIVRLISGKTMSWNREEGNEERSS----TSPSSSKDEEDYMVERPRERMKSSRVKQ
AtSKOR        --------------------MGGSSGGGVSYRSGGESDVELEDYEVDDFRDGIVESRGNR
OsSKOR        ---------------------------------------MAEEYELNEIDDTLHGSVGSR
                                                       .:: :    : :  *  .:

AhSKOR        FDLLVHELNHESPERQFSREGFFHGVKDLYNGLAIHPENNRWYRAWDKFILLWAIYSSFF
ApSKOR        FDLLVNELNHESSQRKFTREGFFLGCKDLYHGLVIHPENNRWFKAWDKFILLWAIYSSFF
CqSKOR        FNLLVTEFSHESSQRQFTREGFFVGVKDLYHGLVIHPEHNRWYKAWDNIILLWAIYSSFF
BvSKOR        FDRMVNELNQESSQREFCREGFFNGIVDLYHGLAIHPDNNRWYRAWEKFILLWAIYSSFF
SoSKOR        FDMLVNELKHESTPREFCREGFFTGLTDLYHGLVIHPEKNRWFKAWDKFILLWAFYSSFF
AtSKOR        FNPLTNFLGLDFAGGSGGKFTVINGIRDISRGSIVHPD-NRWYKAWTMFILIWALYSSFF
OsSKOR        LSLFARELKSRRSSSWHGG-TALRLPKDLYESLVIHPN-GRWYRIWANMMFLWSIYSTFF
              :. :.  :    .         :    *: ..  :**: .**:: *  ::::*::**:**

AhSKOR        TPMEFGFFNGLPENLFVLDIVGQVAFLLDIVLQFFVGYRDKQTYRMVCQRPAIAFRYLKS
ApSKOR        TPMEFGFFRGLPENLFVLDIVGQVAFLMDIVLQFFVAYRDKQTYRMVYQRPAIAFRYLKS
CqSKOR        TPMEFGFFRGLPENLFVLDIVGQVAFLMDIVLQFFVGYRDKQTYRMVYQRPAIAFRYLKS
BvSKOR        TPLEFGFFTGLPKNLLVLDLVGQVAFLVDIVLLFYVGYRDRKTHHMVHQRRAIAFRYLKS
SoSKOR        TPLEFGFYRGLPENLFILDIAGQIAFLVDIVLGFFVAYRDKQTYRMIHHRRPIAFRYLKS
AtSKOR        TPLEFGFFRGLPENLFILDIAGQIAFLVDIVLTFFVAYRDSRTYRMIYKRSSIALRYLKS
OsSKOR        TPFEFSFFRGLPDQLLDLECV-QLVFLADVAVHFFLAYRDPHTYRMVHDKRHIALRYIKG
              **:**.*: ***.:*: *: . *:.** *:.: *::.*** :*::*: .:  **:**:*.

AhSKOR        TFVIDLLACLPWDLIYKASGHKEAVRYLLWIRLCRVRKVHYFLQKMEKDIRINYLFTRII
ApSKOR        TFVVDLLACLPWDLIYKASGHEEAVRYLLWIRLCRVRKVHYFLQKMEKDIRINYLFTRIV
CqSKOR        TFVLDLLACLPWDLIYKASHRKEAVRYMLWIRLCRVRKIHFFLQKLEKDIRINYLFTRIV
BvSKOR        TFMLDLLACMPWDLIYKAAKRKEAMRYLLWIRLCRVRKITYFLHKLEKDIRINYLFTRIV
SoSKOR        TFFVDLLACMPWDLIFKASHRKEIVRYLLWIRLCRVRKITFFLQKMEKDIRINYLFTRII
AtSKOR        TFIIDLLACMPWDIIYKAAGEKEEVRYLLLIRLYRVHRVILFFHKMEKDIRINYLFTRIV
OsSKOR        SFALDVLGCFPWDAIYKVTGRVEAVRWLVWVRLYRGRKVMAFFKRVEKDIRVSYLLTRIV
              :* :*:*.*:*** *:*.: . * :*::: :** * :::  *::::*****:.**:***:

AhSKOR        KLVTVEIYCTHTAACIFYYLATTVPEREEGYTWIGSLTLGDYSYAHFREIDIWRRYTTSL
ApSKOR        KLLTVELYCTHTAACIFYYLATTLPEKEEGYTWIGSLTLGDYSYSHFREIDIWRRYTTSL
CqSKOR        KLLTVELYCTHTAACIFYYLATTIPEREEGYTWIGSLTLGDYSYSHFREIDLWRRYTTSL
BvSKOR        KLLVVEIYCTHTAACIFYYLATTVPEREEGYTWIGSLTLGDYSYSNFREIDIWKRYTTSL
SoSKOR        KLLTVELYCTHTAACIFYYLATTIPEQEEGYTWIGSLKLGDYSYSHFREIDLFRRYTTSL
AtSKOR        KLIFVELYCTHTAACIFYYLATTLPASQEGYTWIGSLKLGDYSYSKFREIDLWTRYTTSM
OsSKOR        KLITVELYCTHTAACGFYYLATTLPPAREGGTWIGSLSLGDARYINFREVDLLTRYVTSL
              **: **:******** *******:*  .** ******.***  * :***:*:  **.**:

AhSKOR        YFAIVTMATVG---YGDIHAVNMREMIFIMIYVSFDMVLGAYLIGNMTALIVKGSKTERF
ApSKOR        YFAIVTMATVG---YGDIHAVNMREMIFIMIYASFDMVLGAYLIGNMTALIVKGSKTERF
CqSKOR        YFAIVTMATVG---YGDIHAVNMREMIFIMIYVSFDMILGAYLIGNMTALIVKGSKTERF
BvSKOR        YFAIVTMATVG---YGDIHAVNLREMIFIMIYVSFDMILGAYLIGNMTALIVKGSKTEKF
SoSKOR        YFAIVTMATVG---YGDIHAVNLREMIFIMIYVSFDMILGAYLIGNMTALIVKGSKTERF
AtSKOR        YFAVVTMATVG---YGDIHAVNMREMIFAMVYISFDMILGAYLIGNMTALIVKGSKTERF
OsSKOR        YLAIVTMATVGGSGYGDIHAVNTREMAFTVVYISFSIVLSAYLIGNMTALIVKGSRTERF
              *:*:*******   ******** *** * ::* **.::*.***************:**:*

AhSKOR        RDKMAEVMKYMNRNRLSRDLRNQIKGHLRLQYESSYT-EASVLQDIPISLRAKVIR----
ApSKOR        RDKMAEVMKYMNRNRLSRDLRNQIKGHLRLQYESSYT-EASVLQDIPVSLRAKISQTLYM
CqSKOR        RDKMTEVMKYMNRNRLSRDLRNQIKGHLRLQYESSYT-EASVLQDIPISLRAKISQSLYM
BvSKOR        RDKMTDVMKYMNRNRLERGLCNQIKGHLRLQYESSYT-EASILRDIPISLRAKIFQSLYR
SoSKOR        RDKTAEVMKYMYRNRLDKDLRNKIKGHLRLQYESSYT-EAAVLQDIPISLRAKISHTLYM
AtSKOR        RDKMADIMRYMNRNKLGRNIRGQITGHLRLQYESSYT-EAAVLQDIPVSIRAKIAQTLYL
OsSKOR        RDRMTDLIRYMNRNRLGSAIRSQVKDHLMLQYESSYTRDRVIVDDIPVAVRSKMSQTLYL
              **: :::::** **:*   : .::..** ******** :  :: ***:::*:*: :

AhSKOR        -----------------------VHEEFFLPGEVILEQGNVVDQLYFVCHGMLEEIGIGE
ApSKOR        PYIEKASLFSGCSSEFINQIVIRVHEEFFLPGEVILEQGNVVDQLYFVCHGMLEEIGVGE
CqSKOR        PYIEKVPLFKGCSPEFINQIAIRVHEEFFLPGEVILEQGNVVDQLYFICHGLLEEVGIGQ
BvSKOR        PYIEESPLFNGCSSEFINQIVIRVHEEFFLPGEVILEQGNVVDQLYVVCHGMLEEVSIGQ
SoSKOR        PFIEKAPLFKECSPEFKNQIVTKVHEEFFLPGEVILEQGNVVDQLYLVCHGKLEEVSIGQ
AtSKOR        PYIEKVPLFRGCSSEFINQIVIRLHEEFFLPGEVIMEQGSVVDQLYFVCHGVLEEIGITK
OsSKOR        DMVSRVGLFRGCSDDFLSQIVLKLHEEFFLPGEVILEQGTVVDQIYIVAHGCLEEVANGE
                                     :***********:***.****:*.:.** ***:.  :

AhSKOR        HGSEQTISILERNSTFGQISIFCNIPQPSTVRVLELCRLLRIDKESLSNIIDIYFYDGKK
ApSKOR        DGSEQTITLLEPNNTFGQISIFCNIPQPTTVRVLELCRLLRIDKQSLSNTIDIYFYDGKR
CqSKOR        DGSEQIISRLEPNNTFGQISIFCNVPQPSIIRVIDLCRLLRIDKESLSNIIDIYFYDGKK
BvSKOR        DGSEMKVSILQPNSTFGQISIFCNIPQSCTVRVLDLCRLLRIDKESLSNIIDIYFNDGKI
SoSKOR        DGSEQLISRLEPDSTFGQNSIFCNIPQSCTVRVVDLCRLLRIDKQSLSNIIDIYFFDGKK
AtSKOR        DGSEEIVAVLQPDHSFGEISILCNIPQPYTVRVAELCRILRLDKQSFMNILEIFFHDGRR
OsSKOR        DGSEEIISELRPYGIVGDVAVICNIPQPYTVRVCELCSLLRIDKQSLTSILQIYFKDNSQ
              .***  :: *.    .*: :::**:**.  :** :** :**:**:*: . ::*:* *.
```

```
AhSKOR          IFDNLLEGNDG-KYNLKQVESDIASHIAKQESELALKVNNAAYHGDLYQLKSLLNAGADL
ApSKOR          IFDNLLEGND--KYNLKQVESDIASHIAKQESELALKVNNAAYHGDIYQLKNLLNAGADL
CqSKOR          IFDNLLEGNNNGKYNLKQVESDIASHIAKQESELALKVNNAAHHGDLYQLKSLLNAGADL
BvSKOR          IFNNLLKGNDG-KYNLSQVESDIASHIRKQESELALKVNNVAYHGDLYQLKTLINGGADL
SoSKOR          IFDNLLKGNDG-KFNLTEVESDIASHITQQESELALKVNNAANHGDLYQLKSLLKAGADL
AtSKOR          ILNNLLEGKES-NVRIKQLESDITFHISKQEADVALKLNSAAFYGDLYQLKSLIRAGGDP
OsSKOR          ILSNLLKGKET-ESKRKQLESDITYLLAKQESELVLGVNNAAYHGDIFRLKSLISAGADP
                *:.***:*::  : . .::****:  : :**:::.* :*..* :**:::**.*: .*.*

AhSKOR          KKTDYNGRSPLHIAASRGYEDIVVFLIEEGADVNKSDNFGNTPLLEAVKNGHDQVAALLY
ApSKOR          KKTDYNGRSPLHIAASRGHEEIVRFLIEEGADVNKSDNFGNTPLLEAVKNGHDEVAATLY
CqSKOR          KKTDYNGRTPLHIAASGGCEDIVLFLIEEGADVNRTDNFGNTPLLEAVKNGHDKVAALLY
BvSKOR          KRKDYNGRVPLHVAASRGHEAIVLFLIQEGADVNILDDFGNTPLLEAVKNGHDEVAALLY
SoSKOR          NKTDYNERSPLHIAASKGHENIVLFLIQAGALVNQSDNFGNTPLLEAAKNGHDQIAAVLY
AtSKOR          NKTDYDGRSPLHLAASRGYEDITLYLIQESVDVNIKDKLGSTPLLEAIKNGNDRVAALLV
OsSKOR          SKSDYDGRTALHIAALRGYENIVRFLIQRGANVNSIDRFGNSPLLQAVKSGHDRITSLLV
                .:.**: * .**:**  * * *. :**: .. **  * :*.:***:* *.*:*.::: *

AhSKOR          REGAKIDVKDVGSFLCTVVLSG---DVEFLKRIVANGIDPNSKDYDHRTPLHVACSQGLF
ApSKOR          KQGAIIDVKDVGSFLCTVVLRG---DVEFLKRIIANGIDPNSKDYDQRTPLHVACSQGLF
CqSKOR          KEKARIDVKDVGSFLCTVVLSG---DVEFLKRILTNGMDPNSKDYDHRTPLHVACSQGLY
BvSKOR          KEGARIDLKNAGSSLSTIVLSG---DVEVLRRMITNGVDPNSRDYDLRTPLHVACSQGLY
SoSKOR          REGARIDIKDVGTYLCTVVLSKESKDVDFLKRLLNNGVDPNSRDYDGRTPLHVACSHGLY
AtSKOR          KEGATLNIENAGTFLCTVVAKG---DSDFLKRLLSNGIDPNSKDYDHRTPLHVAASEGFY
OsSKOR          EHGAILNLEDAGGYLCRVVRGG---RIDLLKKLLRFGISPNCRNYDQRTPLHIAAAEGLH
                .. * :::::.*  *. :*        :.*::::  *:.**.::** *****:*.:.*:.

AhSKOR          LMAKVLVEAGAFVTLKDRWGNTPLDEAWNCGNKHLIKLLEAAKSAQLSQSSGNLEELLDK
ApSKOR          LMAKVLVDAGAYVTLKDRWGNTPLDEAWMCGNKHLIKLLEAAKSAQLSQSSGNIEEMLDK
CqSKOR          LMAKLLVEAGAYVTLKDRWGNTPLDEAWMCGNKNLIKLLEAAKAAQLAQSSDHIEEMLDK
BvSKOR          LMAKLLVEAGANVLLKDRQGKTPMDEARMCGHNNLIKLLEDAKLAQLSQSSADIEDMSDK
SoSKOR          LMAKLLVEAGALVLLKDRWDNTPLDEARLCGHNNLIKLLEAAKSAQLSQPAGQIEQISDR
AtSKOR          VLAIQLVEASANVLAKDRWGNTPLDEALGCGNKMLIKLLEDAKNSQISSFPSGSKEPKDK
OsSKOR          LVASTLIESGADIQAKDRWGNTPLDEGRRCSSKPLVRILEQARTVATN------------
                ::*  *:::.* :  *** .:**:**.  *. : *:::** *:

AhSKOR          TLHKKCTVFPFHPWGSKENRKPGIVLWVPRTIEQLVKIASEKLNFPTGSCILSDDGGKIL
ApSKOR          KLHKKCTVFPFHPWGPKENRKPGIVLWTPHRIDELIKMASEKLNFPRGSSILSEDGGKIL
CqSKOR          KLHKKCTVFPFHPWGTKENRKPGIVLWLPRTIDELIRLASEKLNFLTGSCILSEDGGKIL
BvSKOR          TLHKKCIVFPFHPWETKENRRTGIVLWVPRTIDELVKIASEKLNFPTGSCILSDDGGKIL
SoSKOR          KLHKKCTVFPVHPWDTQEKRIPGIVLWIPRTIDELVKTASEKLNLPTATCVLSEDGGKIL
AtSKOR          VYKKKCTVYFSHPGDSKEKRRRGIVLWVPRSIEELIRTAKEQLNVPEASCVLSEDEAKII
OsSKOR          ------------------------------------------------------------


AhSKOR          DVDMIDNGQKLYLISETE
ApSKOR          DVDMIDNGQKLYLISETE
CqSKOR          DVDMIENGQKLYLISETE
BvSKOR          DVDLVDNGQKLYLISKTE
SoSKOR          DIEMIDNGQKLYLITETD
AtSKOR          DVDLISDGQKLYLAVET-
OsSKOR          ------------------
```

Supplementary Figure S10. Alignment of SKOR protein sequences. The multiple sequence alignment of SKOR protein sequences was generated with ClustalW (http://www.ebi.ac.uk/Tools/msa/ClustalW2/). Identical amino acids are marked with an asterisk (*) below. Protein accession numbers and species names used for amino acid sequences alignment are showed on Supplementary Table S1.

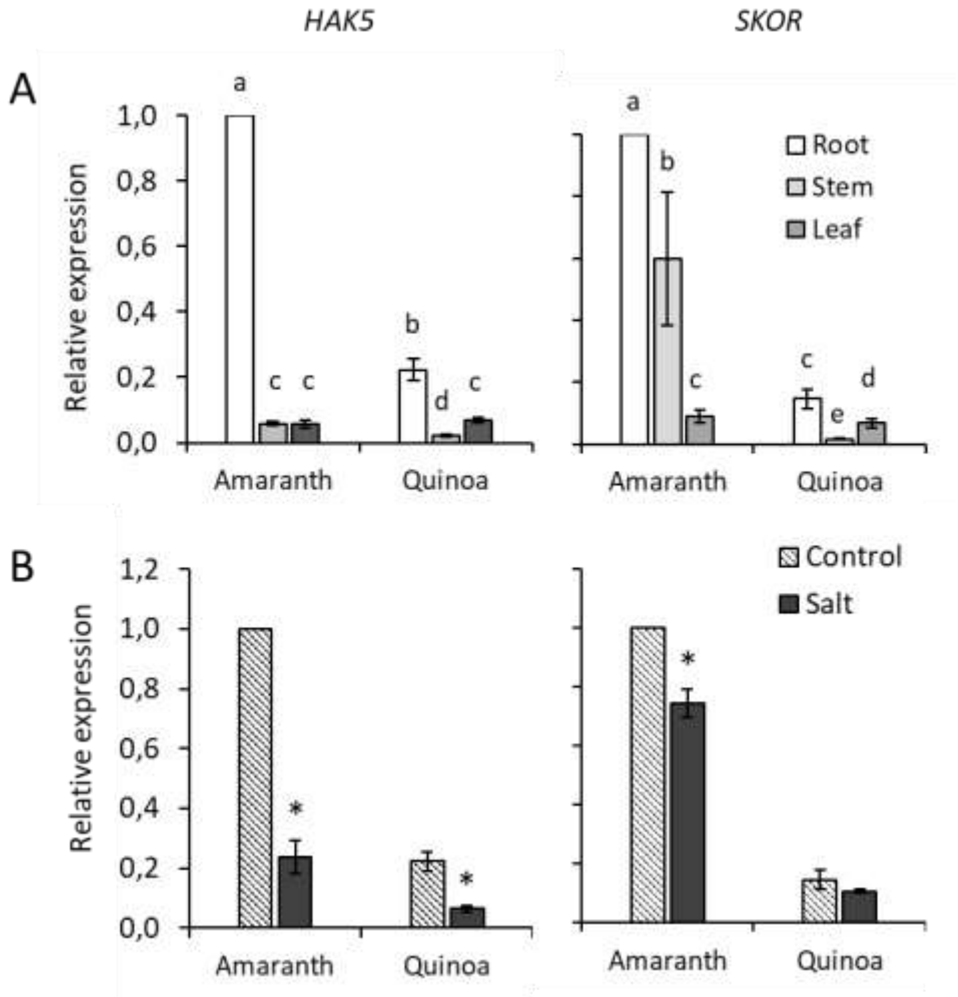

Supplementary Figure S11. Constitutive and relative expression of genes involved in K^+ homeostasis in amaranth and quinoa. (A) Comparative constitutive expression of *HAK5* and *SKOR* genes analyzed in root, stem and leaf of plants grown in control condition. (B) Changes in relative expression levels of genes involved in K^+ homeostasis (*HAK5* and *SKOR*) in root of amaranth and quinoa plants grown in control and salt stress (100 mM NaCl) for 20 days. The expression of root in control was set to 1 for amaranth. Values are means ± SE of three biological replicates (each one of 3 plants, n=9). Different letters indicate significant differences among mean values of constitutive expression levels of *HAK5* and *SKOR* genes (A) (LSD test, $p \leq 0.05$). Asterisks indicate significant differences between control and salt treatments in (B) (Student's *t*-test, $p \leq 0.05$).

Supplementary Table S1. Protein accession numbers and species of genes used in phylogenetic relationships, alignments and design of the degenerate primers.

Name	Species	Protein Accession Number	Sequence Source
AtSOS1	*Arabidopsis thaliana*	NP_178307.2	Gen Bank
OsSOS1	*Oryza sativa*	XP_015619351.1	Gen Bank
SpSOS1	*Sesuvium portulacastrum*	AFX68848.1	Gen Bank
McSOS1	*Mesembrythemum crystallinum*	ABN04858.1	Gen Bank
AhSOS1	*Amaranthus hypocondriacus*	AH006272-RA	Phytozome
BvSOS1	*Beta vulgaris*	XP_010680103.1	Gen Bank
SoSOS1	*Spinacia oleraceae*	CDL70805.1	Gen Bank
CqSOS1	*Chenopodium quinoa*	XP_021727954.1	Gen Bank
SjSOS1	*Suaeda japonica*	BAE95196.1	Gen Bank
SdSOS1	*Salicornia dolichostachya*	CDL70804.1	Gen Bank
SbSOS1	*Salicornia brachiata*	ACJ63441.1	Gen Bank
OsHKT1;2	*Oryza sativa*	ANF99165.1	Gen Bank
BvHKT1;1	*Beta vulgaris*	XP_010690257.1	Gen Bank
CqHKT1;1	*Chenopodium quinoa*	XP_021776291.1	Gen Bank
AhHKT1;1	*Amaranthus hypocondriacus*	AHYPO_003592-RA	Phytozome
McHKT1;1	*Mesembrythemum crystallinum*	AAK52962.1	Gen Bank
CqHKT1;2	*Chenopodium quinoa*	XP_021774566.1	Gen Bank
BvHKT1;2	*Beta vulgaris*	XP_010690256.1	Gen Bank
AtHKT1;1	*Arabidopsis thaliana*	NP_567354.1	Gen Bank
OsHKT1;1	*Oryza sativa*	QHR93066.1	Gen Bank
OsHKT1;3	*Oryza sativa*	CAD37185.1	Gen Bank
OsHKT2;1	*Oryza sativa*	BAB61789.1	Gen Bank
OsHKT1;4	*Oryza sativa*	CAD37197.1	Gen Bank
OsHKT1;5	*Oryza sativa*	AFY08297.1	Gen Bank
McHKT1;2	*Mesembrythemum crystallinum*	AAO73474.1	Gen Bank
BvHKT1;3	*Beta vulgaris*	XP_010688439.1	Gen Bank
SsHKT1;1	*Suaeda salsa*	AAS20529.2	Gen Bank
SbHKT1;1	*Salicornia bigelovii*	ADG45565.1	Gen Bank
AtNHX3	*Arabidopsis thaliana*	AAG10433.1	Gen Bank
GmNHX3	*Glycine max*	AFC91768.1	Gen Bank
OsNHX1	*Oryza sativa*	LOC_Os07g47100.1	Phytozome
BvNHX3	*Beta vulgaris*	XP_010675005.1	Gen Bank
BvNHX2	*Beta vulgaris*	XP_010675850.1	Gen Bank
AtNHX1	*Arabidopsis thaliana*	NP_198067.1	Gen Bank
CqNHX1	*Chenopodium quinoa*	XP_021742352.1	Gen Bank
SoNHX1	*Spinacia oleraceae*	CDL70807.1	Gen Bank
AhNHX1	*Amaranthus hypochondriacus*	AHYPO_008765-RA	Phytozome
SsNHX1	*Suaeda salsa*	AAK53432.1	Gen Bank
BvNHX1	*Beta vulgaris*	XP_010672472.1	Gen Bank
SeNHX1	*Salicornia europaea*	AAN08157.1	Gen Bank
SeHAK5	*Salicornia europaea*	ADP95697.1	Gen Bank
OsHAK5	*Oryza sativa*	XP_015622303.1	Gen Bank
OsHAK1	*Oryza sativa*	CAD20991.1	Gen Bank

AtHAK5	*Arabidopsis thaliana*	NP_567404.1	Gen Bank
OsHAK25	*Oryza sativa*	Q6YWQ4.1	Gen Bank
CqHAK5	*Chenopodium quinoa*	XP_021726984.1	Gen Bank
SeHAK5	*Salicornia europaea*	ADP95697.1	Gen Bank
AhHAK5	*Amaranthus hypochondriacus*	AHYPO_013402-RA	Phytozome
BvHAK5	*Beta vulgaris*	XP_010670701.1	Gen Bank
SoHAK5	*Spinacia oleracea*	XP_021843968.1	Gen Bank
OsSKOR	*Oryza sativa*	Os04g36740	Phytozome
AtSKOR	*Arabidopsis thaliana*	CAA11280.1	Gen Bank
CqSKOR	*Chenopodium quinoa*	XP_021754949.1	Gen Bank
AhSKOR	*Amaranthus hypochondriacus*	AHYPO_001358-RA	Phytozome
ApSKOR	*Alternanthera philoxeroides*	AFO70199.1	Gen Bank
SoSKOR	*Spinacia oleracea*	XP_021845325.1	Gen Bank
BvSKOR	*Beta vulgaris*	XP_010670208.1	Gen Bank

Supplementary Table S2. Sequences of primers for RT-qPCR gene expression analysis used in this study.

AMARANTH		
**Oligo name**	**Oligo sequence, 5' to 3'**	**Efficiency (%)**
Ah_*EF1a*_Fw	AGGATGGTCAAACCCGTGAG	91.20%
Ah_*EF1a*_Rv	TGTCTGGGCTGTAACCAACC	
Ah_*SOS1*_Fw	TGGCCTGCACGAGAAAATTC	91.00%
Ah_*SOS1*_Rv	TCTCGAGGACTGTTCGTGGA	
Ah_*HKT1*;1_Fw	GGAGCTCTGGAGTTATGGAGG	100.40%
Ah_*HKT1*;1_Rv	ACAACCAAGATGGCCGAACT	
Ah_*NHX1*_Fw	CCAATGTTCCAACGGCTCCA	99%
Ah_*NHX1*_Rv	GGTTTAGCCACCGGAGTTGT	
Ah_*HAK5*_Fw	GCAAGCTGCCTACCTATCGAA	94%
Ah_*HAK5*_Rv	GGGCAATAGGAACCCAAGCT	
Ah_*SKOR*_Fw	ACCCGACACAATCTGATCCA	90%
Ah_*SKOR*_Rv	TGGTTTGTCAACGTCCTGCT	
**QUINOA**		
**Oligo name**	**Oligo sequence, 5' to 3'**	**Reference or Efficiency**
Cq_*EF1a*_Fw	GTACGCATGGGTGCTTGACAAACTC	(Böhm et al., 2018)
Cq_*EF1a*_Rv	ATCAGCCTGGGAGGTACCAGTAAT	
Cq_*SOS1*_Fw	TCATGAGCCATTATGTGA	
Cq_*SOS1*_Rv	TTACTTGCCTCGTCTTTA	
Cq_*HKT1*;1_Fw	CTTGAATTGGCCGTTTG	
Cq_*HKT1*;1_Rv	CGTTGTACCCCGTGTTCT	
Cq_*HKT1*;2_Fw	CTCTTCATTAGCTTCAAAC	
Cq_*HKT1;2*_Rv	AGAGGTAGGGTTAGGGT	
Cq_*NHX1*_Fw	TCCGTCACATTGTGCCATGT	(Ruiz-Carrasco et al., 2011)
Cq_*NHX1*_Rv	TGGCTTGCTCAGTGCTTACA	
Cq_*HAK5*_Fw	ATTGGGTCAAGGCTTAAA	(Böhm et al., 2018)
Cq_*HAK5*_Rv	AACATGTGGGTTCCAGTG	
Cq_*SKOR*_Fw	ACCCGACACAATCTGATCCA	104%
q_*SKOR*_Rv	TGGTTTGTCAACGTCCTGCT	
